# A hybrid pathway for self-sustained luminescence

**DOI:** 10.1126/sciadv.adk1992

**Published:** 2024-03-08

**Authors:** Kseniia A. Palkina, Tatiana A. Karataeva, Maxim M. Perfilov, Liliia I. Fakhranurova, Nadezhda M. Markina, Louisa Gonzalez Somermeyer, Elena Garcia-Perez, Marta Vazquez-Vilar, Marta Rodriguez-Rodriguez, Victor Vazquez-Vilriales, Ekaterina S. Shakhova, Tatiana Mitiouchkina, Olga A. Belozerova, Sergey I. Kovalchuk, Anna Alekberova, Alena K. Malyshevskaia, Evgenia N. Bugaeva, Elena B. Guglya, Anastasia Balakireva, Nikita Sytov, Anastasia Bezlikhotnova, Daria I. Boldyreva, Vladislav V. Babenko, Fyodor A. Kondrashov, Vladimir V. Choob, Diego Orzaez, Ilia V. Yampolsky, Alexander S. Mishin, Karen S. Sarkisyan

**Affiliations:** ^1^ Planta LLC, 121205 Moscow, Russia.; ^2^ Shemyakin-Ovchinnikov Institute of Bioorganic Chemistry, Russian Academy of Sciences, 117997 Moscow, Russia.; ^3^ Institute of Science and Technology Austria, 3400 Klosterneuburg, Austria.; ^4^ Instituto de Biología Molecular y Celular de Plantas (IBMCP), Consejo Superior de Investigaciones Científicas (CSIC), Universitat Politècnica de Valéncia, 46022 Valencia, Spain.; ^5^ Pirogov Russian National Research Medical University, Ostrovityanova 1, Moscow 117997, Russia.; ^6^ Lopukhin Federal Research and Clinical Center of Physical-Chemical Medicine of Federal Medical Biological Agency, Moscow, Russia.; ^7^ Okinawa Institute of Science and Technology Graduate University, Okinawa 904-0412, Japan.; ^8^ Botanical Garden of Lomonosov Moscow State University, Vorobievy Gory 1 b.12, Moscow 119234 Russia.; ^9^ Light Bio Inc., Ketchum, ID, USA.; ^10^ Synthetic Biology Group, MRC Laboratory of Medical Sciences, London, UK.; ^11^ Institute of Clinical Sciences, Faculty of Medicine and Imperial College Centre for Synthetic Biology, Imperial College London, London, UK.

## Abstract

The fungal bioluminescence pathway can be reconstituted in other organisms allowing luminescence imaging without exogenously supplied substrate. The pathway starts from hispidin biosynthesis—a step catalyzed by a large fungal polyketide synthase that requires a posttranslational modification for activity. Here, we report identification of alternative compact hispidin synthases encoded by a phylogenetically diverse group of plants. A hybrid bioluminescence pathway that combines plant and fungal genes is more compact, not dependent on availability of machinery for posttranslational modifications, and confers autonomous bioluminescence in yeast, mammalian, and plant hosts. The compact size of plant hispidin synthases enables additional modes of delivery of autoluminescence, such as delivery with viral vectors.

## Teaser

Pathway for substrate-free luminescence gets upgraded by identification of compact hispidin synthases in a range of land plants.

## INTRODUCTION

In the bioluminescent fungus *Neonothopanus nambi*, light emission results from the oxidation of polyketide 3-hydroxyhispidin (*1*). The discovery of all the genes encoding 3-hydroxyhispidin biosynthesis and recycling (*2*) allowed reconstitution of the pathway in heterologous hosts and engineering of organisms with self-sustained luminescence (*3*, *4*). As the fungal bioluminescence system does not require exogenous application of substrate and shows no toxicity to eukaryotes, it has the potential to become the foundational technology for a suite of autoluminescent reporter tools for longitudinal noninvasive physiology imaging (*4*, *5*).

**Fig. 1.**
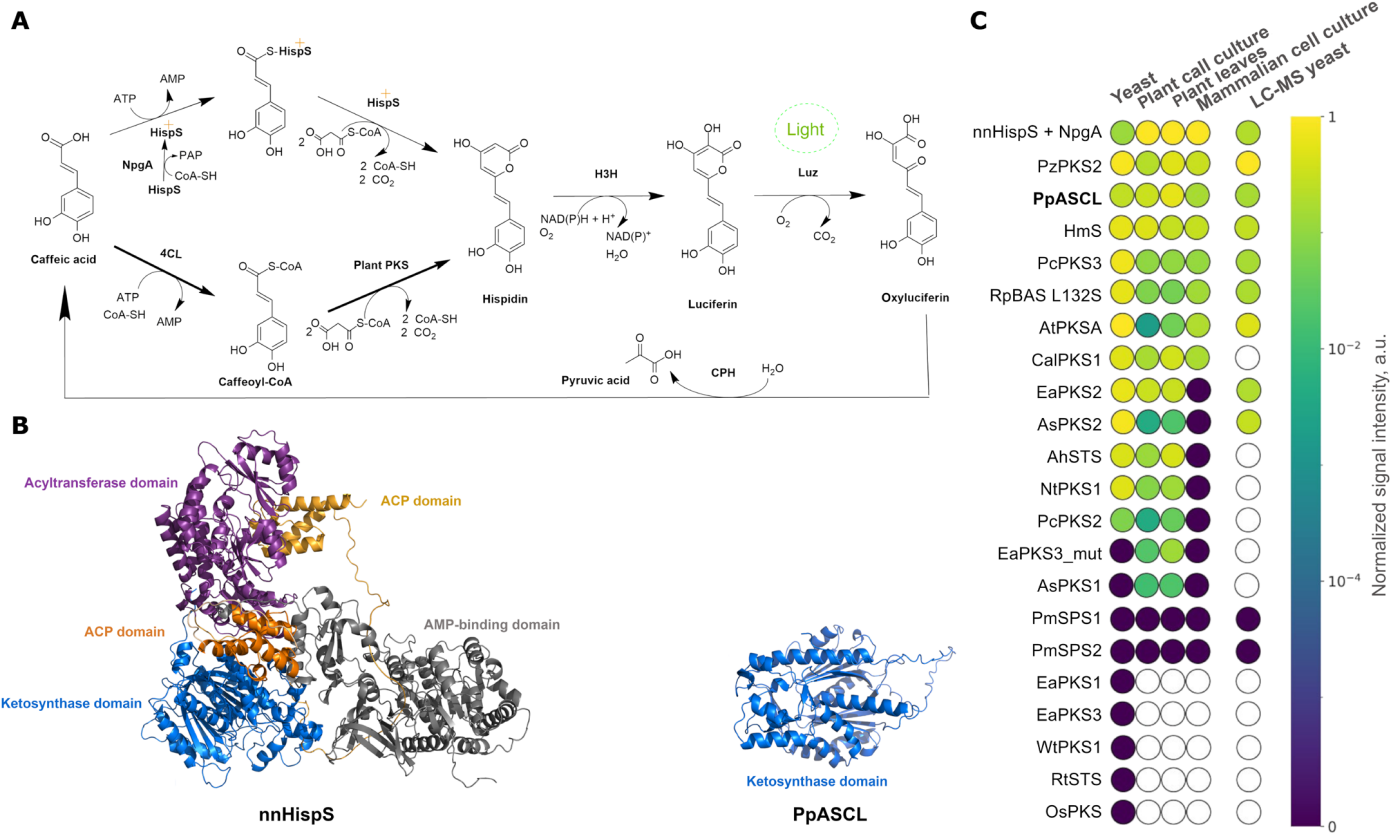
Fungal bioluminescence system with plant polyketide synthases. (**A**) Caffeic acid cycle catalyzed by nnLuz, nnH3H, nnCPH, and nnHispS (with NpgA) from *N. nambi* or plant PKS (with 4CL). (**B**) Protein structures of nnHispS and a plant polyketide synthase (PpASCL), predicted by AlphaFold 2.0 (*52*). (**C**) Comparison of luminescence conferred by expression of plant PKS genes in various hosts and relative hispidin concentration in yeast. White space indicates enzymes not included in the corresponding experiment. AMP, adenosine 5′-monophosphate; ATP, adenosine 5′-triphosphate; NAD(P)^+^, nicotinamide adenine dinucleotide (phosphate); NAD(P)H, reduced form of NAD(P)^+^; ACP, acyl carrier protein; a.u., arbitrary units.

The wild-type pathway from *N. nambi*, however, may need reengineering before it becomes a widely useful tool for imaging studies. One of the problems is the size of the genetic construct. Four enzymes are essential for reconstitution of the pathway in organisms comprising caffeic acid (Fig. 1A). The first reaction of the pathway, biosynthesis of hispidin, is carried out by a type I polyketide synthase—nnHispS [~5.1–kilo–base pair (kbp) gene]. HispS requires a posttranslational modification—addition of phosphopantetheinyl group—for its activity (*6*). Thus, in heterologous hosts, coexpression with phosphopantetheinyl transferase, such as NpgA from *Aspergillus nidulans*, is typically needed (Fig. 1A) (*3*, *4*). This further increases the number of transcription units to at least five. As a result, the size of the DNA encoding bioluminescence exceeds 12 to 17 kbp for most eukaryotic hosts, depending on the length of regulatory elements. In addition, the 5.1-kbp length of the *hisps* gene alone makes it incompatible with size-sensitive delivery systems, such as delivery with RNA viruses (cargo capacity of ~4 kbp) (*7*) or geminiviruses (increasing cargo size decreases replication efficiency) (*8*, *9*) for plant expression (*10*) or adeno-associated viruses (cargo capacity of ~4.7 kbp) for expression in animal systems (*11*, *12*).

In contrast to large enzymes characteristic for fungi, polyketide production in land plants relies on compact type III polyketide synthases (*13*, *14*). Unlike HispS, these enzymes accept coenzyme A (CoA)–esters as substrates and thus may require coexpression with CoA ligases but are about four times smaller (Fig. 1C) and do not need posttranslational phosphopantetheinylation (*13*). Land plants are known to produce hispidin and related α-pyrones, and caffeoyl-CoA is a ubiquitous plant metabolite (*15*, *16*). Thus, if a hispidin synthase was found in plants, then it could potentially catalyze hispidin biosynthesis and replace both HispS and phosphopantetheinyl transferase in the fungal bioluminescence pathway. However, no genes encoding hispidin synthases have been identified in plants. In 1997, Beckert and colleagues (*17*) reported hispidin synthase activity in extracts of the horsetail *Equisetum arvense* but did not identify the corresponding gene. In addition, several enzymes have been reported to catalyze synthesis of hispidin-like molecules from CoA-esters of hydroxycinnamic acids in vitro (*13*).

## RESULTS

In this work, we aimed to search for hispidin synthases of plant origin that could be used in the *N. nambi* bioluminescence pathway. In the literature describing heterologous expression of type III polyketide synthases, we looked for the presence of styrylpyrones in the chromatography data (*18*–*24*). Following the work by Beckert *et al.* (*17*), we also screened publicly available transcriptomes of *E. arvense* (*25*) and selected several candidate type III polyketide synthases. Our shortlist included 21 genes from phylogenetically diverse plant species covering bryophytes, horsetails, magnoliids, monocots, and dicots (table S1 and figs. S1 and S2), which we cloned and evaluated for ability to produce hispidin.

We initially assayed hispidin synthase activity in the yeast *Pichia pastoris* by testing whether the enzymes could restore light emission in strains expressing other genes essential for bioluminescence (coumarate-CoA ligase 4CL1, hispidin-3-hydroxylase nnH3H, and luciferase nnLuz; Fig. 1A and table S2). Twelve of 21 selected candidates restored light emission when caffeic acid was applied exogenously, with 11 outperforming the fungal enzyme nnHispS (Fig. 1C). We also assayed production of polyketides by yeast strains coexpressing just the polyketide synthase and coumarate-CoA ligase by liquid chromatography–mass spectrometry (LC-MS). LC-MS analysis showed production of hispidin as one of the major products by the strains encoding “light-emitting” candidates, confirming that land plants from diverse phylogenetic groups encoded hispidin synthases (figs. S3 and S4).

The brightest luminescence was observed from the strain encoding PzPKS2 from *Plumbago zeylanica* (Fig. 1C and figs. S5 to S8): Depending on the concentration of substrate, these cells emitted one to two orders more light than the strain that coexpressed the fungal hispidin synthase and a phosphopantetheinyl transferase. No light was emitted from colonies expressing PmSPS1 and PmSPS2, although these enzymes were previously reported to produce hispidin as one of their products in vivo (*16*).

We then tested whether plant hispidin synthases can functionally replace the fungal synthase and phosphopantetheinyl transferase in mammalian and plant transient expression systems: Human embryonic kidney (HEK) 293NT human cells, BY-2 plant cell culture (*26*), and *Nicotiana benthamiana* leaves (*27*)*.* We detected light emission from 14 candidate hispidin synthases in plant cells (Fig. 1C and fig. S9) and from 7 in mammalian cells (Fig. 1C and figs. S10 and S11). Three enzymes—PzPKS2, HmS, and PpASCL—appeared as top candidates across plant, yeast, and mammalian systems, with PpASCL demonstrating the brightest luminescence in plants (figs. S12 and S13).

We benchmarked the brightness of the top-performing pathway variants against NanoLuc and firefly luciferase, two commonly used nonautonomous bioluminescence reporters. In yeast cells (fig. S14), all versions of the autoluminescence pathway, except the all-fungal version, were brighter than the firefly luciferase. HmS-based pathway emitted 40 times more light than the firefly luciferase and was 12 times dimmer than NanoLuc over the 1-hour acquisition period. In plant cells (fig. S15), autonomous luminescence conferred by the brightest HmS-based hybrid pathway was 24-fold dimmer than that of NanoLuc and 7-fold dimmer than that of firefly luciferase. The kinetics of luminescence curves were different, with peak differences reaching two orders of magnitude (figs. S14B and S15B).

We then assessed whether compact size of plant synthases could enable delivery of the bioluminescence pathway with size-sensitive vectors. We tested a size-sensitive RNA-based [tobacco mosaic virus (TMV)] and a non–size-sensitive DNA-based [bean yellow dwarf virus (BeYDV)] replicative viral systems in plant leaves (figs. S16 and S17). As expected, in the case of BeYDV, both all-fungal and hybrid pathways produced clear bioluminescence signals. In the case of TMV, the large size of the fungal hispidin synthase did not allow TMV replication and movement, resulting in no luminescence from infiltrated leaves. In contrast, all three tested plant-derived polyketide synthase genes produced easily detectable and mobile bioluminescence when delivered with TMV. This result illustrated the utility of the hybrid bioluminescence pathway for size-sensitive applications.

**Fig. 2.**
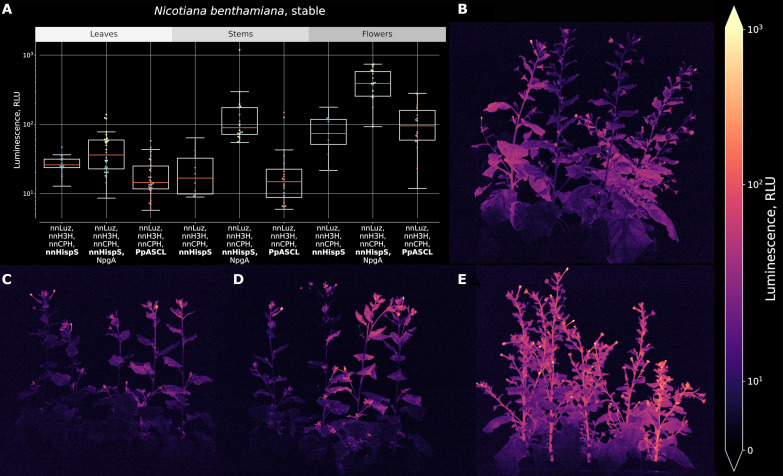
Transgenic *N. benthamiana* plants expressing different versions of the bioluminescence pathway. (**A**) Average brightness of leaves, stems, and flowers in 9- to 10-week-old plants. (**B**) Photo of transgenic plants producing nnHispS, nnLuz, nnH3H, and nnCPH (line NB021)*.* (**C** and **D**) Photo of transgenic lines NB221 (C) and NB220 (D) producing PpASCL, nnLuz, nnH3H, and nnCPH. (**E**) Photo of transgenic plants producing nnHispS, NpgA, nnLuz, nnH3H, and nnCPH (line NB2359). RLU, Relative Luminescence Units.

To validate the activity of plant hispidin synthases in vivo, we created transgenic *N. benthamiana* lines (NB218, NB220, and NB221) coexpressing PpASCL, nnLuz, nnH3H, and nnCPH. We observed sustained autonomous bioluminescence with signal strengths suitable for bioluminescence imaging. Lines expressing plant hispidin synthases were glowing at brightness levels similar to those reported by Mitiouchkina *et al.* (*3*) but were typically more than an order of magnitude dimmer than the representative line coexpressing fungal hispidin synthase nnHispS and NpgA (Fig. 2 and fig. S18). We hypothesized that dimmer luminescence of PpASCL-expressing plants may indicate lower concentration of the functional hispidin synthase, its lower affinity to substrates compared to competing endogenous enzymes, or inhibition by reaction products or other plant metabolites and set out to test these hypotheses.

In vivo concentration of caffeic and coumaric acids in plant leaves is reported to vary within 10 to 100 μM range (*3*, *28*). To test how concentration of pathway intermediates affects luminescence, we injected caffeic acid, coumaric acid, hispidin, and luciferin into autonomously glowing lines. Injections of hispidin and luciferin into PpASCL-expressing lines resulted in a significant increase in brightness of bioluminescence (fig. S19), indicating that hispidin biosynthesis was the bottleneck of the pathway in these lines. Injections of caffeic or coumaric acid did not increase luminescence, indicating that these metabolites were not limiting the pathway in planta.

Consistent with this interpretation, we also observed no significant differences in caffeic and coumaric acid content of plant leaves transiently expressing plant or fungal polyketide synthases, compared to control samples (fig. S20, A and B). Hispidin content measured in the same samples followed the expected pattern: no hispidin in the control leaves, highest content in nnHispS-expressing leaves, and lower content in the PpASCL-expressing leaves (fig. S20C).

We supplemented these data by perturbing phenylpropanoid metabolism with CRISPR-based activators. Using previously validated plasmids, we activated expression of 11 genes involved in phenylpropanoid metabolism in *N. benthamiana* [phenylalanine ammonia-lyase 1 (*PAL1*), *PAL2*, *4CL*, cinnamate-4-hydroxylase (*C4H*), chalcone synthase (*CHS1*), *CHS2*, chalcone-flavonone isomerase 1 (*CHI1*), *CHI2*, flavonol synthase (*FLS*), dihydroflavonol 4‐reductase (*DFR*), and flavonoid 3’‐hydroxylase (*F3’H*) individually and in combinations (fig. S21) (*29*) but did not detect a change in luminescence compared to the dummy guide RNA control in any of the samples (figs. S22 to S25). Similarly, we observed no change in luminescence upon overexpression of genes involved in production of phenylpropanoids—*C4H* and malonyl-CoA synthetase *AAE13* (figs. S21 and S26 to S28). Together, these results suggested that in transgenic plants, hispidin biosynthesis was limited by the enzymatic activity and not by the supply of precursor molecules.

To rule out that conversion of caffeic acid to caffeoyl-CoA was constraining light emission, we overexpressed 4-coumarate:CoA ligase Pv4CL1 from *Panicum virgatum (**30**)*. Overexpression of Pv4CL1 did not change light emission (fig. S29). This result indicated that caffeoyl-CoA biosynthesis was not the limiting step. In the nnHispS-expressing line, Pv4CL1 reduced light emission, likely due to competition for substrate with nnHispS, demonstrating that Pv4CL1 was functional.

In contrast, we found that overexpression of PpASCL itself in the background of genomically expressed PpASCL resulted in brighter luminescence (fig. S29). We thus concluded that PpASCL products did not inhibit downstream luminescence enzymes and that it was the low activity of PpASCL that limited the pathway. Plant polyketide synthase activity was reported to be regulated by 26*S* proteasome–mediated degradation (*31*), enzyme recruitment into metabolons (*32*), and inhibition by plant metabolites (*33*–*35*). Injection of proteasome inhibitor MG132 into leaves of glowing tobacco lines did not increase luminescence, indicating that degradation was unlikely the reason for low activity (fig. S30). Another potential explanation was that PpASCL efficiently produced hispidin solely when overexpressed, while at physiological expression levels, the enzyme was sequestered in metabolons isolating it from relevant substrates or changing product specificity (*32*). To test this hypothesis, we shortlisted six genes involved in assembly or function of phenylpropanoid metabolons [*PAL4,* *CHI*, chalcone-flavonone isomerase–like protein (*CHIL*), *CHIL3*, *CYP90A1*, and flavanone 3-hydroxylase (*F3H*)] (fig. S19) (*36*) and induced their silencing via RNA interference but did not observe a significant change in luminescence of PpASCL-expressing lines (figs. S31 to S33).

Thus, we believe that reduced activity of PpASCL in transgenic plants is likely due to inhibition by endogenous plant metabolites. Products of the flavonoid pathway such as naringenin, naringenin chalcone, luteolin, and apigenin, including those present in tobacco (*37*), were reported to inhibit chalcone synthase homologs (*33*–*35*); and regulation of type III polyketide synthases by flavonoids was proposed as a mechanism to avoid accumulation of metabolites at levels toxic for the plant (*38*). Catalytic triad of type III polyketide synthases is different from that of ketosynthase domains of type I enzymes (*39*). It is thus not unexpected that compared to their fungal analog, these enzymes may be differentially inhibited in vivo by numerous phenylpropanoids present in *N. benthamiana* (*37*).

## DISCUSSION

To summarize, in this work, we identified a set of compact hispidin synthases encoded by a phylogenetically diverse group of land plants. Among our candidates, enzymes from dicotyledonous plants varied in their ability to produce hispidin, while tested enzymes from monocots and two enzymes from magnoliid plants did not produce hispidin. The highly conserved enzyme responsible for the biosynthesis of sporopollenin in bryophytes, PpASCL, was among the most active hispidin-producing enzymes we found.

When expressed in yeast, some of polyketide synthases demonstrate high activity, outperforming the fungal hispidin synthase in the bioluminescence pathway by more than an order of magnitude. In mammalian cells, the performance of both all-fungal and hybrid pathways remains suboptimal and requires further optimization. In transgenic plants, hispidin synthase PpASCL shows lowered activity compared to data in yeast, likely due to inhibition by endogenous plant metabolites (figs. S7, S12, and S13). Observed differences in enzyme activity in various heterologous hosts, including enzymes that failed to yield light emission, might reflect differences in protein stability at different temperatures, availability, and concentration of inhibitors, as well as host-specific targeting to subcellular localization and other factors.

Autoluminescence pathways have the potential to become universal tools to report physiological events at the organism level. This includes noninvasively monitoring changes in activity of genes, spread of infections, or computation in synthetic genetic circuits. A clear downside of autonomous luminescence as a reporter technology is the dependence of the signal on both gene expression and metabolic activity of the host (*3*). Multistep branched metabolic pathways may have multiple bottlenecks that change dynamically depending on physiological and environmental inputs (*40*, *41*). Replacement of the fungal hispidin synthase by PpASCL in the bioluminescence cycle anchors the bottleneck of the pathway to expression of the hispidin synthase. PpASCL-based pathway appears tolerant to metabolic perturbations (figs. S21 to S28). We thus expect that in planta, the hybrid pathway will be useful for longitudinal bioluminescence imaging experiments, where circadian rhythms, plant development, and other physiological factors influence activity of phenylpropanoid metabolism. At the same time, reduction in the size of the largest gene of the pathway from 5.1 to 1.2 kbp enabled viral gene delivery of the bioluminescence pathway, making new types of autoluminescence experiments possible.

## MATERIALS AND METHODS

### Assembly of DNA

Coding sequences of *nnLuz*, *nnH3H*, *nnCPH*, *nnHispS*, *NpgA*, *At4CL1*, *Pv4CL1*, and plant polyketide synthases were optimized for expression for both *P. pastoris* and *N. benthamiana* and ordered synthetically (tables S1 and S4).

We used Golden Gate assembly for all plasmids created in this study. The assembly followed the modular cloning standard described in (*42*). For yeast expression, genes were cloned into plasmids with varied selectable markers under control of promoter glyceraldehyde-3-phosphate dehydrogenase promoter pGAP and alcohol oxidase terminator tAOX.

For experiments in plants, genes were cloned into MoClo-like level 1 or level M vectors for agrobacterium-mediated transformation. All genes were cloned under control of constitutive promoters of viral origin (p35S-nnLuz-tAct2; pFMV-nnH3H-tNOS, p35S-nnCPH-tOCS, p35S-polyketide_synthase-tOCS, pCmYLCV-At4CL1-tATPase, and pCmYLCV-npgA-tATPase).

Plasmids for pyruvate orthophosphate dikinase intron-containing hairpin silencing (PDK) silencing experiments were created using PDK sequence from pHannibal (*43*). Each plasmid consists of target gene sequence under 35*S* promoter, followed by the PDK intron, antisense target gene sequence and agrobacterium tumefaciens octopine synthase gene terminator (ocs terminator) (tables S1 and S4). Targets for silencing were fragments of *PAL4* (GenBank: MK689226.1), *C4H* (AT2G30490.1), *CHI *(sequence ID Niben101Scf01916g00004.1), *CHIL* (sequence ID Niben101Scf05989g01008.1), *CHIL3* (sequence ID Niben101Scf23113g00012.1), *F3H* (AT3G51240.1), cytochrome P450 superfamily protein (*P450*, AT5G05690.1). Plasmids for perturbing phenypropanoid metabolism encoded AAE13 (malonyl-CoA synthetase from *Arabidopsis thaliana*) (*44*), AtC4H (C4H from *A. thaliana*), GmCHIL (CHIL from *Glycine max*) (*45*), and MdCHIL (CHIL from *Malus domestica*) (*45*). Each plasmid encoded the target gene under control of 35*S* promoter and ocs terminator (table S4).

For transient expression in mammalian cells, *nnLuz*, *nnH3H*, *nnHispS*, *NpgA*, *PKS*, and *4CL* were expressed under the control of cytomegalovirus promoter and SV40 terminator. This set of plasmids was also created following the MoClo standard of the Golden Gate (*42*).

BeYDV and TMV viral vectors used in this work were assembled using GoldenBraid (*46*). They all were cloned under the regulation of the constitutive 35*S* promoter in a binary vector for *Agrobacterium tumefaciens*–based transient expression in *N. benthamiana* leaves.

### Yeast cell cultivation and transformation

*P. pastoris* GS115 cells were cultured using yeast extract peptone dextrose (YPD) medium with corresponding antibiotic or regeneration dextrose base (RDB) for His^+^ selection at temperature of 30°C (*47*).

For drop tests, we prepared yeast strains expressing* nnLuz, nnH3H, Pv4CL1* (GeneBank EU491511) or *At4CL1* (GeneBank At1g51680) or *NpgA*, and a polyketide synthase. Yeast competent cells already expressing nnLuz, nnH3H, Pv4CL1/At4CL1/NpgA were transformed by electroporation with 3 μg of AvrII-linearized plasmid encoding polyketide synthase to integrate the cassette into the GAP genomic locus. Yeast transformants were grown on RDB medium containing zeocin, G418, or hygromycin.

For LC-MS analysis, we created additional yeast strains coexpressing solely the polyketide synthase and At4CL1 (in case of plant synthases) or NpgA (in case of nnHispS). To confirm genomic integration, we extracted genomic DNA (ExtractDNA Blood kit, Evrogen, Russia) and carried out real-time polymerase chain reaction with primers for the bioluminescence-related and housekeeping genes (table S3).

### Drop test

To compare performance of strains, we resuspended yeast colonies in 40 μl of 1 M sorbitol. Ten microliters of aliquots of the suspension were dropped on YPD agar plates containing different concentrations of caffeic acid and zeocin in three technical replicas. The plates were then incubated for 15 min at room temperature until the drops dried completely and further incubated for 24 hours at 30°C.

We imaged a “no-substrate” frame on Fusion-Pulse.7 (Vilber Lourmat) with 7-min (in the case of 10 μM caffeic acid) or 1-min exposure (in the case of 100 μM caffeic acid). We then applied 10 μl of caffeic acid [220 mM in 40% dimethyl sulfoxide (DMSO) in phosphate-buffered saline (PBS)] to yeast spots and imaged a time-lapse series on Fusion-Pulse.7 (Vilber Lourmat) every 5 min with 0.5-s exposure. Imaging continued for 6 to 8 hours at room temperature.

We analyzed images using the Fiji ImageJ distribution (version 1.53t) (*48*) and custom Python scripts (Python version 3.8). For luminescence quantification, mean values in the region of interest after background subtraction were used. Background subtraction was performed using the following formula: signal = signal_raw_ − (background_mean_ − 3 * background_std_).

### Comparison of bioluminescence pathway with other luciferases in yeast

Yeast lines expressing NanoLuc or firefly luciferase (FFluc) have been created by transforming the wild-type strain with the corresponding plasmids (see table S4) and selecting on zeocin. For light emission experiments, yeast biomass was resuspended in PBS and used for a drop test in 96-well plates with YPD agar. After 30 hours of incubation at 30°C, we performed luminescence assays by applying 30 μl of 100 μM caffeic acid solution in PBS (for autoluminescence systems), 100 μM d-luciferin (LUCK-100, GoldBio) solution in PBS (for firefly luciferase), or Nano-Glo Live Cell substrate (N2011, Promega) solution (for NanoLuc). Plates were imaged in Tecan Spark with an open filter and automatic attenuation at 0.1-s exposure times for 60 min. Data processing was performed using custom Python scripts. Integral signal was quantified by integration along the “time” axis using the composite trapezoidal rule (trapz function from numpy Python package, v1.22.4).

### LC-MS experiments

#### Chemicals

The analytical standards of caffeic acid were purchased from Sigma-Aldrich (≥98.0), and hispidin was chemically synthesized and tested for purity in house (>95.0). Standard solution of two components was prepared in a 20% acetonitrile-water mixture acidified to 0.1% of acetic acid. High-performance liquid chromatography (HPLC)–grade acetonitrile was purchased from J.T.Baker. Deionized water was obtained from a Milli-Q System (USA), and acetic acid was purchased from Sigma-Aldrich (≥98.0).

#### Yeast sample preparation

Yeast colonies were grown in YPD agar medium with/without 10 mM caffeic acid overnight at 30°C. After 48 hours, biomass from all samples was moved into Eppendorf tubes and washed three times in milli-Q. About 100 mg of glass beads and 1000 μl of cold 70% methanol-water mixture were added to yeast pellets in every tube. The samples were treated by bead mill homogenizer at 13,000 rpm for 10 min and then centrifuged for 10 min at 13,000 rpm. A total of 700 μl of supernatant was transferred to another tube and lyophilized in the miVac machine. Before analysis, dry pellets were reconstituted by vortexing in 100 μl of 20% acetonitrile-water mixture, then acidified to 1% of acetic acid, and centrifuged at 13,000 rpm to remove insoluble debris. The supernatants were transferred to HPLC vials and analyzed.

#### Plant samples from transient transformation preparation

*N. benthamiana* leaves were transformed by infiltration with a mixture of agrobacterium strains encoding various combinations of individual transcription units (PpASCL, nnHispS, npgA, enhanced green fluorescent protein, and nnH3H). Forty-eight hours after infiltration, leaves were homogenized in liquid nitrogen and lyophilized. Extracts were obtained from 50 mg of dry plant biomass using 2 ml of 70% methanol, filtered through 0.45-μm glass fiber/polyvinylidene difluoride (Phenex) filter, and lyophilized in miVac machine. Dry residues were reconstituted in 200 μl of 70% methanol by vortexing and transferred to HPLC vials for analysis.

#### LC-MS analysis of yeast samples

LC-MS analysis was carried out on an Ultimate 3000 RSLC nano HPLC system connected to an Orbitrap Fusion Lumos mass spectrometer (Thermo Fisher Scientific) with the loading pump used as an analytical flow gradient pump. Samples were separated on Agilent Eclipse Plus C8 3.5-μm column 2.1 × 150 mm at a flow rate of 200 μl/min. Separation was performed by a linear gradient of 90% acetonitrile, 0.1% formic acid, and 10 mM ammonium formate (buffer B) in 99.9% H_2_O and 0.1% formic acid (buffer A) from 3 to 95% B in 10 min. MS data were collected in negative ion mode for full MS scans at 30,000 resolution, two microscans per spectra, 3 × 10^6^ automatic gain control, and 200-ms accumulation time. The spectra were collected in profile type. Identification of components was carried out by exact mass of the ions and coincidence of retention time of the components and its analytical standards. Raw data were collected and processed on Thermo Xcalibur Qual and Skyline software. The MS peaks were extracted at a mass tolerance of 5 parts per million. For compound quantification, the corresponding peak area for each sample was used. Analysis was performed using custom Python scripts.

#### LC-MS analysis of plant samples

LC-MS analysis was carried out on an Ultimate 3000 RSLC nano HPLC system connected to a QExactive Plus mass spectrometer (Thermo Fisher Scientific, USA). A Gemini C18 3-m NX LC column 100*2.1 mm (Phenomenex) at a flow rate of 200 liters/min. Separation was done by a linear gradient of 90% acetonitrile in water, 10 mM ammonium formate, and 0.1% formic acid (buffer B) in 99.9% H_2_O, 10 mM ammonium formate, and 0.1% formic acid (buffer A): 1% B at 0 min, 50% B at 3 min, and 99% B at 8 min, followed by 3-min wash at 99% B and 2-min equilibration at 1% B before the next run. Ultraviolet data were collected at 220 nm. MS1 and MS2 spectra were recorded at 30,000 and 15,000 resolution respectively with higher-energy collisional dissociation fragmentation.

### Plant material and growth conditions

In vitro plants cultivated using Murashige and Skoog basal medium supplemented with sucrose (30 g/liter), indolyl acetic acid (0.2 mg/liter), and agar (8 g/liter) (pH 5.7). The leaves of 3-week-old in vitro plants of *N. benthamiana* were used for stable transformation. In vitro cultures were grown at 25°C under a long-day condition (16-hour light/8-hour dark). For Agrobacterium infiltration, we used 4- to 6-week-old plants grown in soil under a neutral day lighting regime (12-hour light/12-hour dark).

### Agrobacterium-mediated transformation

We used *A. tumefaciens* strain AGL0 to generate transgenic plants. AGL0 transformed with a binary vector was cultured on a shaker overnight at 28°C in LB medium supplemented with spectinomycin (300 μg/ml) and rifampicin. Bacterial cultures were diluted in liquid Murashige and Skoog medium to optical density at 600 nm (OD_600_) = 0.6.The dissected *N. benthamiana* leaves were incubated with bacterial culture for 30 min. Explants were blotted dry on sterile filter paper and placed onto filter paper overlaid on solid Murashige and Skoog medium with sucrose (30 g/liter), agar (8 g/liter), 6-benzylaminopurine (1 mg/liter), and indolyl acetic acid (0.1 mg/liter). After 2 days of cocultivation at 25°C, the explants were transferred to the same Murashige and Skoog medium supplemented with cefotaxime (500 mg/liter) and kanamycin (75 mg/liter). After 3-week incubation in the dark, shoots that regenerated were cut and grown on the rooting medium [Murashige and Skoog salt and vitamins, sucrose (30 g/liter), agar (8 g/liter), indole-3-butyric acid (0.3 mg/liter), and kanamycin (75 mg/liter)].

### Agroinfiltration of *N. benthamiana*

Twenty milliliters of overnight culture of bacteria was collected by centrifugation (3500*g* for 20 min) and resuspended in 10 ml of MMA buffer [10 mM MES (pH 5.7), 10 mM MgCl_2_, and 200 μM acetosyringone]. Cultures were incubated for 3 hours at 28°C and 600 rpm and then diluted to OD_600_ = 0.6. Bacteria were gently delivered into the abaxial leaf surface using 1-ml syringe without needle. To suppress gene silencing, we coinfiltrated agrobacteria encoding P19 protein (*49*) as 25% of bacterial suspension. The luminescence was checked 2 to 4 days after infiltration in Fusion-Pulse.7 (Vilber Lourmat).

Image analysis was performed using the Fiji distribution of ImageJ and custom Python scripts. For luminescence quantification, mean values in the region of interest were normalized by the mean value of leaf luminescence. Background subtraction was performed using the following formula: signal = signal_raw_ − background_mean_.

For BeYDV and TMV agroinfiltration, the OD_600_ was adjusted to 0.1. In the case of BeYDV expression, the silencing suppressor P19 and the replication-related proteins Rep/RepA were diluted at a ratio of 1:3, while they were not included for TMV. Samples were collected on day 2 after infiltration and transferred to a white 96-well microplate filled with liquid 1/2 Murashige and Skoog medium. Luminescence measurements were taken until day 5 for BeYDV and until day 7 for TMV using the GloMax-Multi Detection System (Promega). For capturing time-course pictures of TMV, a Sony ILCE 7S digital camera was used with the following settings: exposure time of 30 s, ISO of 20,000, and an aperture of 2.8.

### BY-2 cultivation and transient transformation

*Nicotiana tabacum* BY-2 cell suspension was provided by J. Petrasek group. The culture was grown in Murashige and Skoog with 2,4-D (0.2 mg/liter), KH_2_PO_4_ (200 mg/liter), thiamine (1 mg/liter), myo-inositol (100 mg/liter), and sucrose medium (30 g/liter) supplemented with sucrose (30 g/liter) on a rotary shaker at 130 rpm under the dark condition at 27°C.

For transient gene expression, we used a 7-day-old culture. The protocol was adapted from (*50*). Cell suspension was aliquoted into wells of 96-well plates with the round perforated bottom. Overnight culture of Agrobacterium was resuspended in infiltration buffer [Murashige and Skoog medium, 200 μM acetosyringone, 15 mM MES (pH 5.6), sucrose (50 g/liter), and glucose (2 g/liter)], and OD_600_ was adjusted to 0.5 (to 0.2 in the case of P19). Agrobacterium cell suspensions were added to each cell bunch and incubated for 1 to 2 hours at 25°C in the dark. After removing agrobacteria by centrifugation, plates were incubated in the dark for 3 days at room temperature.

BY-2 plate analysis was performed 72 hours after infiltration using the luminescence module of microplate reader Tecan Spark, exposure time of 1 s measured with open filter. Data processing was performed using custom Python scripts (Python version 3.8). For luminescence quantification, values in the plate wells were used.

### Comparison to other luciferases in BY-2 cells

Transformations of BY-2 cells were made by agrobacterial strains encoding plasmids. Fifty hours after infiltration, BY-2 cells were supplemented with 150 μl of Murashige and Skoog medium (M5524, Sigma-Aldrich; pH 5.7), containing 100 μM d-Ln (LUCK-100, GoldBio) in the case of FFLuc and Nano-Glo Live Cell substrate (kit N2011, Promega) in case of NanoLuc or no substrate in the case of autoluminescent systems. Plates were imaged in Tecan Spark imager with an open filter and automatic attenuation at 0.1-s exposure times for 60 min. Data processing was performed using custom Python scripts. Integral signal was quantified by integration along the time axis using the composite trapezoidal rule (trapz function from numpy Python package, v1.22.4).

### Solutions for yeast and plant experiments

For yeast experiments, we prepared 220 mM stock solutions of caffeic and coumaric acids (Sigma-Aldrich; purity ≥98.0%) in PBS with 40% DMSO. For plant experiments, 220 mM stock solutions were prepared in the MMA buffer with 40% DMSO. Less concentrated solutions (1, 0.01, and 0.0001 mM) were prepared by diluting the stocks in the MMA buffer. Stock solutions of 4 mM hispidin and 2 mM luciferin in DMSO were prepared just before each experiment and further dissolved to the final concentration in the MMA buffer.

MG132 (Abcam) (*51*) was dissolved in ethanol to 10 mM stock solution. A total of 1 and 40 μM experimental solutions were prepared in the MMA buffer.

### Mammalian cell culture

HEK293NT cells were cultured in Dulbecco’s modified Eagle’s medium (DMEM) complete medium at 37°C with 5% CO_2_. For transfections, cells were seeded into 24-well plates with 500 μl of DMEM cell culture medium per well and transfected with a combination of four plasmids in 2 μl of PolyFect reagent (QIAGEN). The mixture of plasmids for transfection contained 150 ng of each plasmid encoding luciferase (nnLuz), hispidin-3-hydroxylase (nnH3H), 4-coumaroyl-CoA ligase (At4CL1), and plant type III polyketide synthase (PKS). The control cell samples were transfected with 150 ng of plasmids encoding nnLuz and nnH3H, 110 ng of plasmid encoding NpgA (molar equivalent to 150 ng of plasmid encoding At4CL1), and 280 ng of plasmid encoding nnHispS (molar equivalent to 150 ng of plasmid encoding PKS). Comparison of the bioluminescence signal from cells with different PKS was performed 24 hours after transfection on IVIS Spectrum (PerkinElmer) with open-filter bioluminescence detection, an exposure time of 1 min, a binning of 8, an F/stop of 2. Bioluminescence was measured for 20 min after changing the medium to 150 μl of Dulbecco’s PBS per well and adding 22.5 μl of 2.5 mM caffeic acid solutions to reach 330 μM concentration in the well.

Image analysis was performed using Living Image 4.5.5 software and custom Python scripts. For luminescence quantification, we used total flux (in photons per second) normalized by the mean value of the control sample (expressing nnHispS). Background subtraction was performed using the following formula: signal = signal_raw_ − background_mean_.

### Data presentation and statistics

Data are plotted as box-and-whisker plots implemented in Seaborn (https://seaborn.pydata.org/) package (version 0.12, Python version 3.8). Unless noted otherwise in figure captions, the boxes on the graphs extend from the lower to upper quartile values of the data, the horizontal line represents the median, and whiskers represent the full data range. Gray or colored dots represent individual values. A pair-wise post hoc two-sided Mann-Whitney *U* tests (scikit-posthocs package, https://pypi.org/project/scikit-posthocs/, version 0.7.0) with *P* values corrected by the step-down method using Sidak adjustments were computed (fig. S29). Kruskal-Wallis *H* tests (scipy.stats package, www.scipy.org/, SciPy version 1.9.2), followed by multiple pairwise post hoc Conover’s tests (scikit-posthocs package) with *P* values corrected by the step-down method using Sidak adjustments were computed (figs. S5, S6, S9, S10, S11, S14, S15, S19, S20, S22, S23, S24, S25, S26, S27, S28, S31, S32, and S33). Sample numbers (*N*) are reported in the figure legend. Some data (figs. S12 and S13) are plotted as dot plots with Spearman ranked correlation coefficient (pandas package, version 1.5.3).
