# Supplementary material for: A hybrid pathway for self-sustained luminescence
Source: Sci Adv. 2024 Mar 8;10(10):eadk1992. doi: 10.1126/sciadv.adk1992 (PMC10923510; doi:10.1126/sciadv.adk1992)
Supplement: Supplementary file 1 — Figs. S1 to S33 Tables S1 to S4 [file sciadv.adk1992_sm.pdf]

Science Advances
AAAS

Supplementary Materials for

A hybrid pathway for self-sustained luminescence

Kseniia A. Palkina *et al.*

Corresponding authors: Alexander S. Mishin, alexander@planta.bio; Karen S. Sarkisyan, karen@light.bio

Sci. Adv. **10**, eadk1992 (2024)
DOI: 10.1126/sciadv.adk1992

This PDF file includes:

Figs. S1 to S33
Tables S1 to S4

Supplementary materials

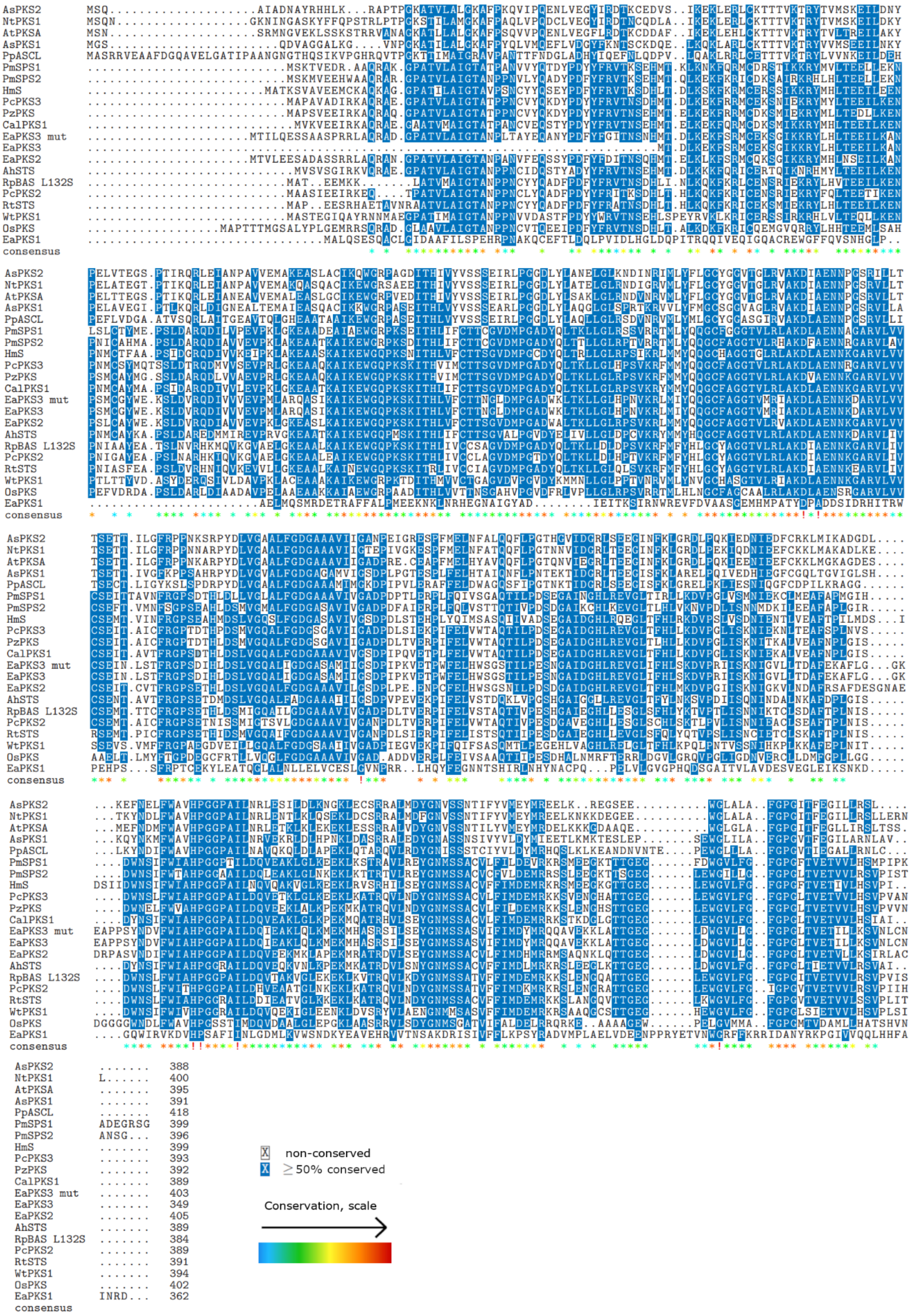

Fig. S1. Amino acid alignment of type III polyketide synthases of plant origin used in this work. Conservative amino acids are shown in blue. Conservation level is colour-coded in the consensus row.

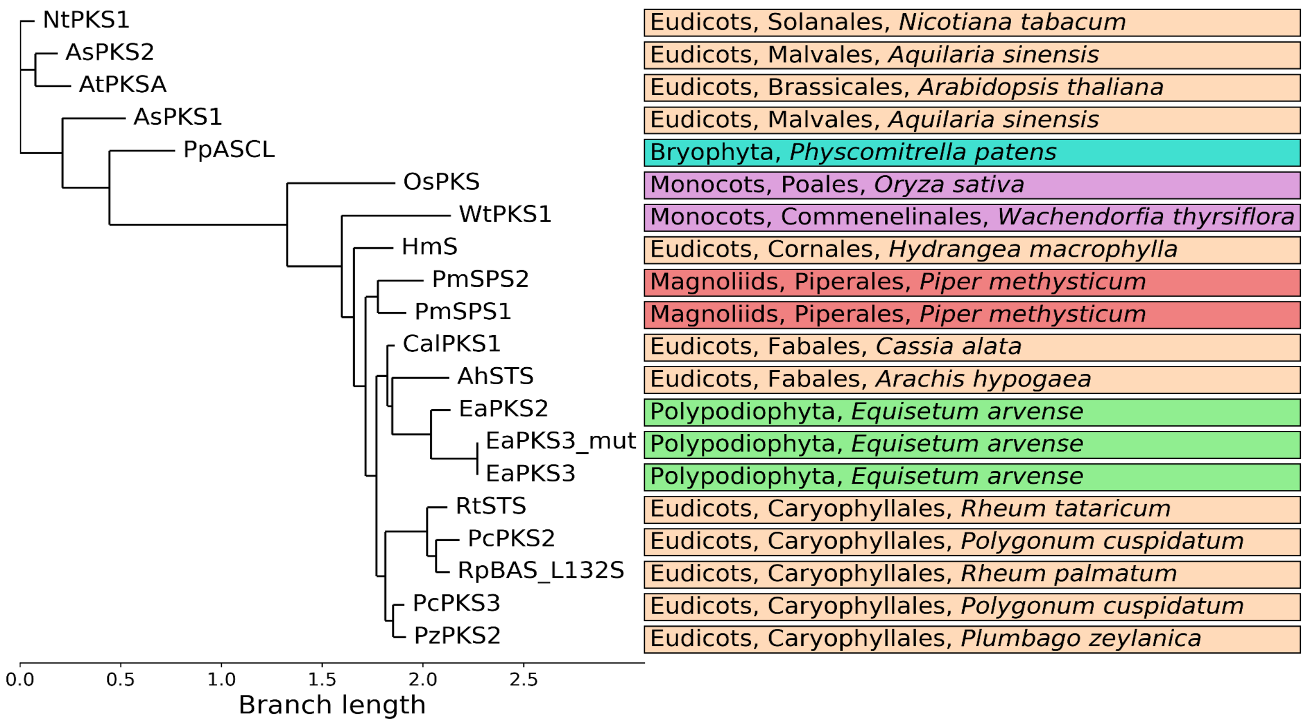

Fig. S2. Sequence similarity tree of type III polyketide synthases assessed in this work.
For each enzyme, order and species name are indicated in rectangles on the right, coloured by clade.

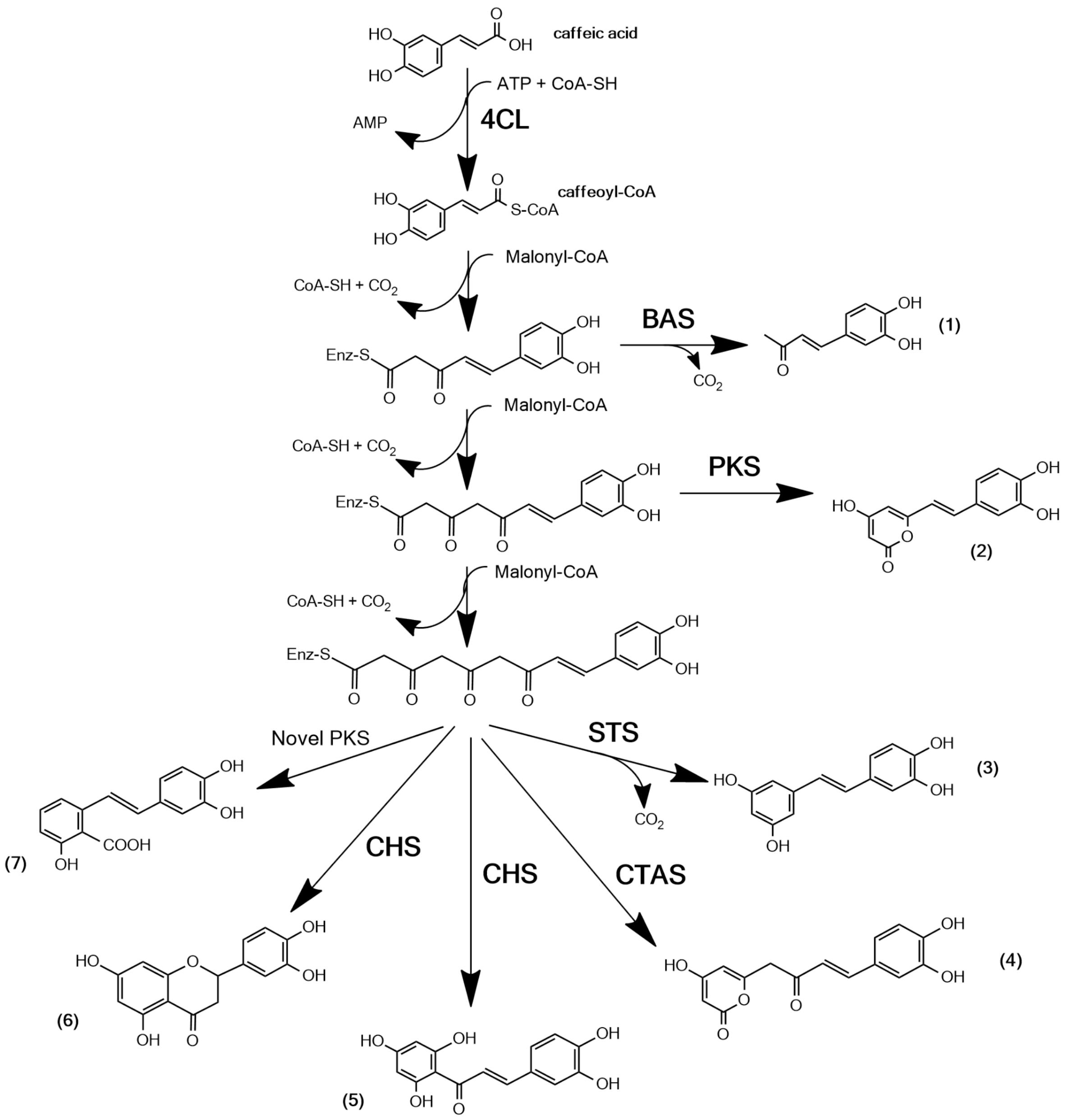

Fig. S3. Variety of caffeic acid polyketide derivatives. Hispidin biosynthesis and other plant metabolite formation catalysed by plant type III polyketide synthase and 4CL. (1) hydroxybenzalacetone, (2) hispidin, (3) hydroxyresveratrol, (4) hydroxycoumaroyltriacetic acid lactone, (5) hydroxynaringenin chalcone, (6) hydroxynaringenin, (7) hydroxehydrangenic acid.

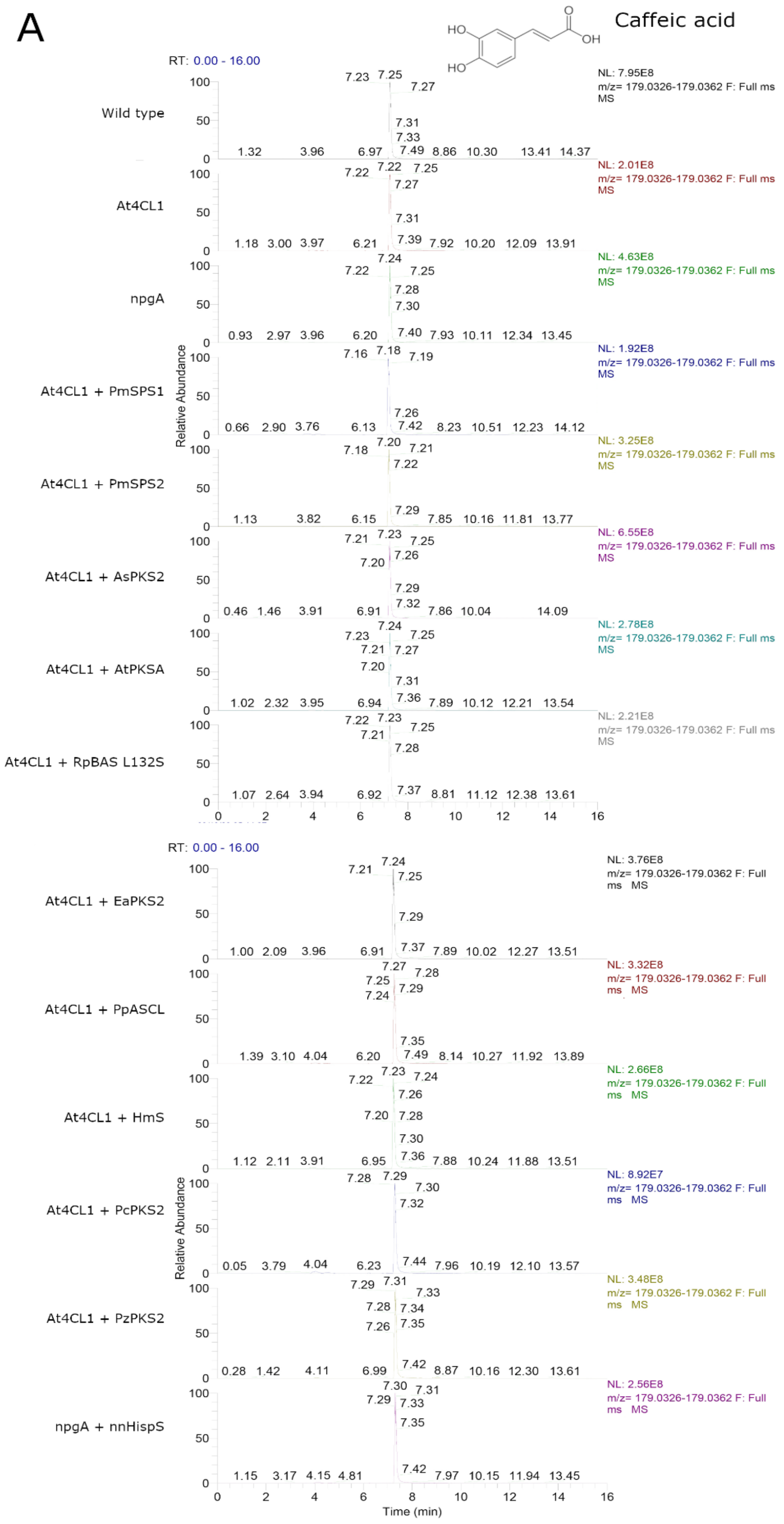

Fig. S4A. See the legend below.

B

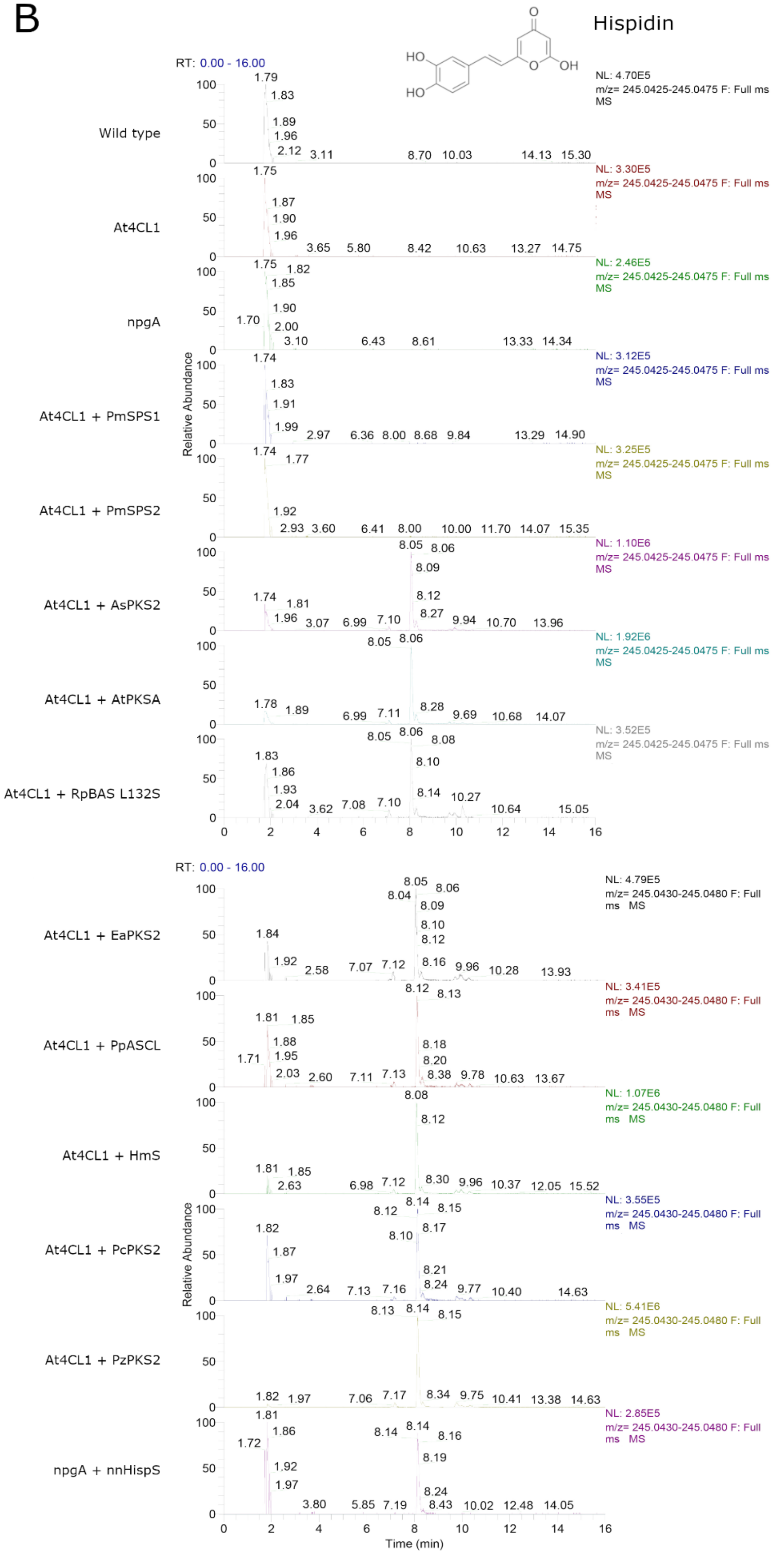
Hispidin
RT: 0.00 - 16.00
Wild type
NL: 4.70E5
m/z= 245.0425-245.0475 F: Full ms MS
At4CL1
NL: 3.30E5
npgA
NL: 2.46E5
At4CL1 + PmSPS1
NL: 3.12E5
At4CL1 + PmSPS2
NL: 3.25E5
At4CL1 + AsPKS2
NL: 1.10E6
At4CL1 + AtPKSA
NL: 1.92E6
At4CL1 + RpBAS L132S
NL: 3.52E5
Relative Abundance
RT: 0.00 - 16.00
At4CL1 + EaPKS2
NL: 4.79E5
m/z= 245.0430-245.0480 F: Full ms MS
At4CL1 + PpASCL
NL: 3.41E5
At4CL1 + HmS
NL: 1.07E6
At4CL1 + PcPKS2
NL: 3.55E5
At4CL1 + PzPKS2
NL: 5.41E6
npgA + nnHispS
NL: 2.85E5
Relative Abundance
Time (min)

Fig. S4B. See the legend below.

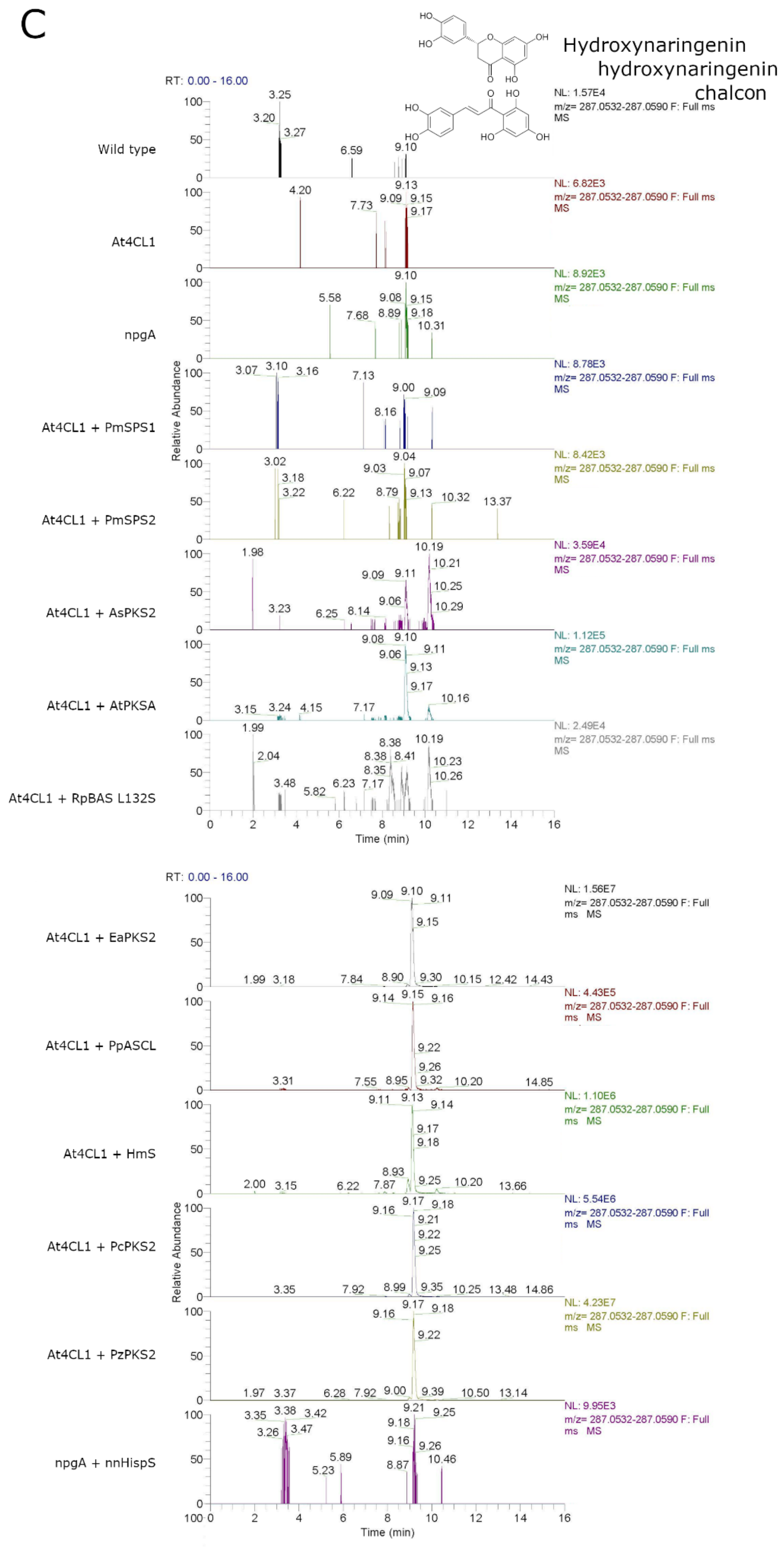

Fig. S4. LCMS spectra of yeast *Pichia pastoris* strains co-expressing polyketide synthase genes and At4CL1 (or NpgA in the case of nnHispS), and control strains. Each sample is represented by two spectra reflecting

the presence of added caffeic acid (**A**) and hispidin (**B**) and hydroxynaringenin or hydroxynaringenin chalcone (**C**) in the extract.

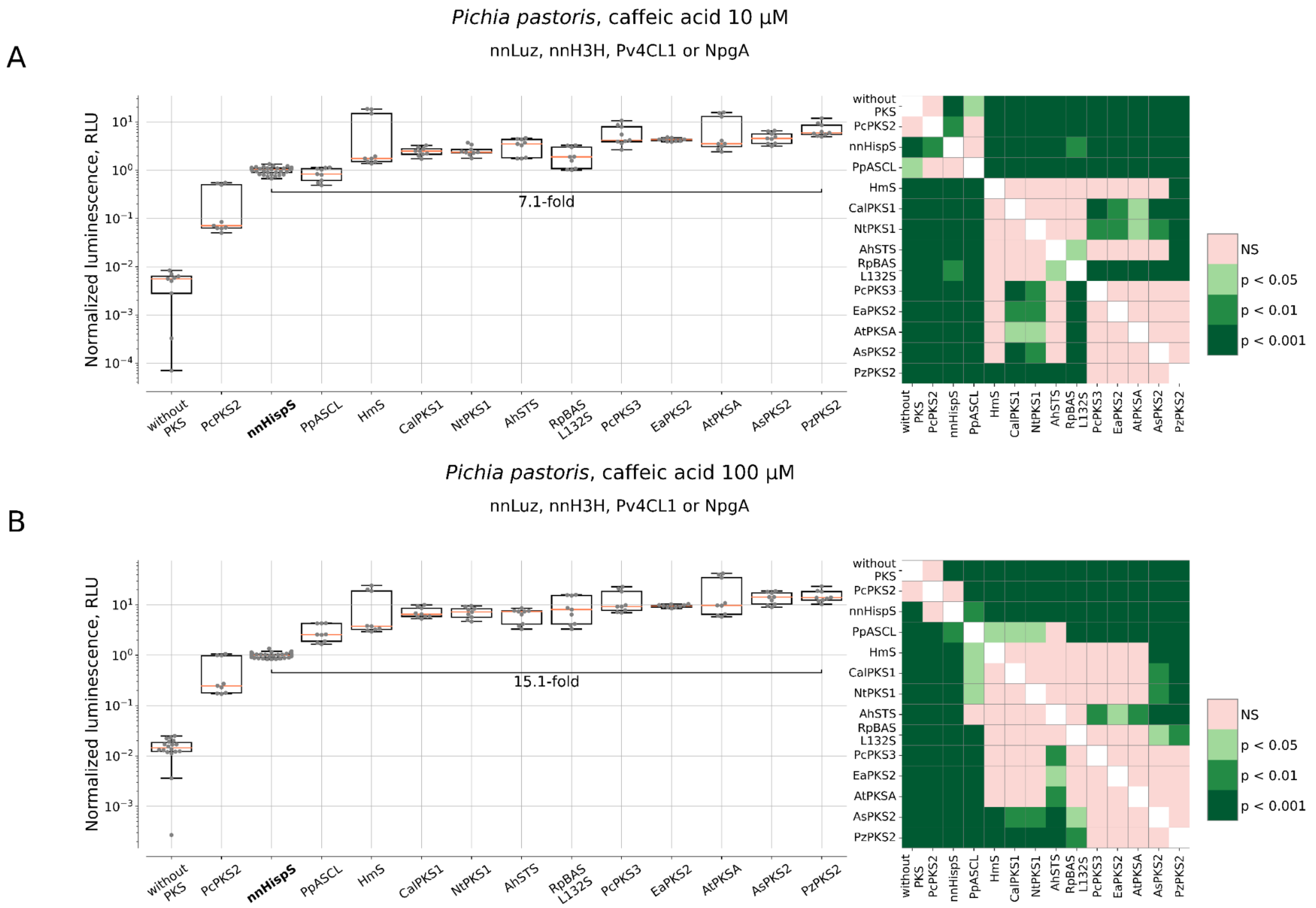

Fig. S5. Luminescence of polyketide-synthase-expressing yeast strains at two different concentrations of externally supplied caffeic acid: 10 μM (**A**) and 100 μM (**B**). Box-and-whiskers plots are accompanied by colour-coded p-values of Conover's test, NS — non-significant. Kruskal-Wallis H Test: H-statistic = 133.08, p = 5.1e-22 (A) or H-statistic = 155.89, p = 1.3e-26 (B). The difference between mean values are indicated below the brackets between the box plots. N = 9-34 colonies per box plot.

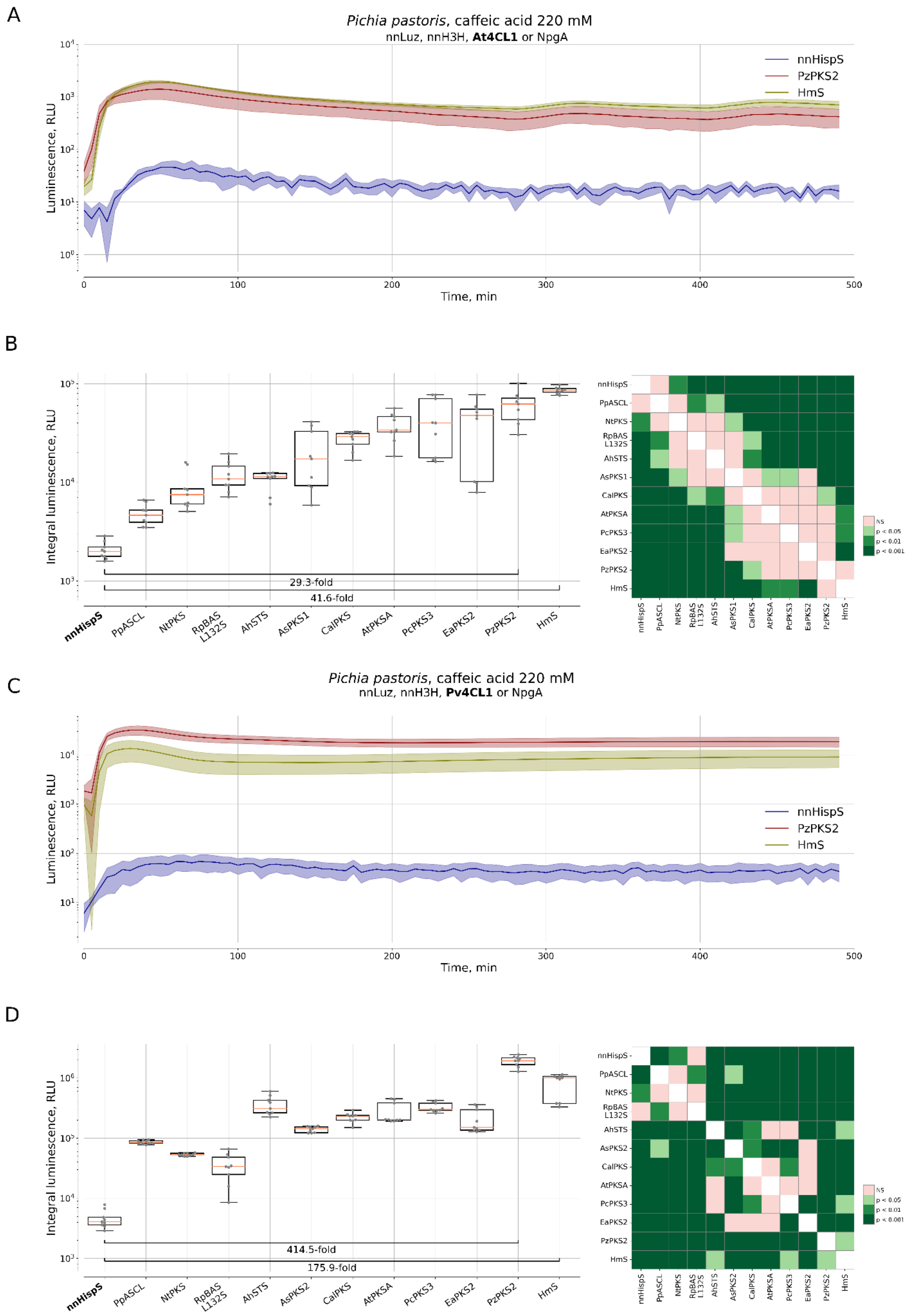

Fig. S6. Luminescence of polyketide-synthase-expressing yeast strains in a medium containing 220 mM caffeic acid. Two coumarate-CoA ligases were co-expressed with Type III polyketide synthases: At4CL1 (**A, B**) and Pv4CL1 (**C, D**). Panels **A** and **C** show kinetics of luminescence. Data are shown as mean ± SD. Panels **B** and **D** show integral luminescence for 495 min. Box-and-whiskers plots are accompanied by colour-coded p-values of Conover's test, NS — non-significant. Kruskal-Wallis H Test: H-statistic = 88.25, p = 3.7e-14 (B) or

H-statistic = 98.41, p = 3.3e-15 (D). The difference between mean values are indicated below the brackets between the box plots. N = 3-18 colonies per box plot.

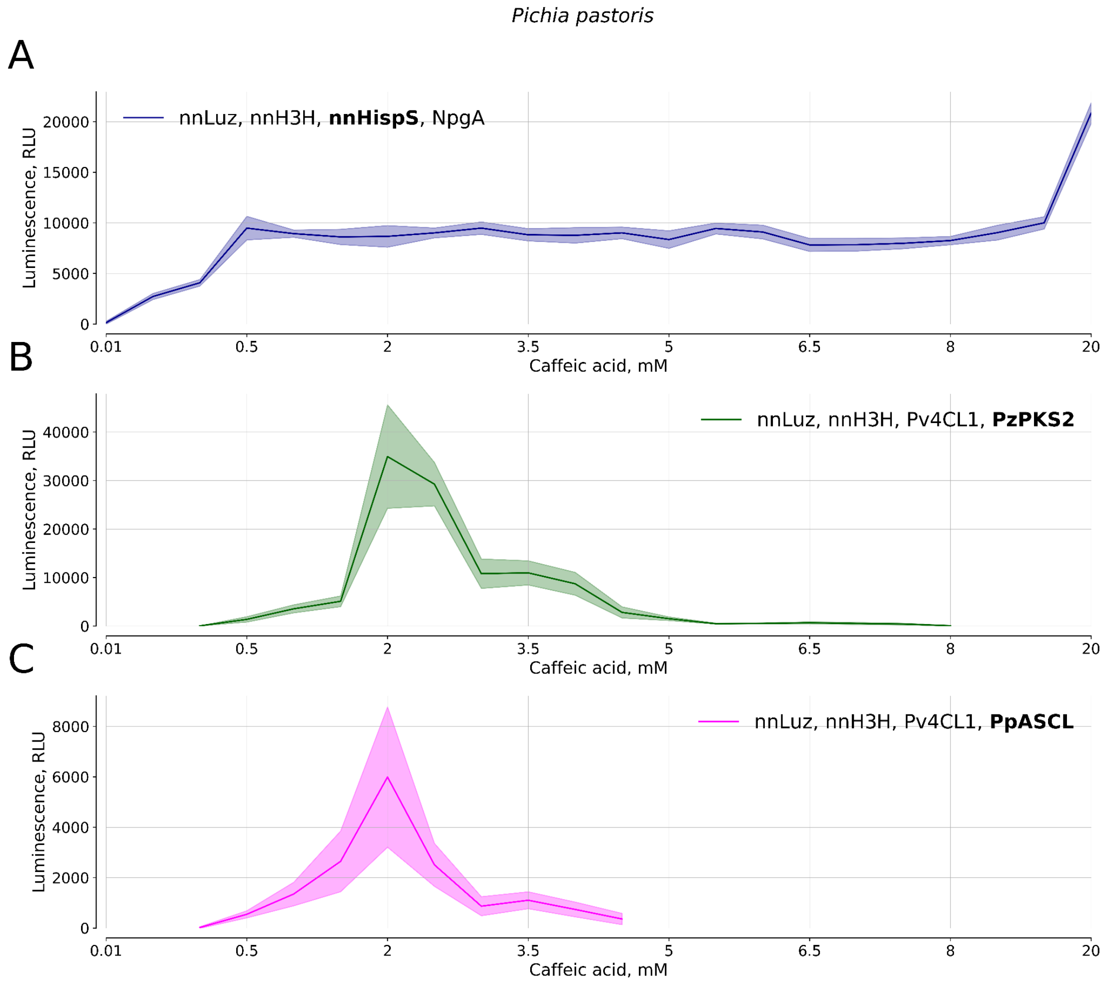

Fig. S7. Luminescence of PpASCL-, PzPKS2- and nnHispS-expressing yeast strains on agar plates comprising caffeic acid at various concentrations. In this experiment, yeast were pre-incubated on agar plates with caffeic acid for 24 hours before measurements.

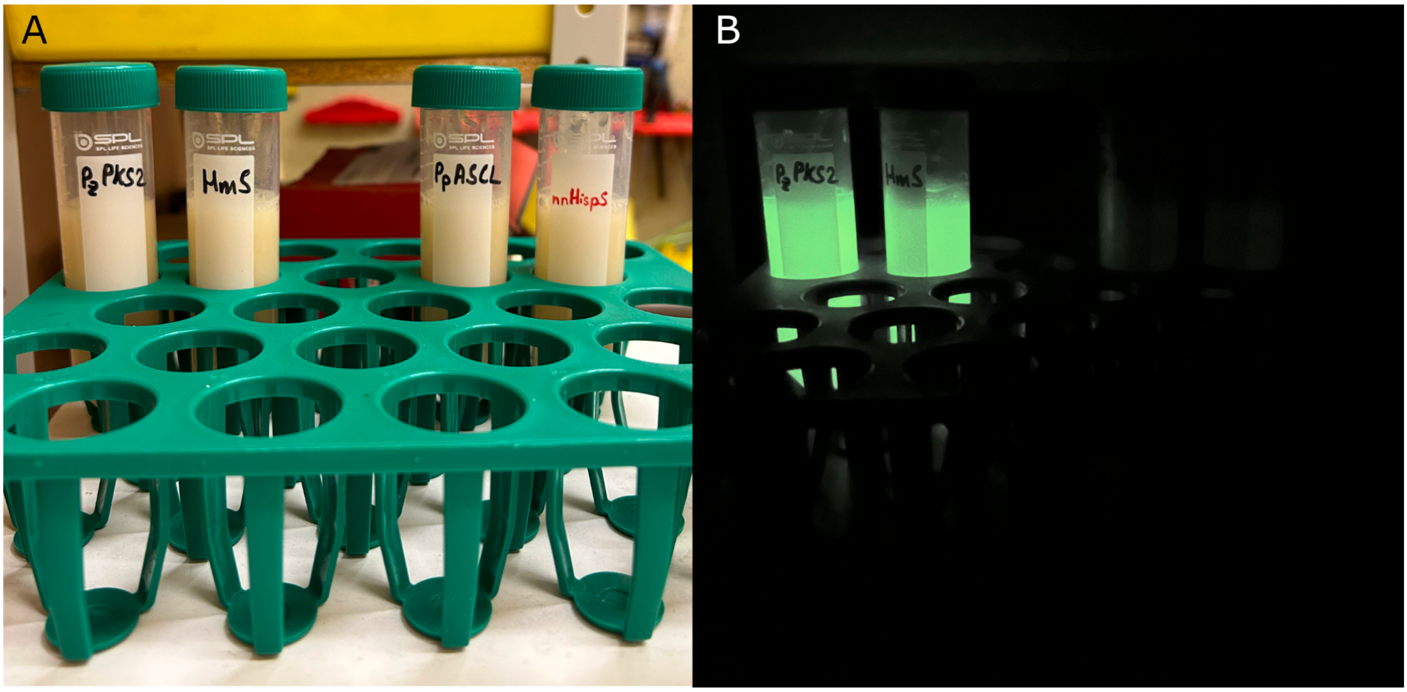

Fig. S8. A photo of yeast cultures expressing nnLuz, nnH3H, and (Pv4CL1 and PzPKS2), (Pv4CL1 and HmS), (Pv4CL1 and PpASCL) or (nnHispS and NpgA), in ambient light (A) and in the dark (B).
For the experiment, yeast were resuspended in a media containing 22 mM caffeic acid.
The photo in the dark was taken on iPhone 14 with exposure time of 0.5 s (26 mm lens, f1.5, ISO 12500).

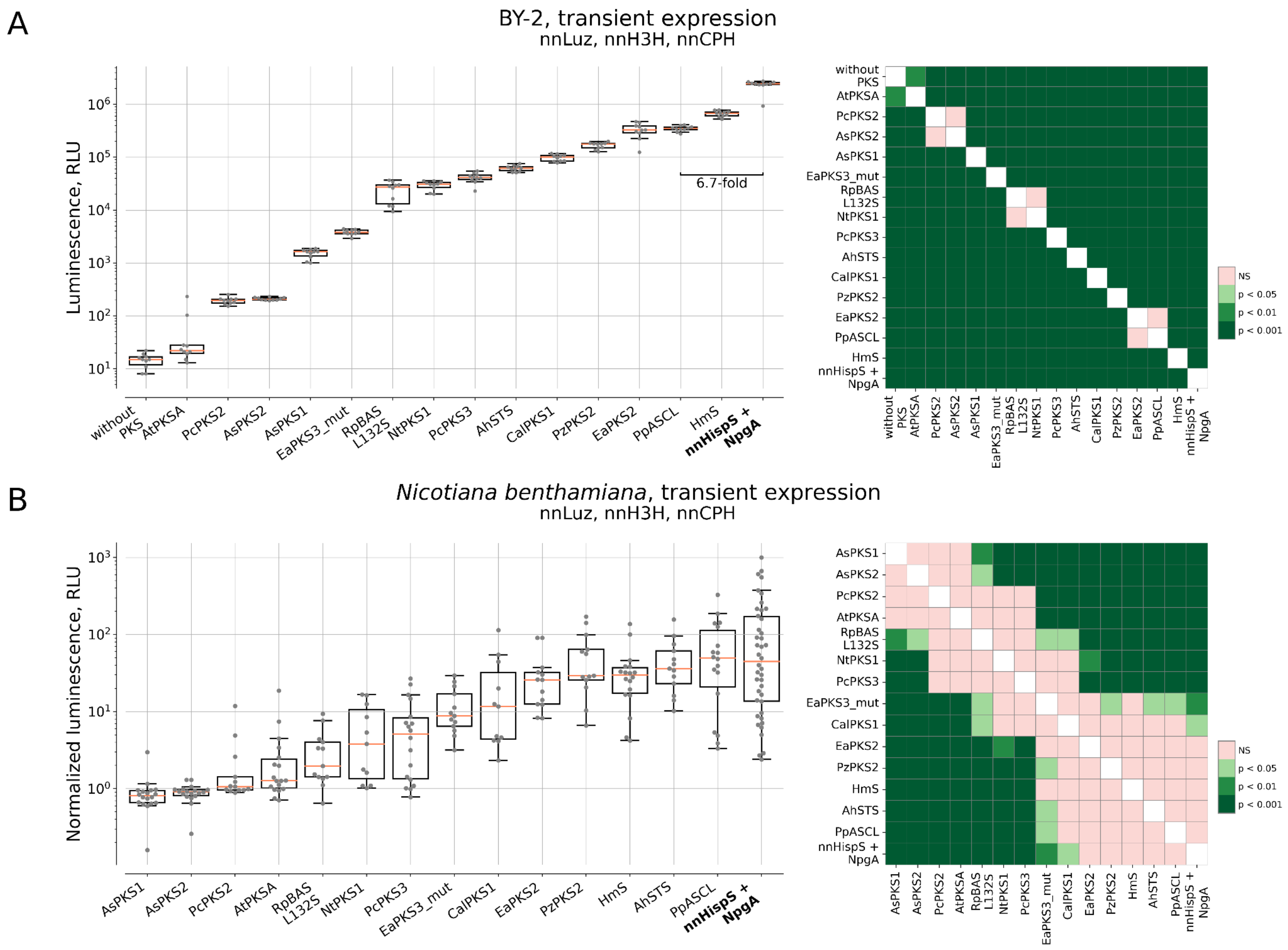

Fig. S9. Luminescence of BY-2 cells (A) or *Nicotiana benthamiana* leaves (B) expressing polyketide synthase, nnLuz, nnH3H, nnCPH and P19 (and NpgA in the case of nnHispS). N = 10 cell packs per box plot in the case of panel A; N = 11-42 leaves per box plot in the case of panel B. Box-and-whiskers plots are accompanied by colour-coded p-values of Conover's test, NS — non-significant. Kruskal-Wallis H Test: H-statistic = 157.0, p = 9.7e-26 (A) or H-statistic = 175.89, p = 4.4e-30 (B). The difference between mean values are indicated below the brackets between the box plots.

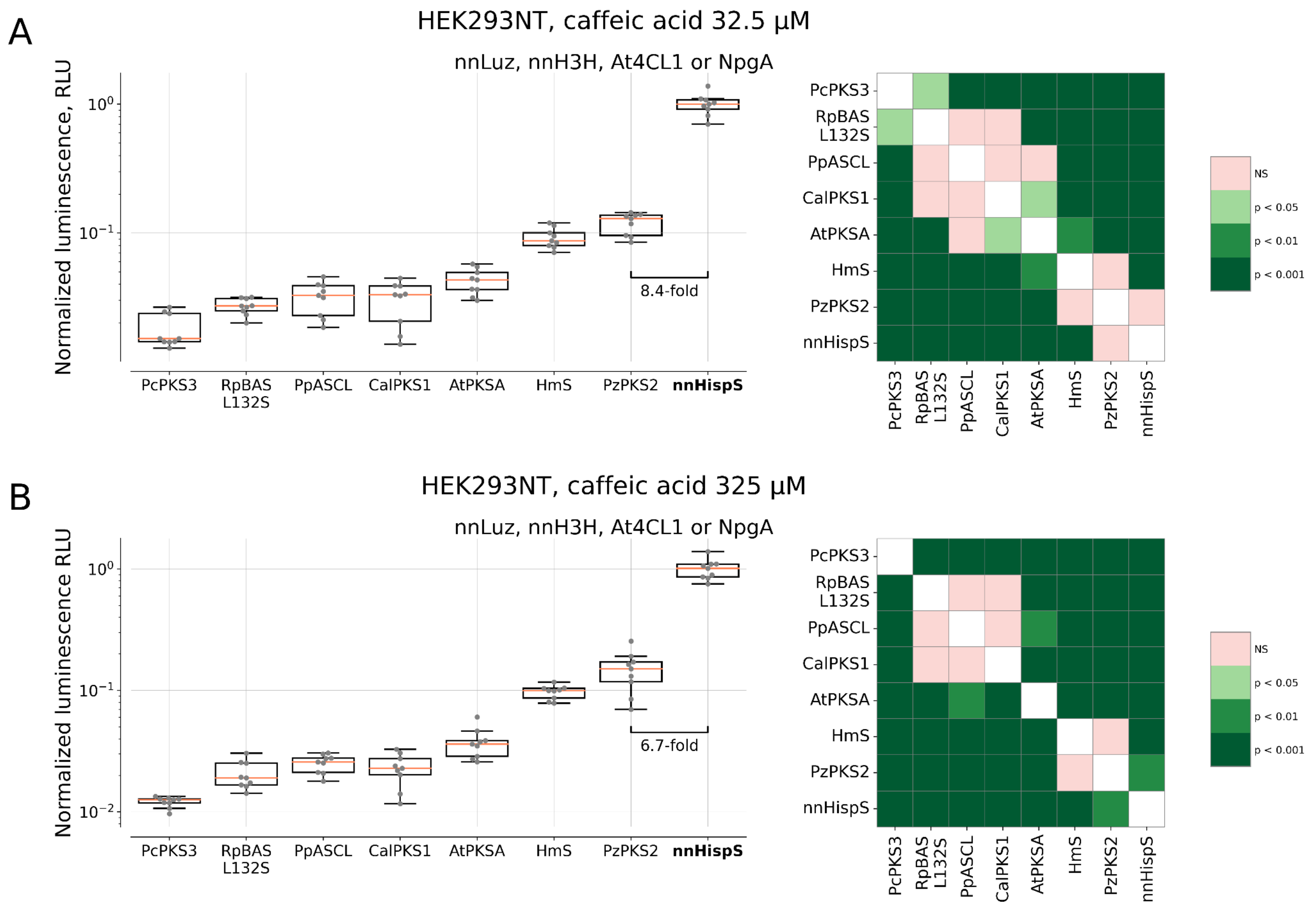

Fig. S10. Luminescence of polyketide-synthase-expressing human cells (HEK293NT) in a medium containing 32.5 μM (A) or 325 μM (B) caffeic acid. N = 9. Plant polyketide synthases were co-expressed with At4CL1, while nnHispS was co-expressed with NpgA. Box-and-whiskers plots are accompanied by colour-coded p-values of Conover's test, NS — non-significant. Kruskal-Wallis H Test: H-statistic = 557.74, p = 3.0e-116 (A) or H-statistic = 588.02, p = 9.3e-123 (B). The difference between mean values are indicated below the brackets between the box plots.

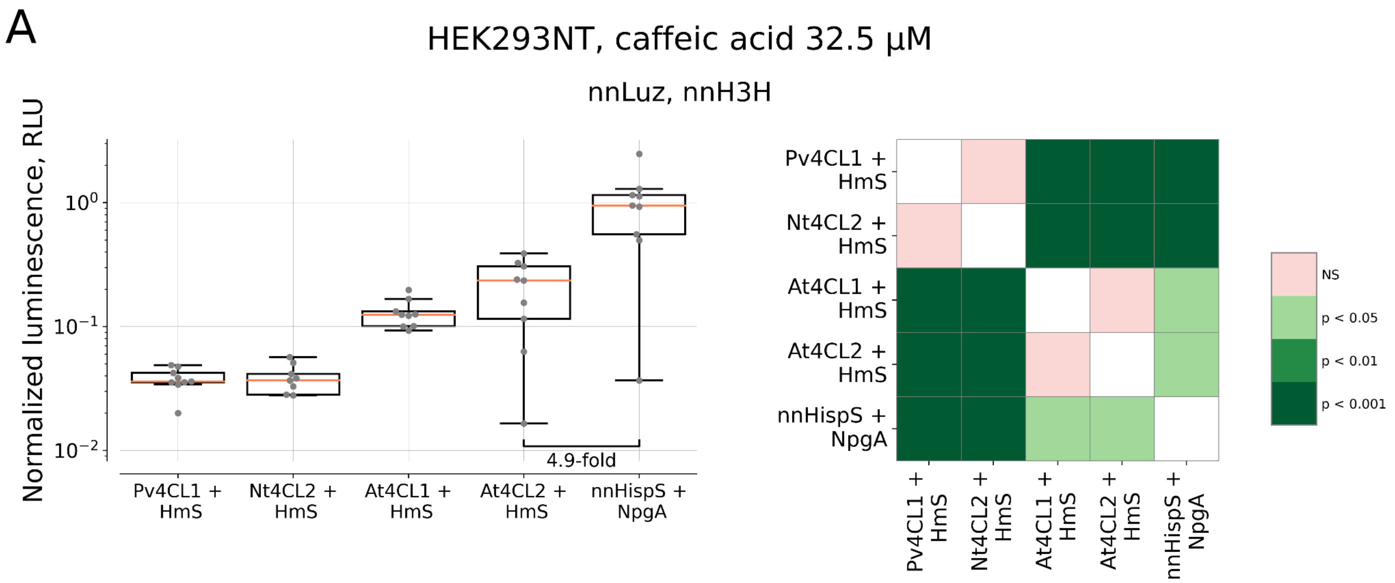

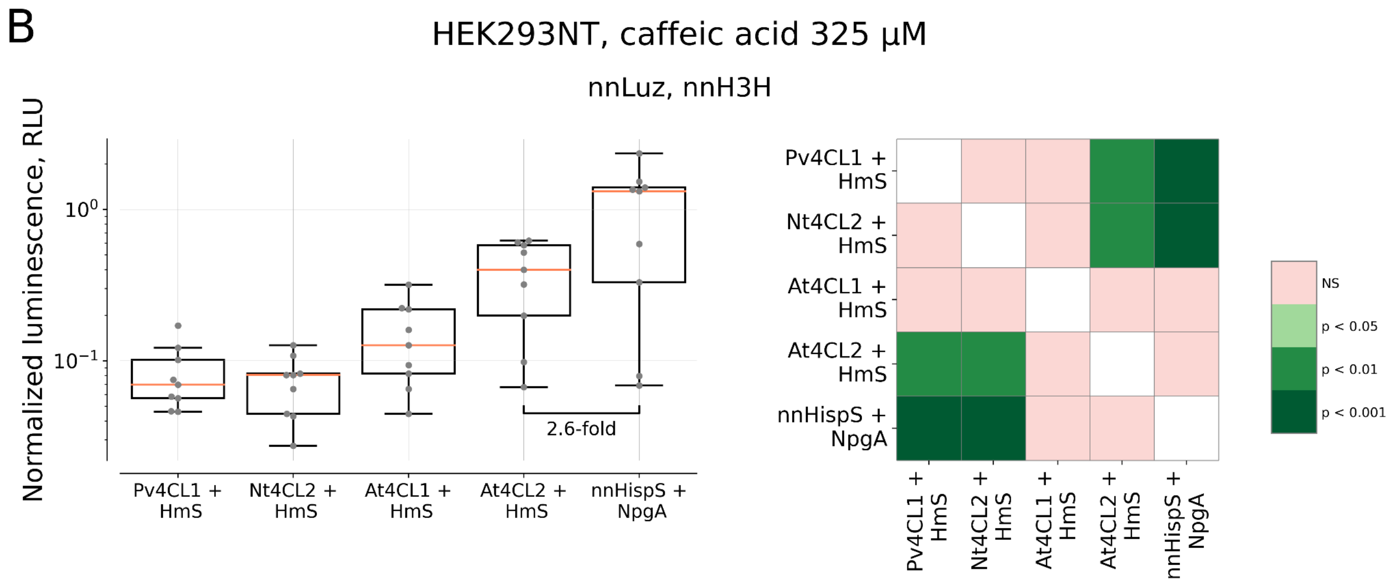

Fig. S11. Effects of 4-coumaroyl-CoA ligases on luminescence of mammalian cells HEK293NT expressing HmS and other genes required for luminescence (nnLuz, nnH3H), in a medium containing 32.5 μM (A) or 325 μM (B) caffeic acid. N = 9. Box-and-whiskers plots are accompanied by colour-coded p-values of Conover's test, NS — non-significant. Kruskal-Wallis H Test: H-statistic = 271.49, p = 1.5e-57 (A) or H-statistic = 188.04, p = 1.4e-39 (B). The difference between mean values are indicated below the brackets between the box plots.

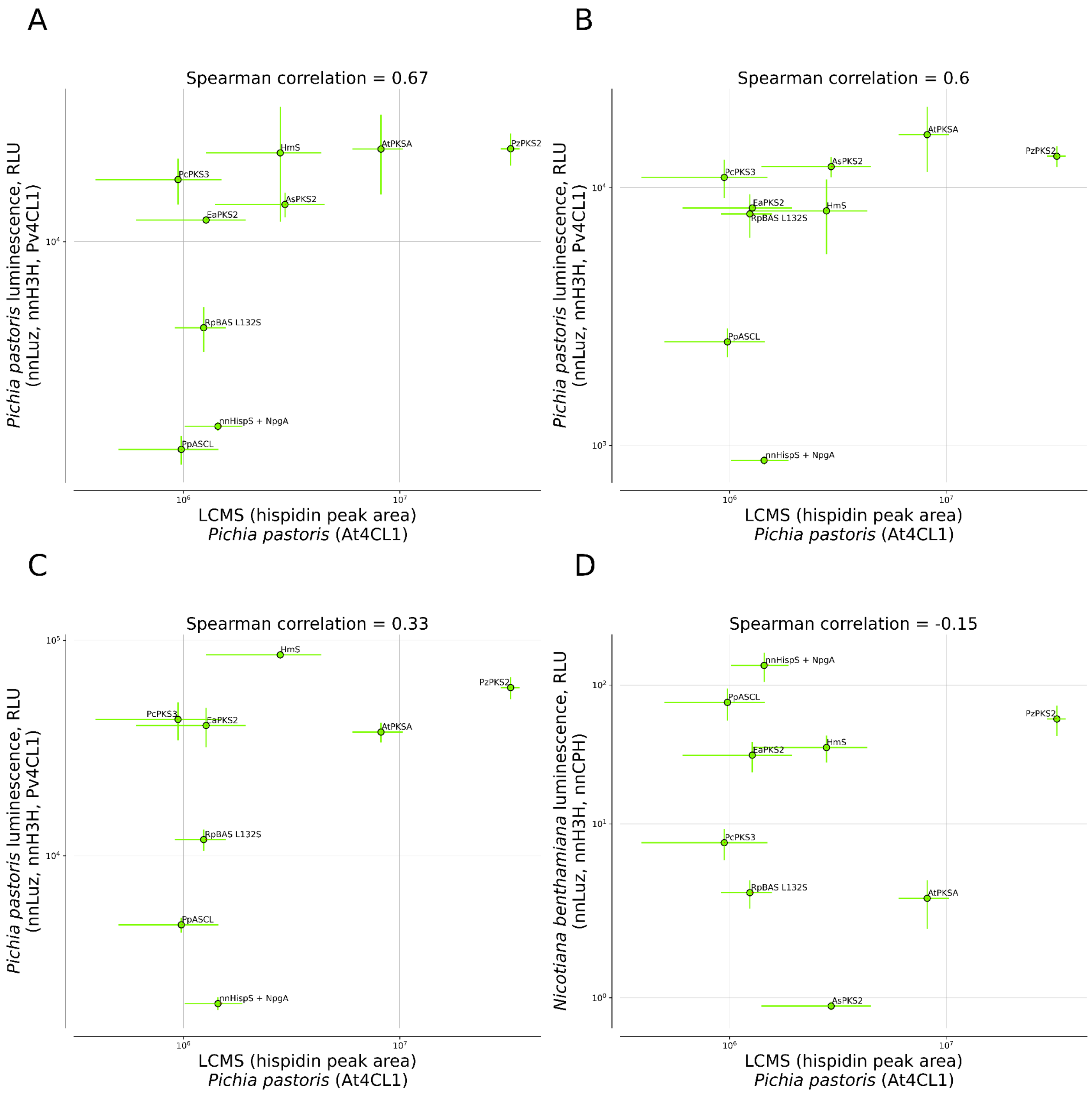

Fig. S12. Correlation between luminescence and hispidin peak area in LCMS data (N=3), for strains producing detectable amounts of hispidin. Panels **A**, **B** and **C** show data for yeast strains (N=9-18) grown on agar plates containing 10uM caffeic acid (**A**), 100uM caffeic acid (**B**), 200mM caffeic acid (**C**). Panel **D** shows data for transient expression in *N. benthamiana* leaves (N=13-42 leaves). The data are shown as mean (green dots) ± SE (green lines).

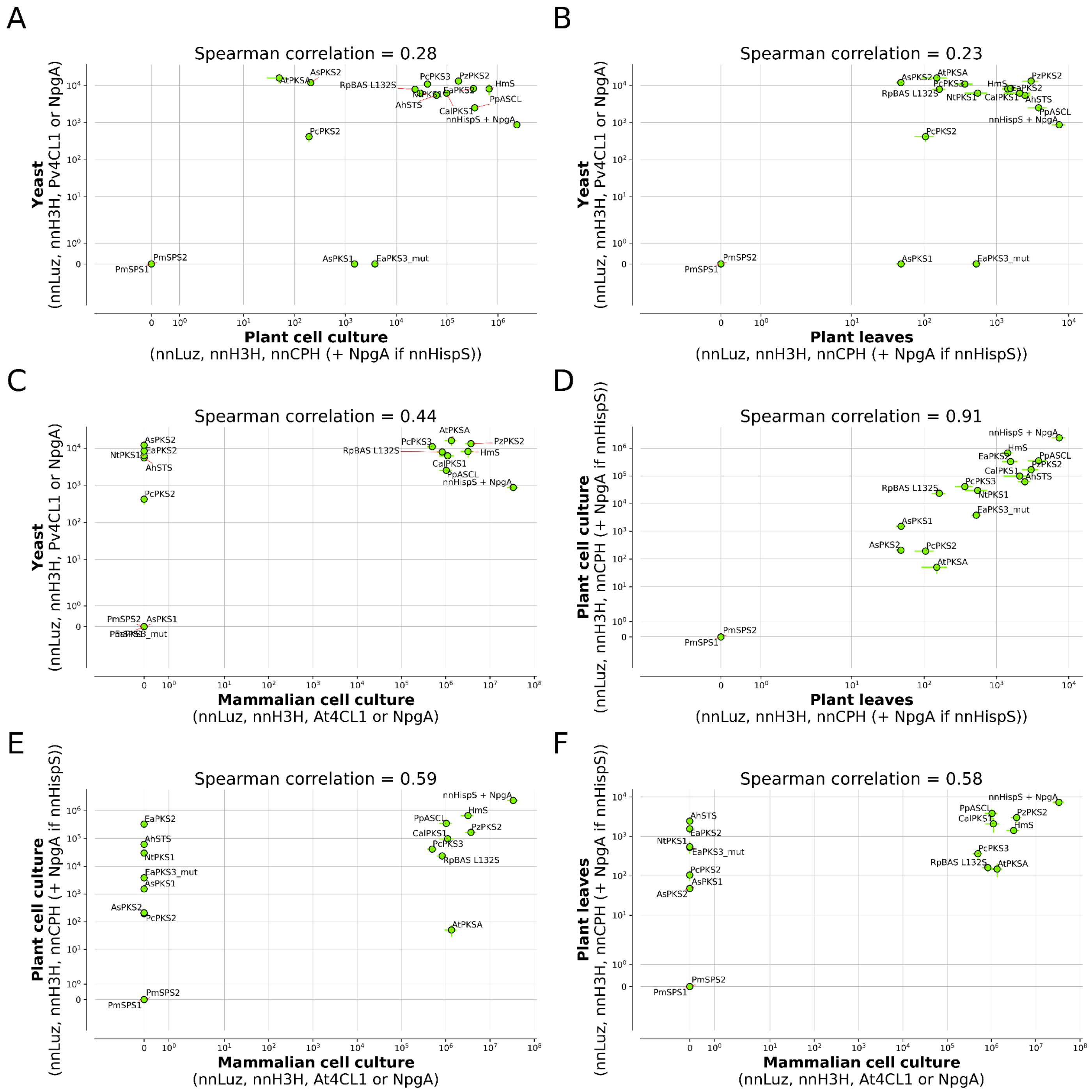

Fig. S13. Correlation of luminescence in different expression systems. The data are shown as mean (green dots) ± SE (green lines). N = 9-18 replicates for yeast data, 10 replicates for plant cell culture, 9 replicates for mammalian cell culture and 11-42 replicates for plant leaves.

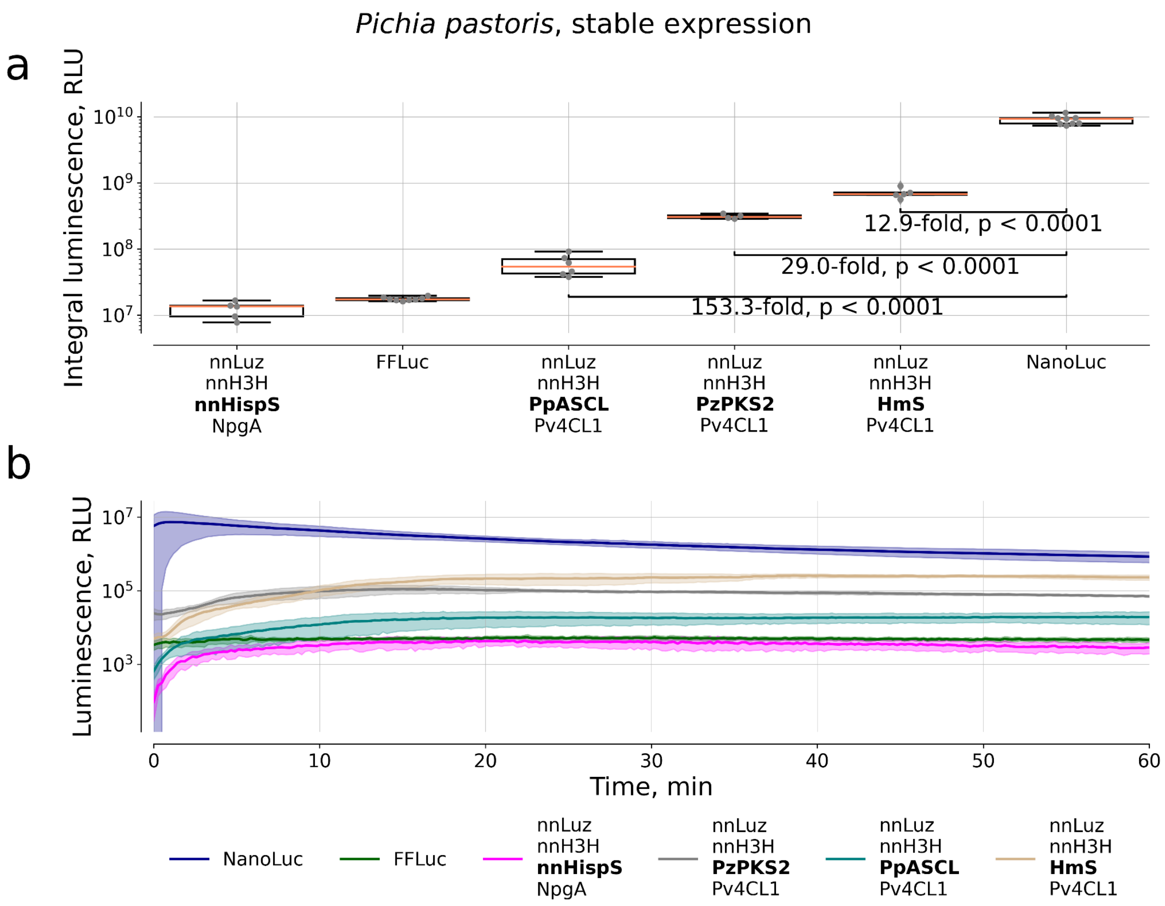

Fig. S14. Luminescence of yeast strains expressing hybrid versions of the autoluminescence pathway (co-expression of HmS, PzPKS2, or PpASCL with Pv4CL1, nnLuz, and nnH3H), all-fungal version of the pathway (NpgA, nnHispS, nnLuz, nnH3H), firefly luciferase FFluc, or Nanoluc. Acquisition started after addition of 100 µM of exogenous substrate (for Nanoluc, recommended concentration was used). **A.** Integral luminescence signal collected for 60 minutes. N = 4-9. The difference between mean values and p-values of post-hoc Conover tests are indicated below the brackets between the box plots. **B** - Luminescence kinetics. Data shown as mean (solid line) ± SD (area around the solid line).

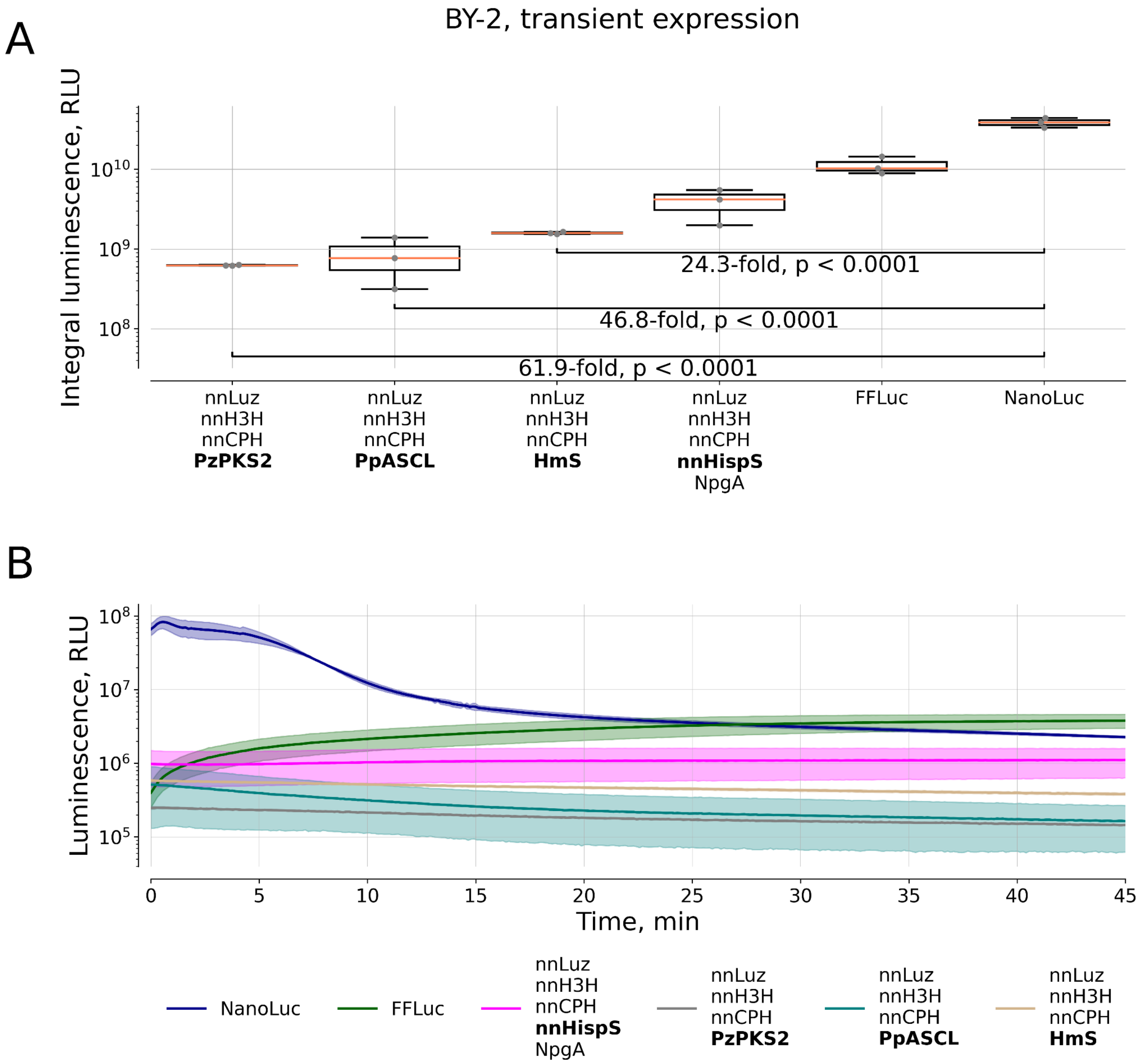

Fig. S15. Luminescence of BY-2 cells expressing hybrid versions of the autoluminescence pathway (co-expression of HmS, PzPKS2, or PpASCL with nnLuz and nnH3H), all-fungal version of the pathway (NpgA, nnHispS, nnLuz, nnH3H), firefly luciferase FFluc, or Nanoluc. All samples additionally co-expressed silencing inhibitor P19. Acquisition started after addition of 100 μM of exogenous substrate for FFluc (for Nanoluc, recommended concentration was used). **A.** Integral luminescence signal registered for 60 minutes. N = 3. The difference between mean values and p-values of post-hoc Conover tests are indicated below the brackets between the box plots. **B** - Luminescence kinetics. Data shown as mean (solid line) ± SD (area around the solid line).

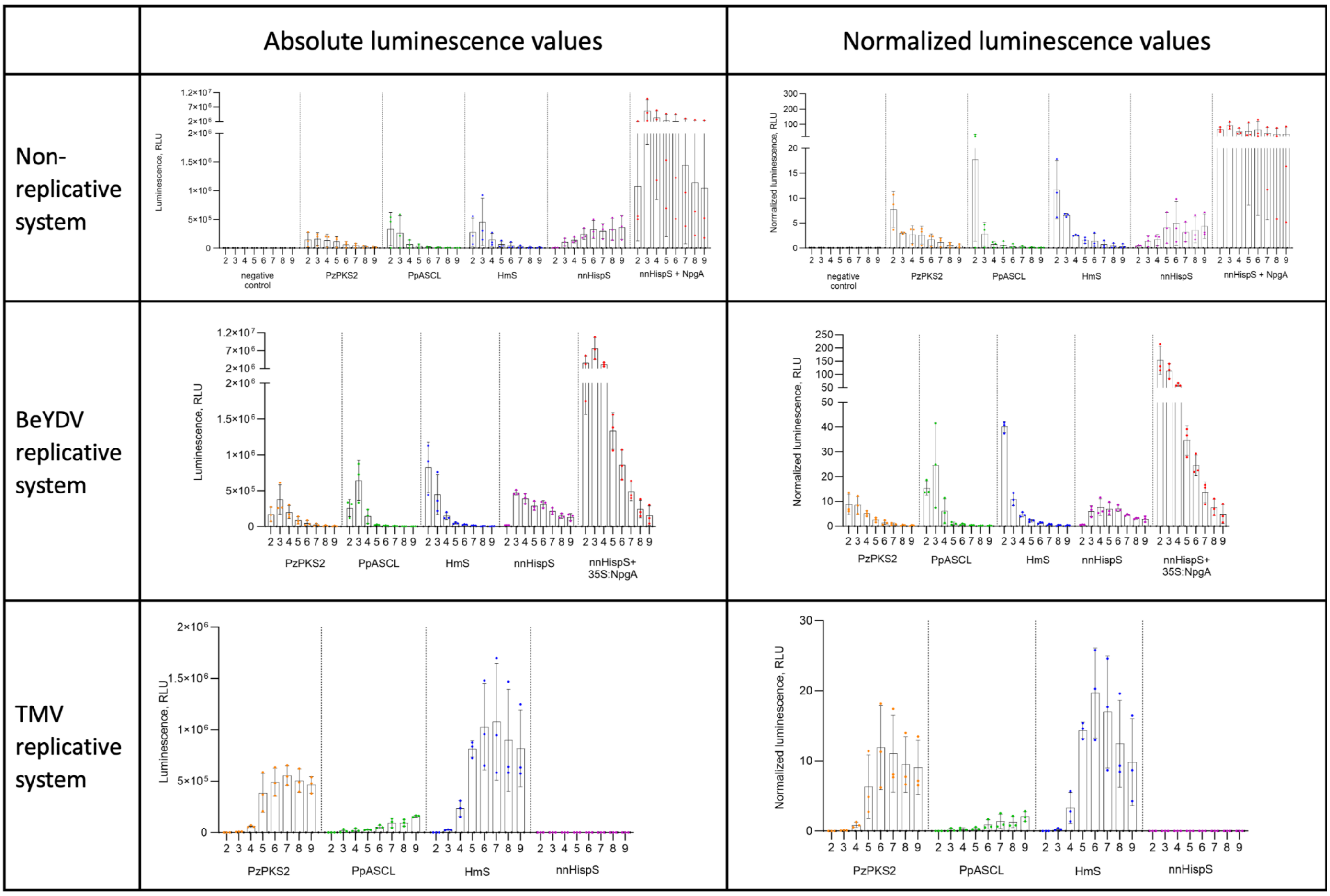

Fig. S16. Luminescence of *Nicotiana benthamiana* leaves transiently expressing the polyketide synthases PzPKS2, PpASCL, HmS, and nnHispS through a non-replicative system (standard binary vector), BeYDV replicative system and TMV replicative system. Measurements were taken from 2 to 9 days post-infiltration. Every polyketide synthase is co-expressed with nnLuz, nnH3H, nnCPH, P19, and enhanced GFP (eGFP) (and ± NpgA in a non-replicative configuration in the case of nnHispS). Graphs on the left column show the absolute luminescence values (arbitrary units, a.u.) of leaf discs, and graphs on the right column show the luminescence/fluorescence ratios normalized with the luminescence/fluorescence ratios conferred by a pNos:nnHispS construct. An empty binary vector was used as a negative control. Error bars indicate SD (n=3).

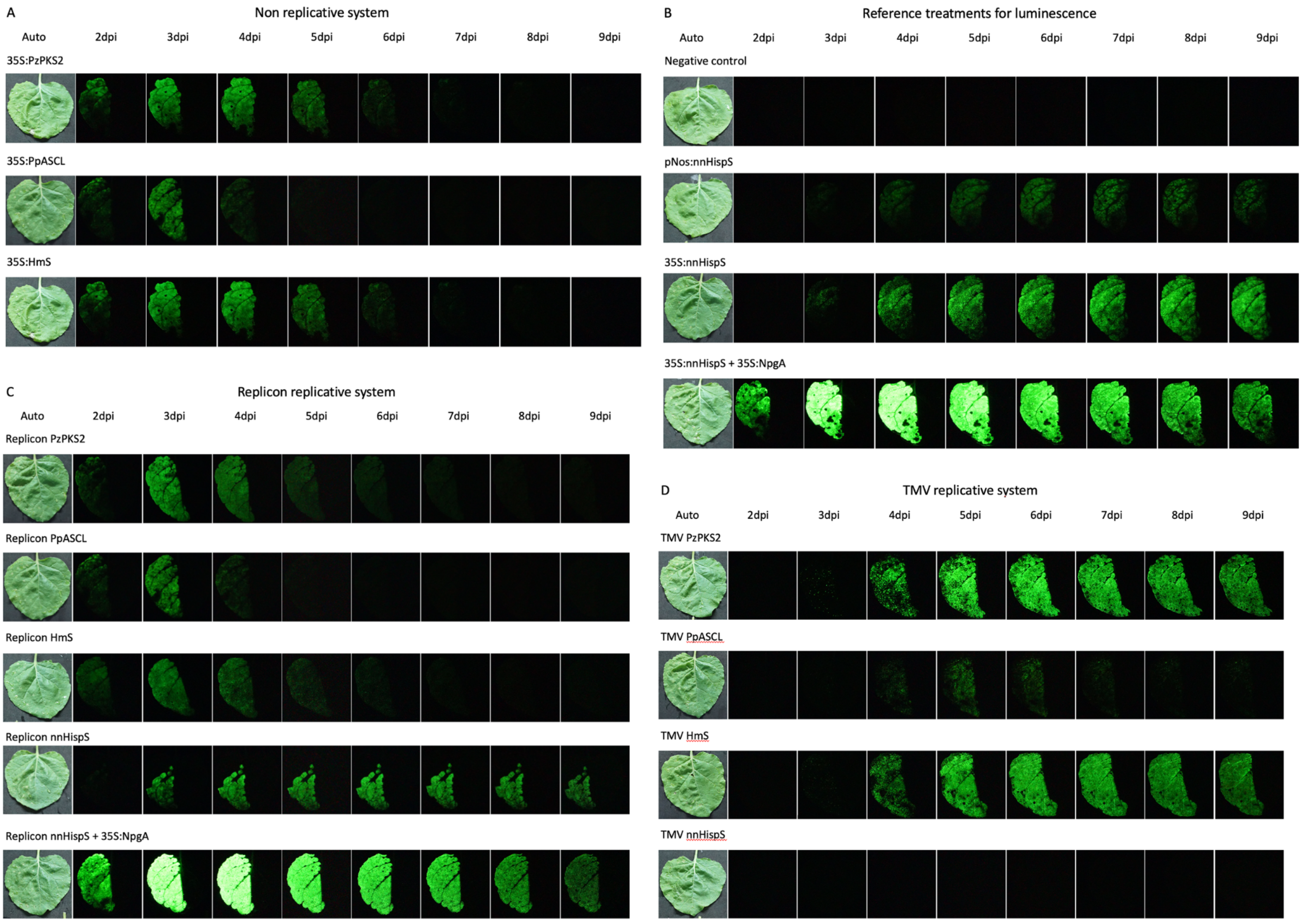

Fig. S17. Time course in transiently transformed *N. benthamiana* leaves expressing the polyketide synthases PzPKS2, PpASCL, and HmS through a non-replicative system (standard binary vector) (A), a BeYDV replicative system (C) and a TMV replicative system (D). Reference treatments for luminescence are included in panel B, where the negative control (empty vector) and nnHispS are transiently transformed in N. benthamiana via standard binary vectors. Every polyketide synthase is co-expressed with nnLuz, nnH3H, nnCPH, and P19 (and ± NpgA in a non-replicative configuration in the case of nnHispS). Pictures were taken with an exposure time of 30 seconds from 2 to 9 days post-infiltration (dpi).

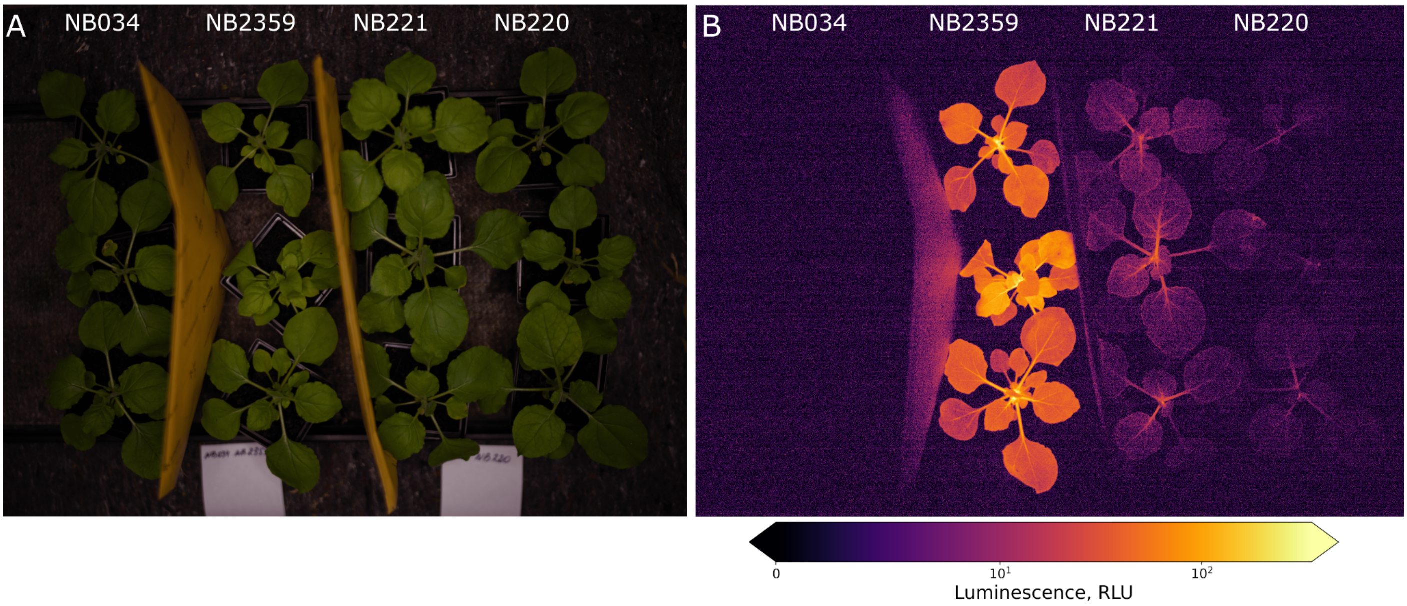

Fig. S18. Transgenic 5-6 week-old *N. benthamiana* plants expressing different versions of the bioluminescence pathway. Photo of transgenic lines NB034 (expressing nnLuz, nnH3H, nnCPH), NB2359 (expressing nnLuz, nnH3H, nnCPH, nnHispS, npgA), and lines NB221 and NB220 (expressing PpASCL, nnLuz, nnH3H, and nnCPH) in ambient light (A) and in the dark (B).

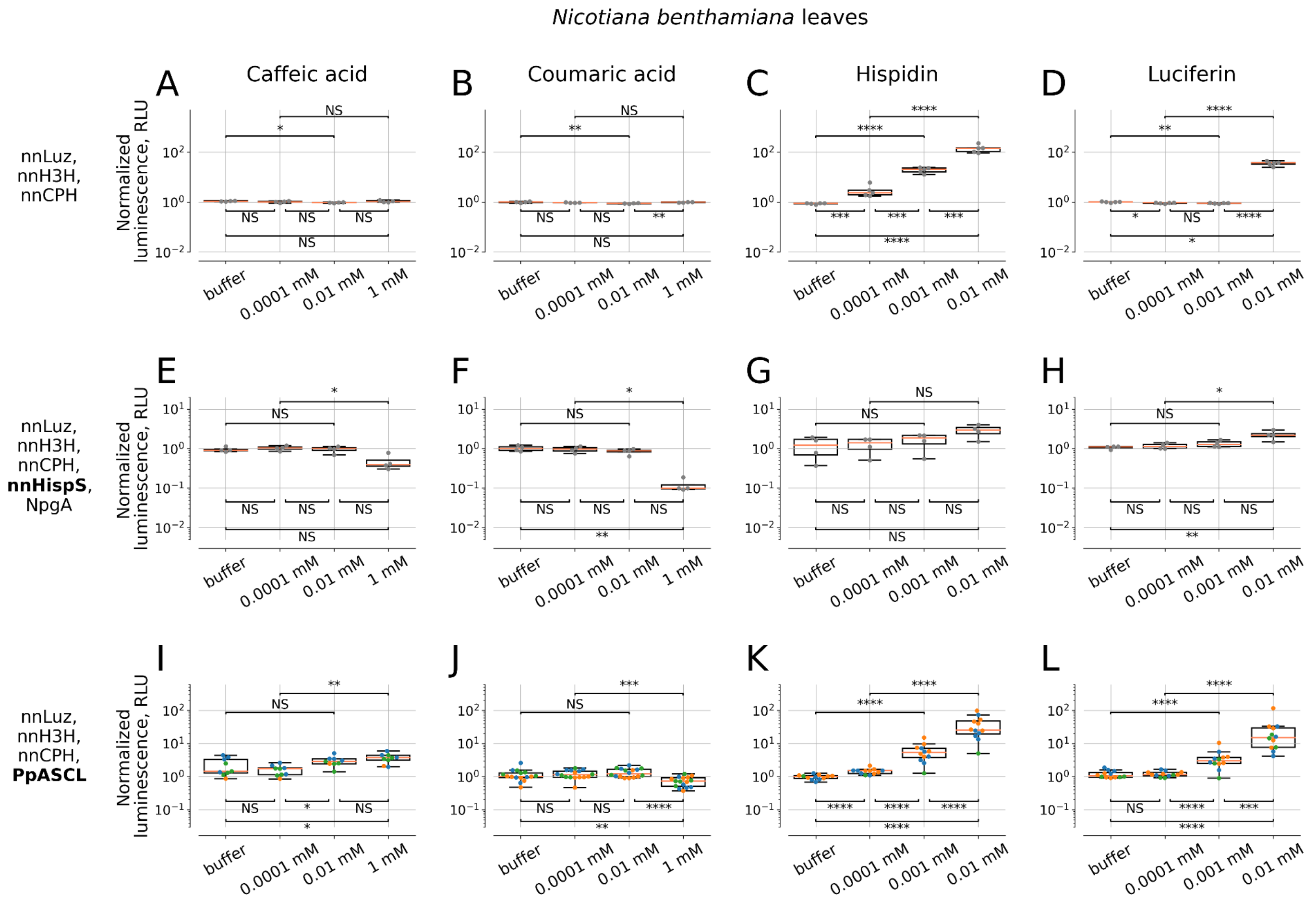

Fig. S19. Injections of caffeic acid, coumaric acid, hispidin and luciferin into leaves of *N. benthamiana* lines constitutively expressing nnLuz, nnH3H, nnCPH, and without polyketide synthase (**A-D**), with nnHispS and NpgA (**E-H**) or with PpASCL (**I-L**). N = 4-16 leaves per box plot. If not grey, the colour indicates data points from different plant lines. The p-values of the post-hoc Conover test are indicated near the brackets between the box plots (**** - p ≤ 0.0001, *** - 0.0001 < p ≤ 0.001, ** - 0.001 < p ≤ 0.01, * - 0.01 < p ≤ 0.05, NS (not significant) - 0.05 < p).

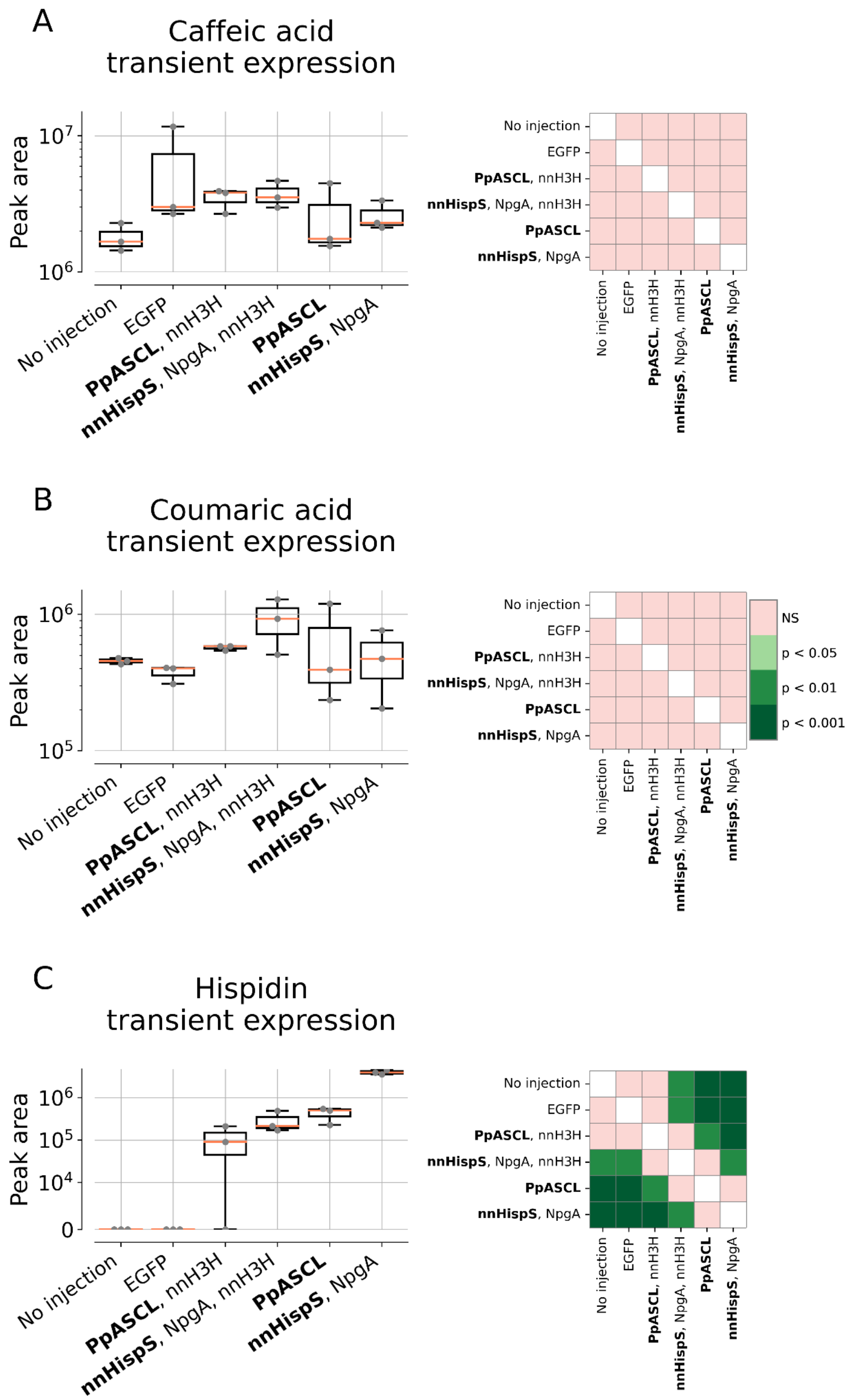

Fig. S20. Abundance of caffeic acid (A), coumaric acid (B), and hispidin (C) in leaves of *N. benthamiana* transiently expressing hispidin biosynthesis genes, with or without nnH3H, or EGFP control.

The abundance of metabolites is represented by LCMS peak area values. The boxes are the first and the third quartiles, whiskers are the rest of the distribution except outliers, the orange line is the median.

Box-and-whiskers plots are accompanied by colour-coded p-values of post-hoc two-sided Conover's test corrected by the step-down method using Sidak adjustments, NS — non-significant. Kruskal-Wallis H Test: H-statistic = 8.18, $p = 0.15$ (A), H-statistic = 7.57, $p = 0.18$ (B), H-statistic = 15.76, $p = 7.6e-3$ (C). N = 3 biologically independent samples per box plot.

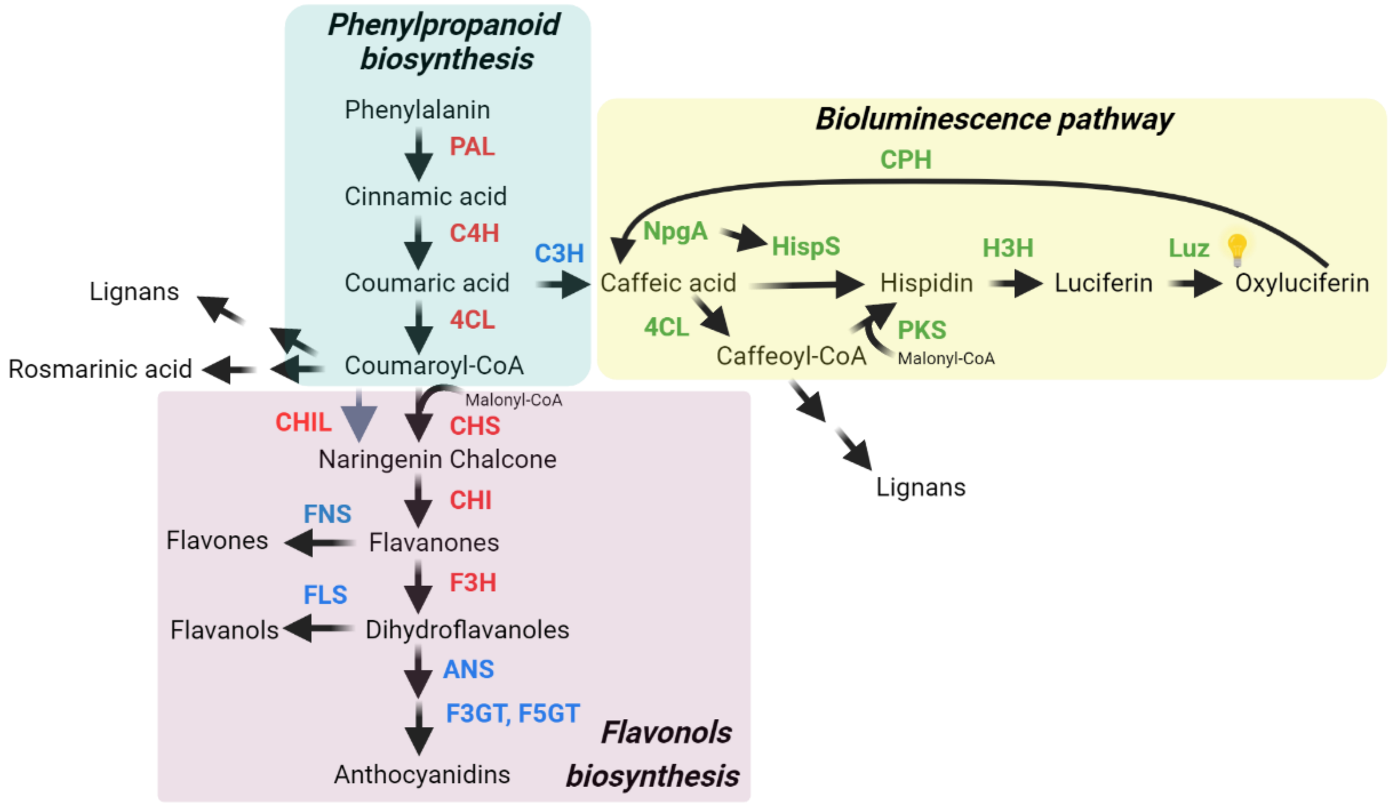

Fig. S21. Plant metabolic pathways: phenylpropanoid and flavonols biosynthesis connected with bioluminescence reaction. Caffeic acid affected enzymes marked with green, enzymes that we targeted by silencing or activation in this work are marked with red. PAL - phenylalanine ammonia-lyase 4, C4H - cinnamate-4-hydroxylase, 4CL - 4-coumaroyl-CoA ligase, CHIL - chalcone-flavanone isomerase like protein, CHS - chalcone synthase, CHI - chalcone isomerase, FNS - flavone synthase, FLS - flavonol synthase, ANS - anthocyanidin synthase, F3GT - flavonoid 3-O-glucosyltransferase, C3H - coumarate 3-hydroxylase, NpgA - 4'-phosphopantetheinyl transferase, HispS - fungal hispidin synthase, PKS - polyketide synthase, H3H - fungal hispidin-3-hydroxylase, Luz - fungal luciferase, CPH - caffeoyl pyruvate hydrolase.

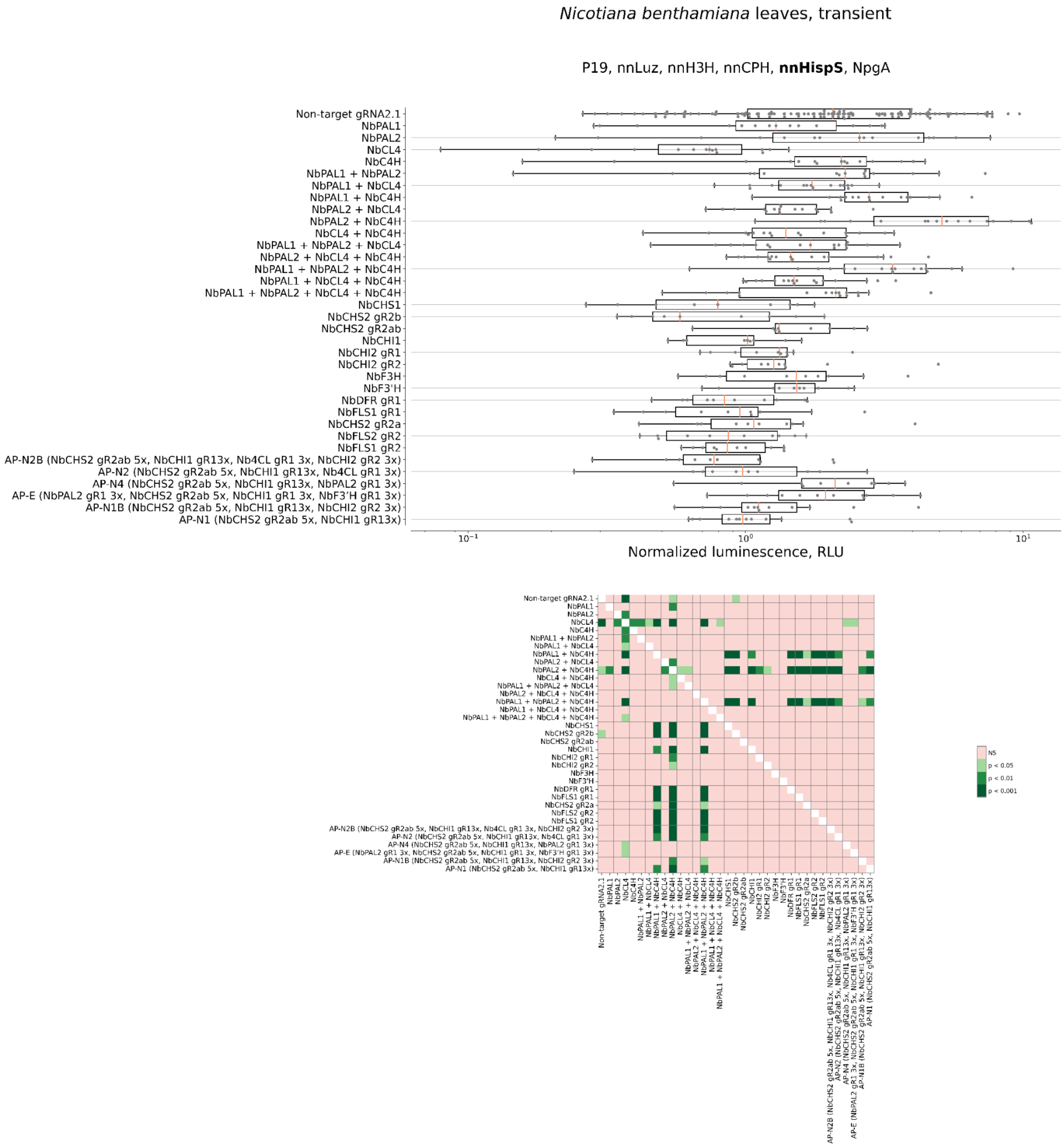

Fig. S22. dCasEV2.1-mediated transcriptional activation of individual genes of the flavonoid pathway and its combinations in *N. benthamiana* wild type agroinfiltrated with P19, nnLuz, nnH3H, nnCPH, nnHispS and NpgA. Box-and-whiskers plots are accompanied by colour-coded p-values of Conover's test, NS — non-significant. Kruskal-Wallis H Test: H-statistic = 156.77, p = 1.1e-17. N = 9-99 leaves per box plot.

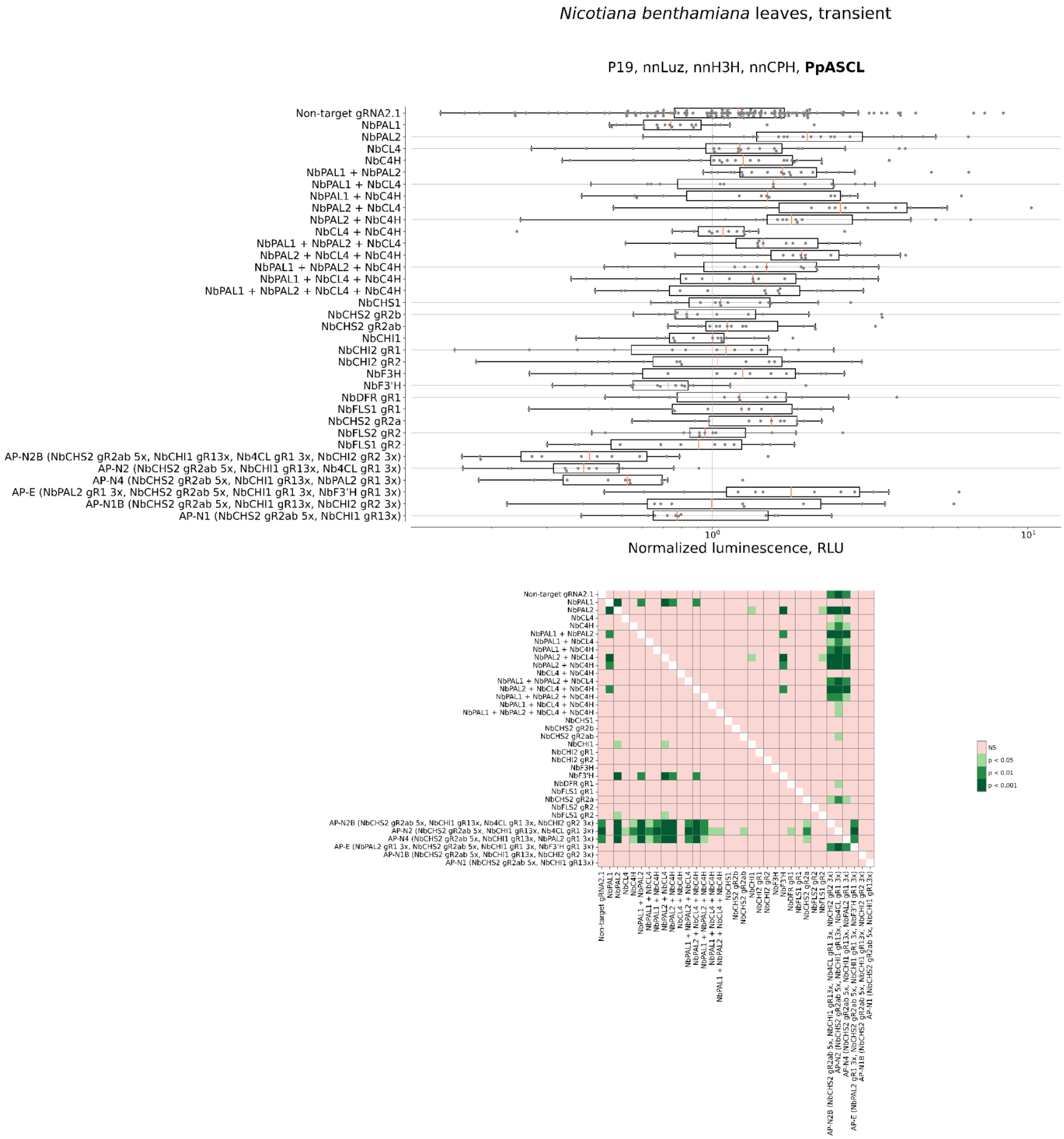

Fig. S23. dCasEV2.1-mediated transcriptional activation of individual genes of the flavonoid pathway and its combinations in *N. benthamiana* wild type agroinfiltrated with P19, nnLuz, nnH3H, nnCPH, PpASCL. Box-and-whiskers plots are accompanied by colour-coded p-values of Conover's test, NS — non-significant. Kruskal-Wallis H Test: H-statistic = 99.69, p = 2.3e-8. N = 12-115 leaves per box plot.

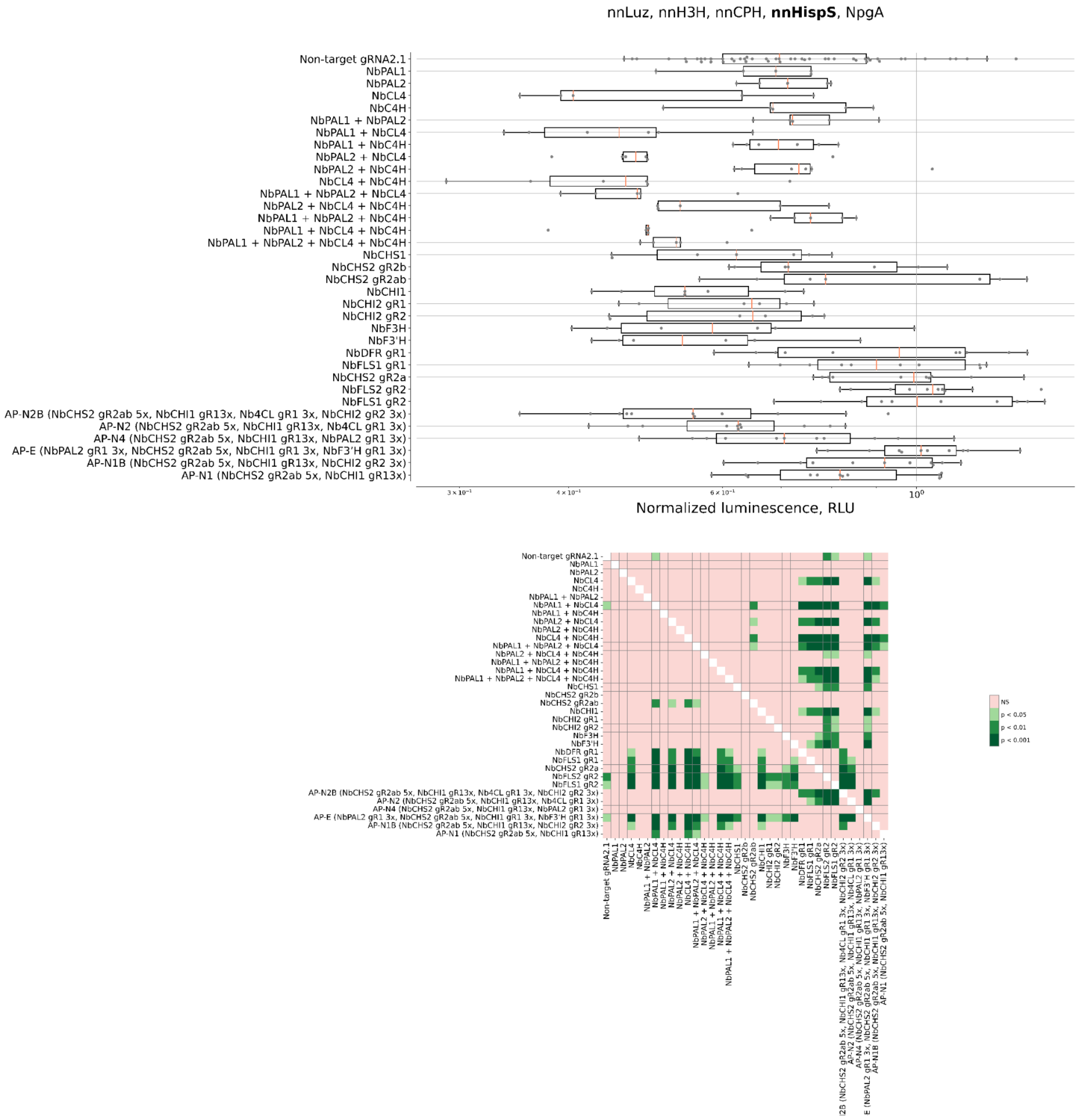

Fig. S24. dCasEV2.1-mediated transcriptional activation of individual genes of the flavonoid pathway and its combinations in *N. benthamiana* constitutively expressing nnLuz, nnH3H, nnCPH, and nnHispS (with NpgA). Box-and-whiskers plots are accompanied by colour-coded p-values of Conover's test, NS — non-significant. Kruskal-Wallis H Test: H-statistic = 148.47, p = 2.9e-16. N = 5-50 leaves per box plot.

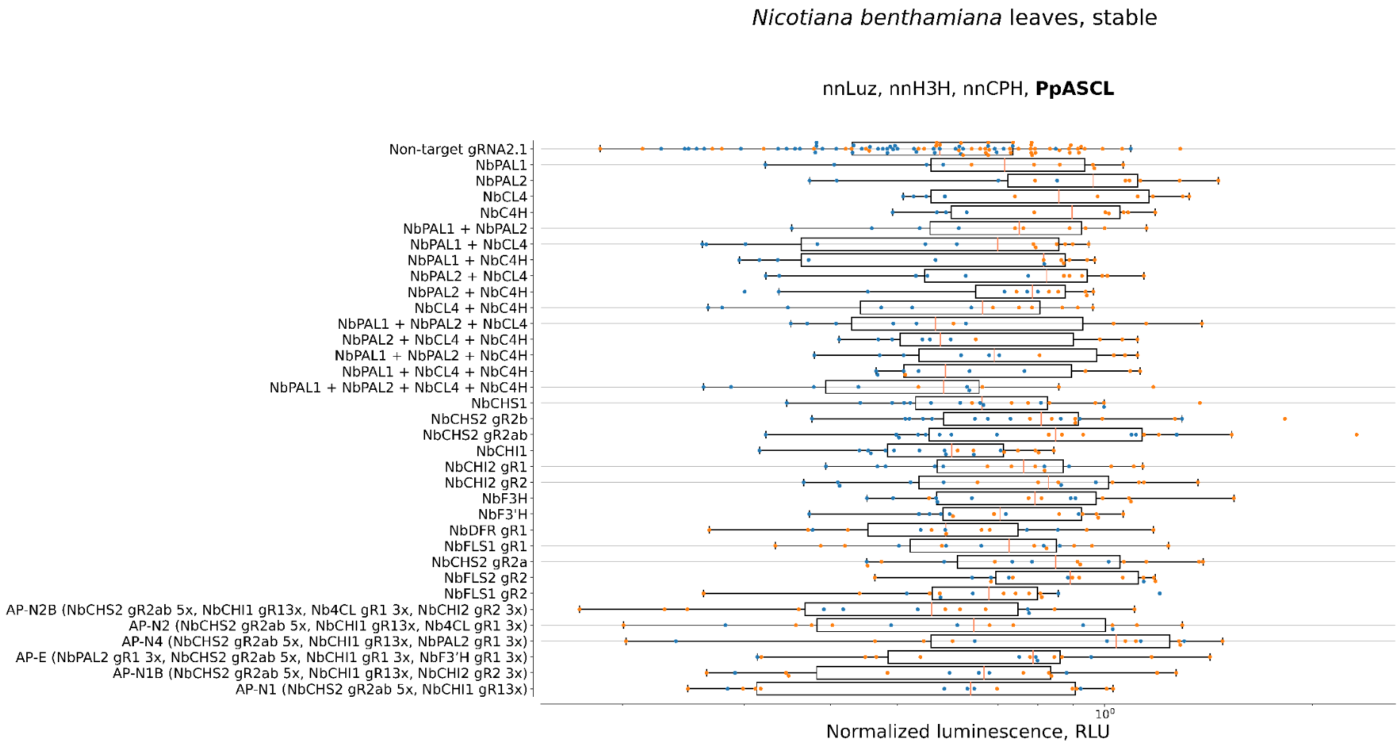

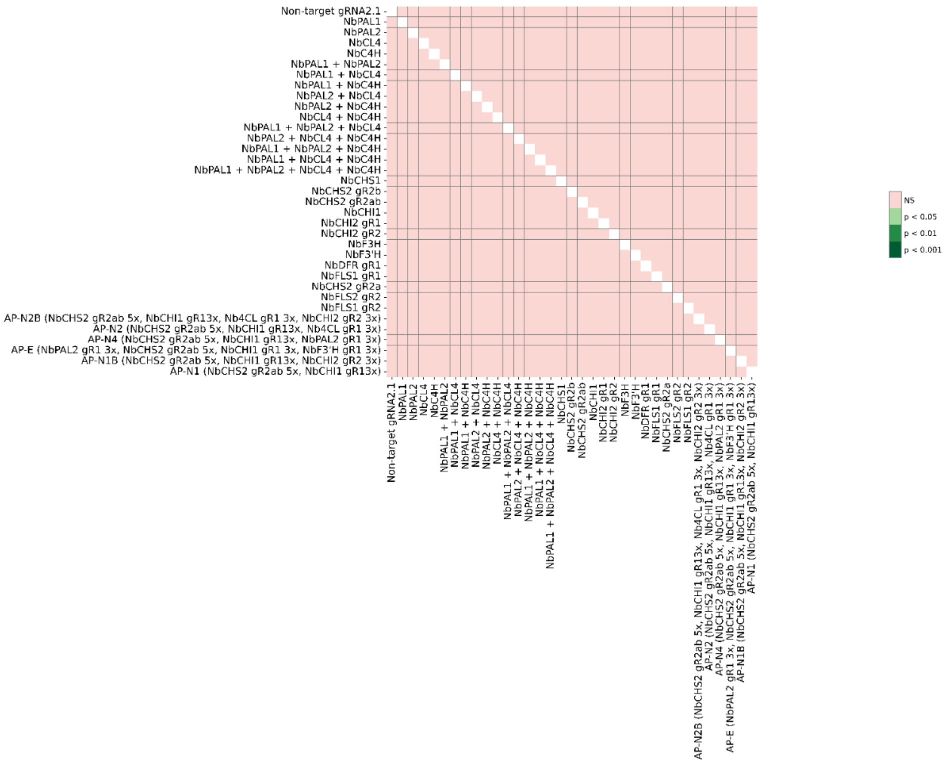

Fig. S25. dCasEV2.1-mediated transcriptional activation of individual genes of the flavonoid pathway and its combinations in *N. benthamiana* constitutively expressing nnLuz, nnH3H, nnCPH, and PpASCL. Box-and-whiskers plots are accompanied by colour-coded p-values of Conover's test, NS — non-significant. Kruskal-Wallis H Test: H-statistic = 44.42, p = 0.. N = 10-92 leaves per box plot. The colour of data points indicates different plant lines.

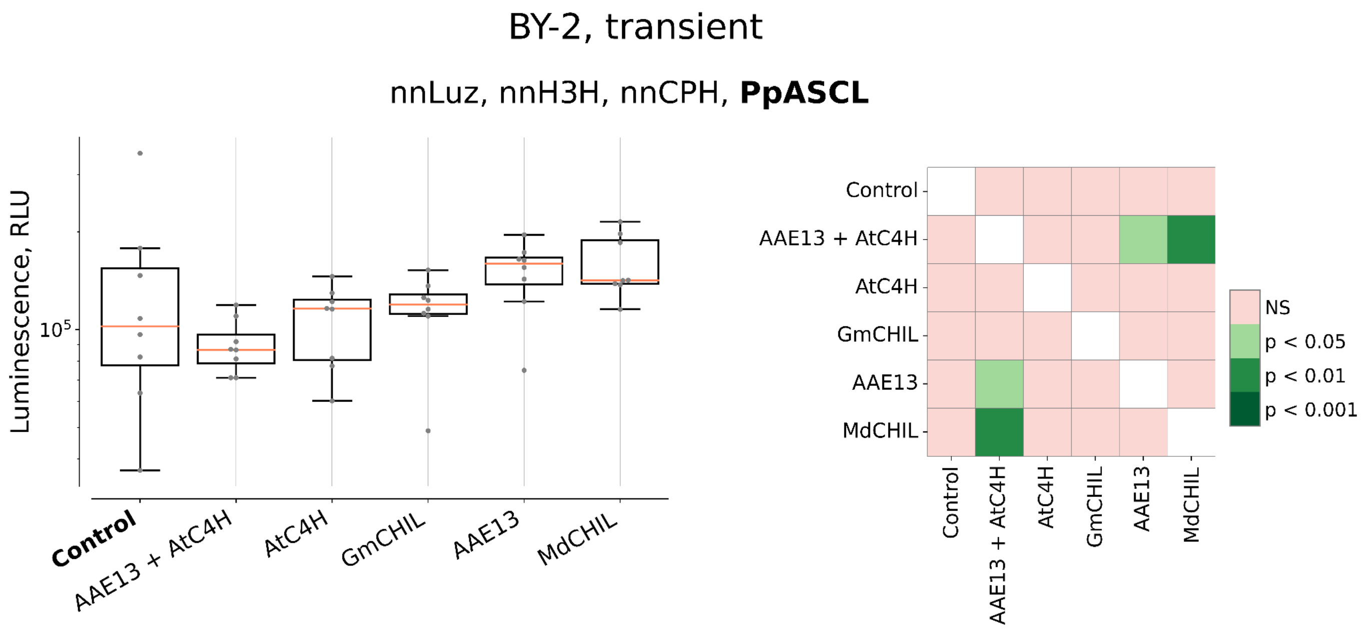

Fig. S26. Enhancing endogenous precursors with overexpression of AAE13 (malonyl-CoA synthetase from *Arabidopsis thaliana*) (45), AtC4H (cinnamate-4-hydroxylase from *Arabidopsis thaliana*), GmCHIL (chalcone isomerase-like protein from *Glycine max*) (46), MdCHIL (chalcone isomerase-like protein from *Malus domestica*) (46) in BY-2 plant cell packs transiently expressing P19, nnLuz, nnH3H, nnCPH, and PpASCL. Box-and-whiskers plots are accompanied by colour-coded p-values of Conover's test, NS — non-significant. Kruskal-Wallis H Test: H-statistic = 16.44, p = 5.7e-3. N = 8 plant cell packs per box plot.

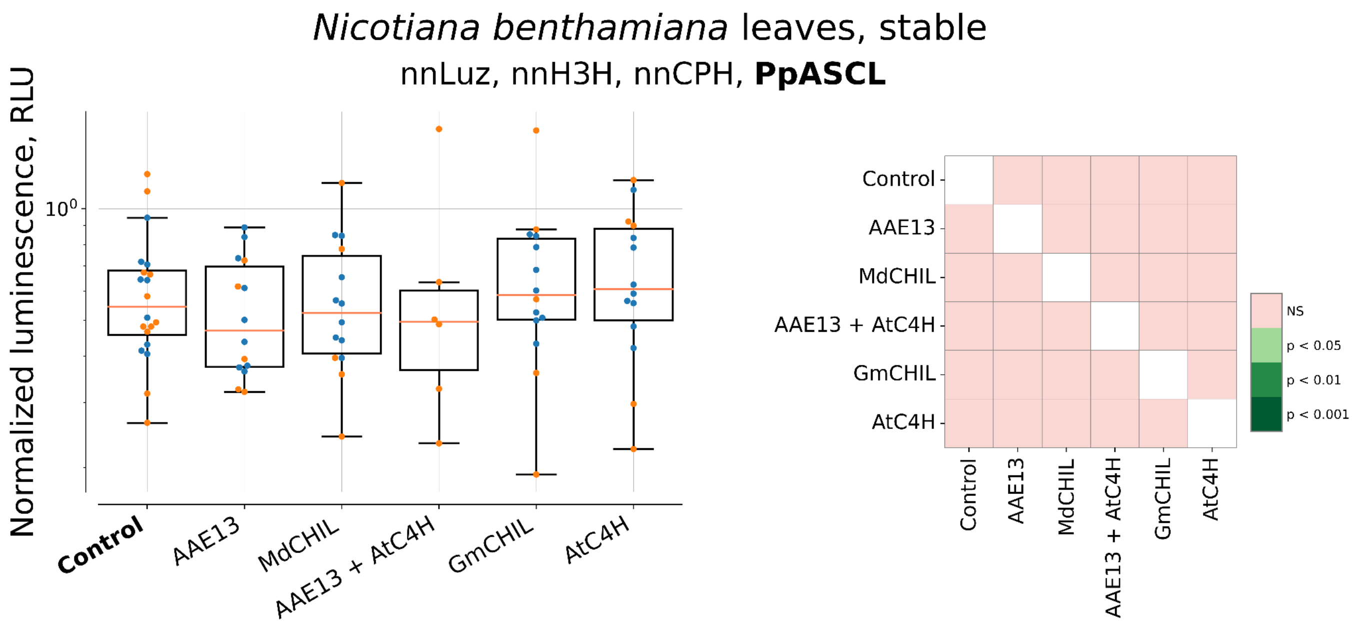

Fig. S27. Enhancing endogenous precursors with overexpression of AAE13 (malonyl-CoA synthetase from *Arabidopsis thaliana*) (45), AtC4H (cinnamate-4-hydroxylase from *Arabidopsis thaliana*), GmCHIL (chalcone isomerase-like protein from *Glycine max*) (46), MdCHIL (chalcone isomerase-like protein from *Malus domestica*) (46) in *N. benthamiana* constitutively expressing P19, nnLuz, nnH3H, nnCPH and PpASCL. Box-and-whiskers plots are accompanied by colour-coded p-values of Conover's test, NS — non-significant. Kruskal-Wallis H Test: H-statistic = 3.09, p = 0.68. N = 6-20 leaves per box plot. The colour of data points indicates different plant lines.

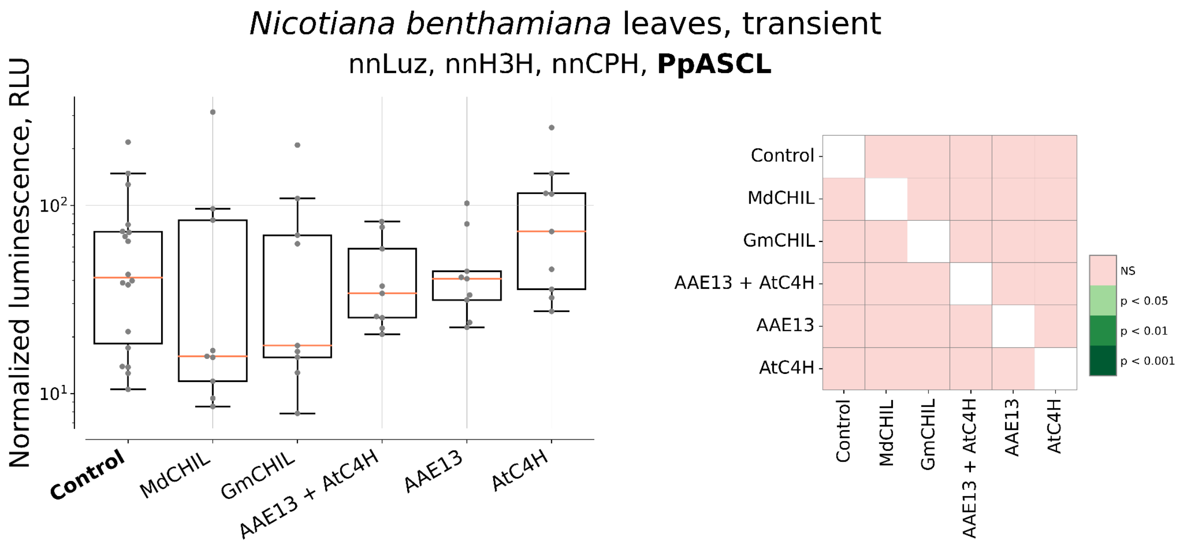

Fig. S28. Enhancing endogenous precursors with overexpression of AAE13 (malonyl-CoA synthetase from *Arabidopsis thaliana*) (*45*), AtC4H (cinnamate-4-hydroxylase from *Arabidopsis thaliana*), GmCHIL (chalcone isomerase-like protein from *Glycine max*) (*46*), MdCHIL (chalcone isomerase-like protein from *Malus domestica*) (*46*) in *N. benthamiana* transiently expressing P19, nnLuz, nnH3H, nnCPH, PpASCL. Box-and-whiskers plots are accompanied by colour-coded p-values of Conover's test, NS — non-significant. Kruskal-Wallis H Test: H-statistic = 6.31, p = 0.28, N = 9-18 leaves per box plot.

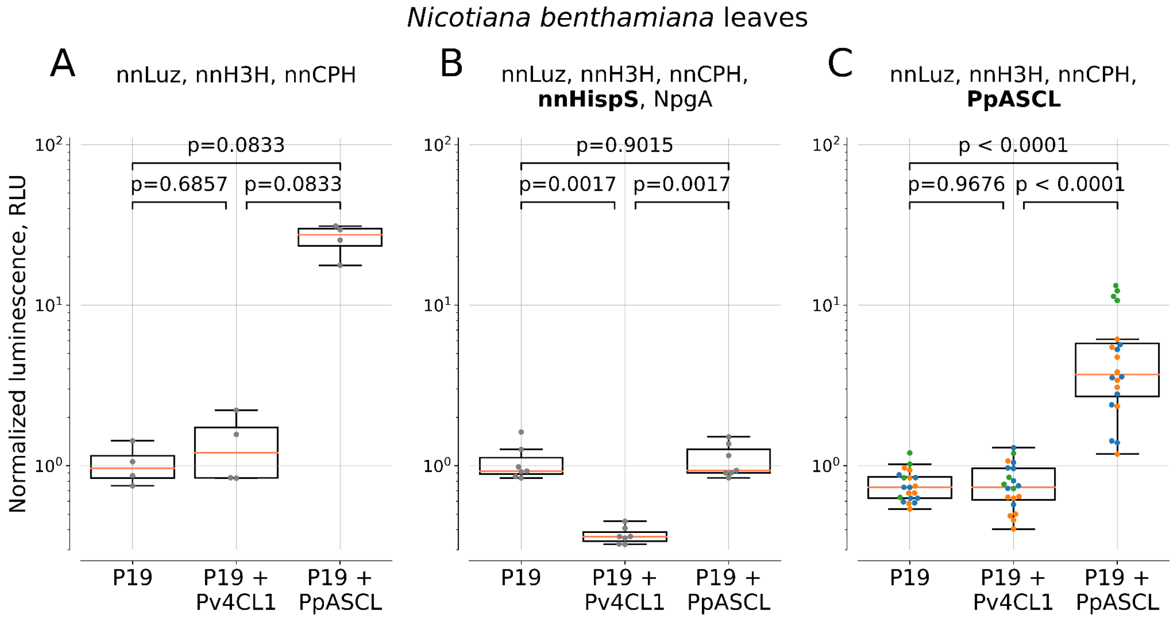

Fig. S29. Overexpression of PpASCL and Pv4CL1 (P19 as a control) by agroinfiltration of *N.benthamiana* constitutively expressing nnLuz, nnH3H, nnCPH (A), with nnHispS and NpgA (B) or PpASCL (C). N = 4-20 leaves per box plot. The colour of data points (if not grey) indicates different plant lines. The p-values of post-hoc Mann-Whitney U-test are indicated on top of the brackets between the box plots.

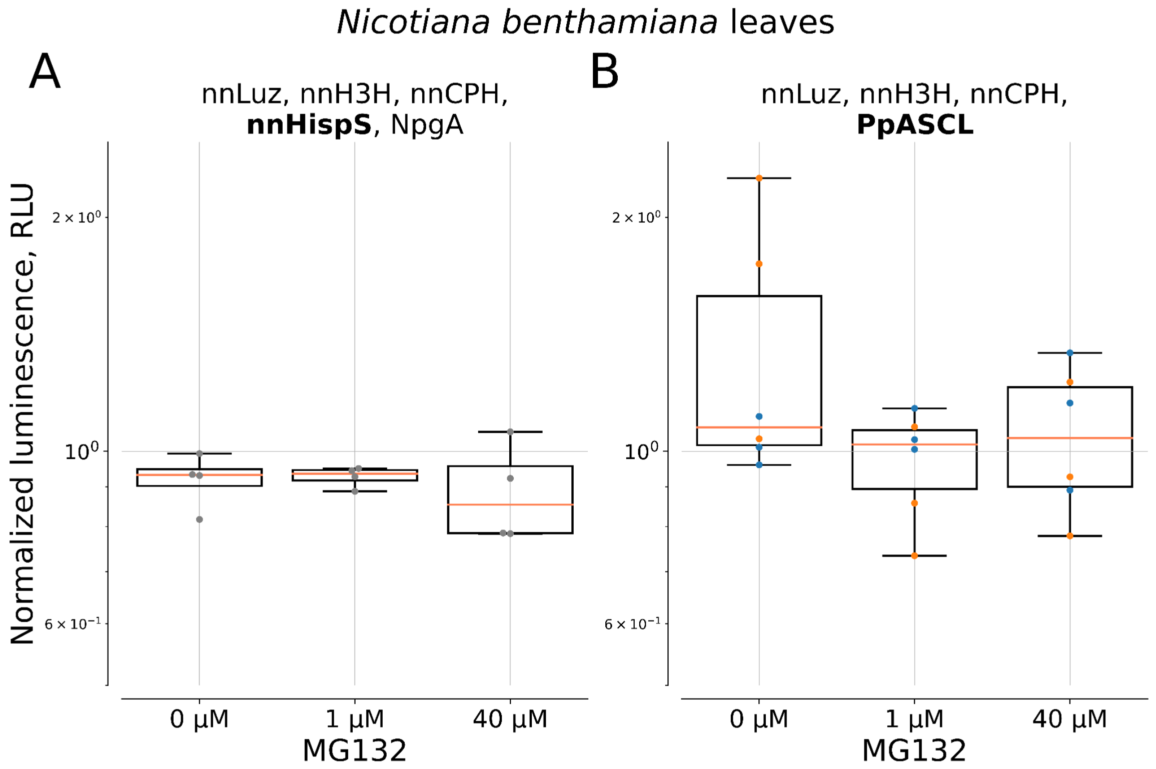

Fig. S30. Testing ubiquitin-dependent degradation of type III polyketide synthases hypothesis. Three concentrations of proteasome inhibitor MG132 (0 µM, 1 µM, 40 µM) were injected into leaves of *N.benthamiana* lines constitutively expressing nnLuz, nnH3H, nnCPH and nnHispS + NpgA (A) or PpASCL (B). Normalised signal obtained 16 hours post-injection is present on the graph. N = 3-6 leaves per box plot. If not grey, colour indicates data points from different plant lines.

BY-2, transient

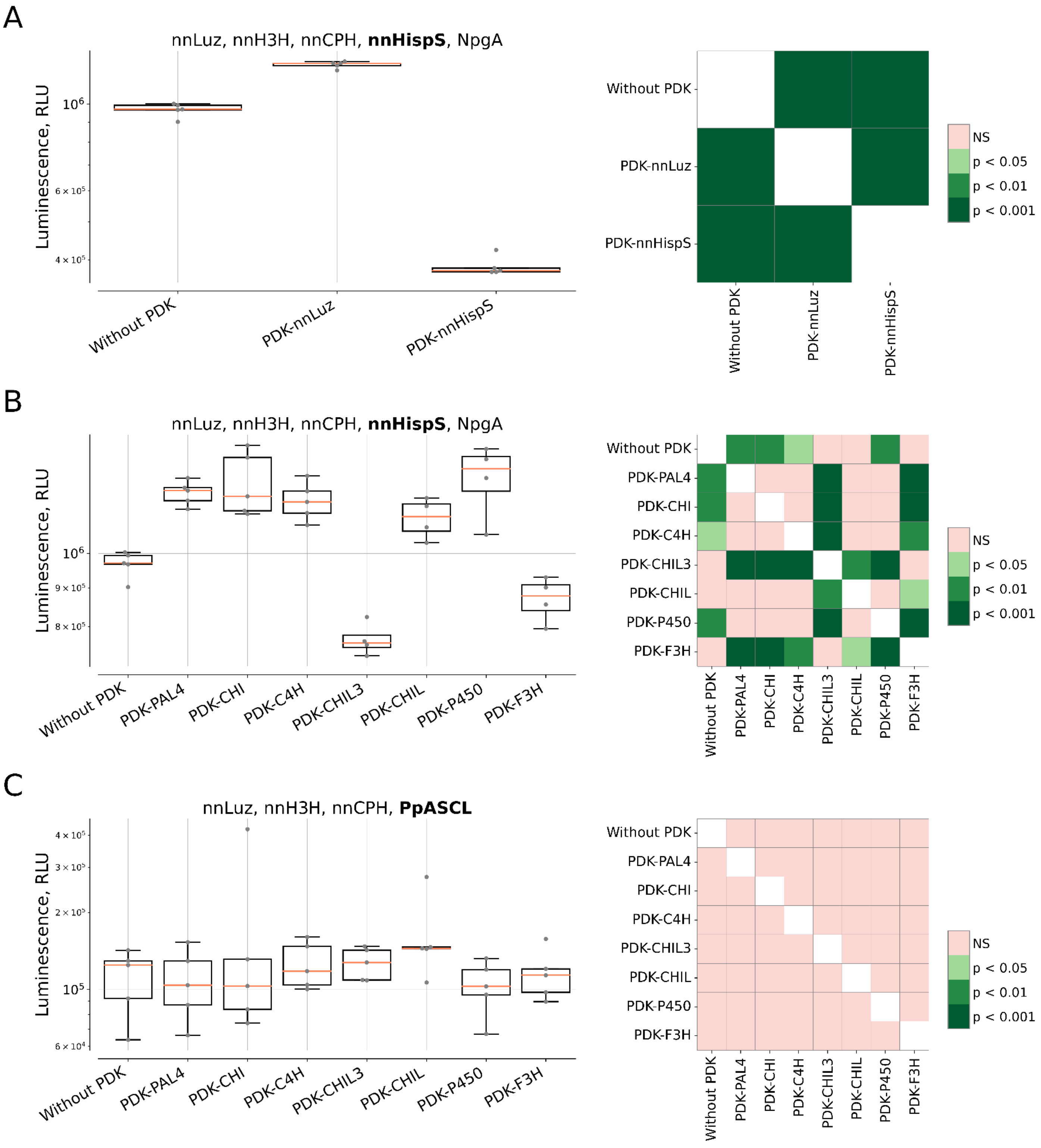

Fig. S31. Silencing testing on bioluminescence system enzymes (nnLuz, nnHispS) in *BY-2 plant cells* agroinfiltrated with nnLuz, nnH3H, nnCPH (A). Silencing of enzymes accepting PKS in metabolons in *N. benthamiana* wild type agroinfiltrated with nnLuz, nnH3H, nnCPH, nnHispS and NpgA (B) or PpASCL (C). The label "without PDK" stands for infiltration with agrobacteria encoding just the bioluminescence genes. Box-and-whiskers plots are accompanied by colour-coded p-values of Conover's test, NS — non-significant. Kruskal-Wallis H Test: H-statistic = 12.5, p = 1.9e-3 (A), H-statistic = 27.48, p = 2.7e-4 (B) or H-statistic = 6.99, p = 0.43 (C). N = 4-5 plant cell packs per box plot.

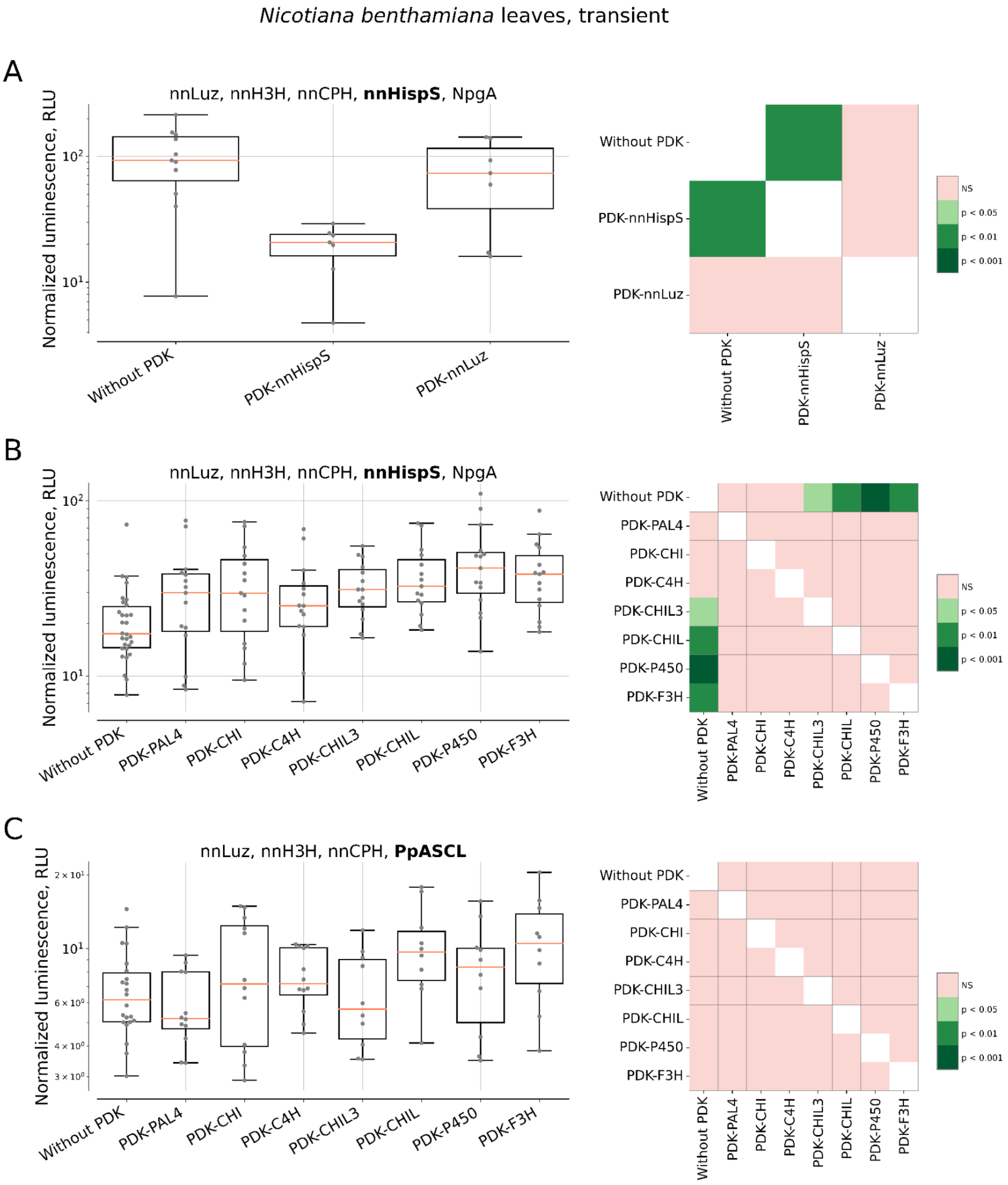

Fig. S32. Silencing testing on bioluminescence system enzymes (nnLuz, nnHispS) in *N. benthamiana* wild type agroinfiltrated with nnLuz, nnH3H, nnCPH, nnHispS, NpgA (A). Silencing of enzymes accepting PKS in metabolons in *N. benthamiana* wild type agroinfiltrated with nnLuz, nnH3H, nnCPH, nnHispS and NpgA (B) or PpASCL (C). “Without PDK” stands for infiltration only with bioluminescent genes without any PDK-constructions .Box-and-whiskers plots are accompanied by colour-coded p-values of Conover’s test, NS

— non-significant. Kruskal-Wallis H Test: H-statistic = 8.74, p = 0.01 (A), H-statistic = 28.09, p = 2.1e-4 (B) or H-statistic = 13.22, p = 0.07 (C). N = 7-30 leaves per box plot.

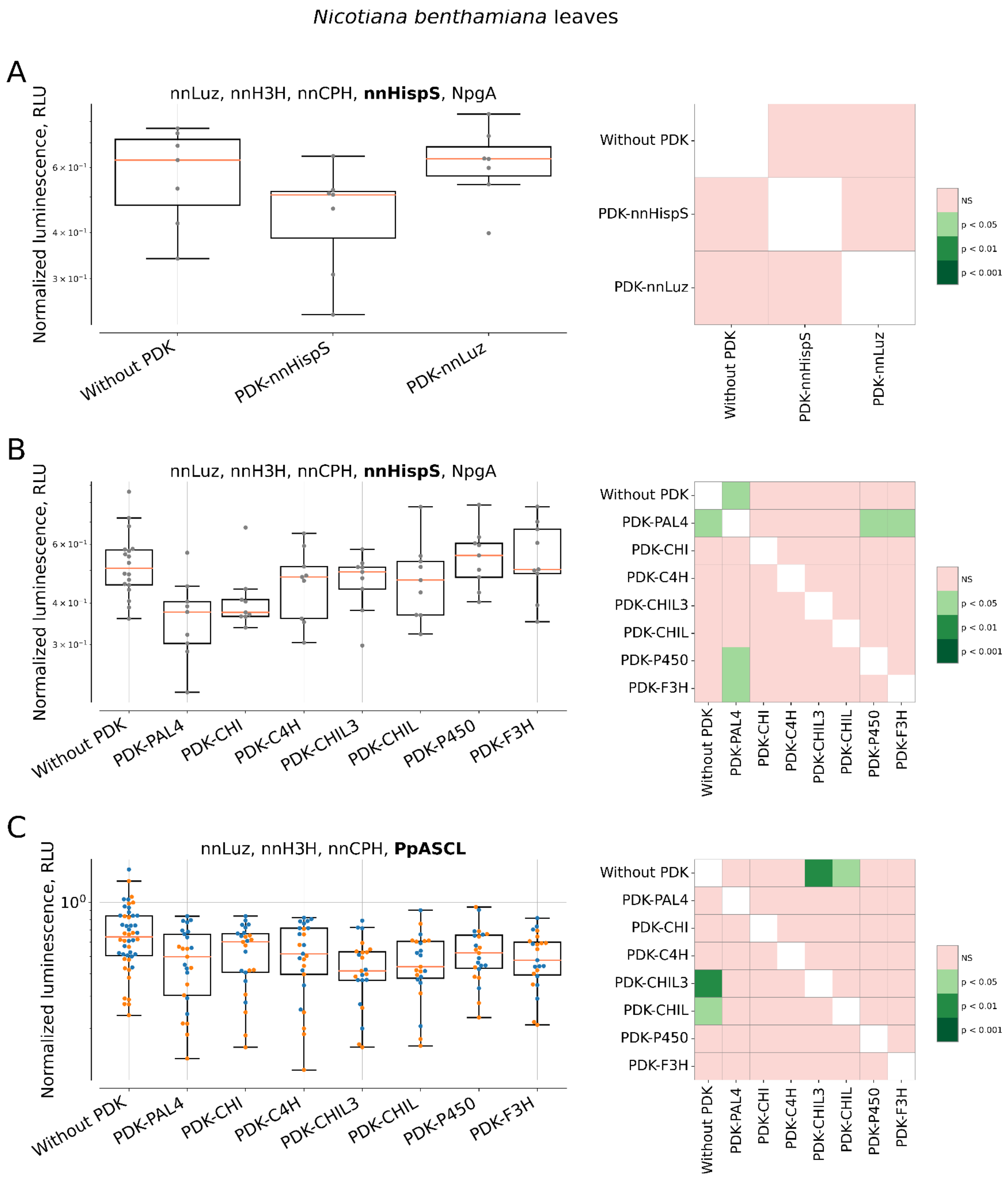

Fig. S33. Silencing testing on bioluminescence system enzymes (nnLuz, nnHispS) in *N. benthamiana* constitutively expressing nnLuz, nnH3H, nnCPH, nnHispS and NpgA (A). Silencing of enzymes accepting PKS in metabolons in *N. benthamiana* constitutively expressing nnLuz, nnH3H, nnCPH, nnHispS and NpgA (B) or PpASCL (C). "Without PDK" stands for infiltration only with bioluminescent genes without any PDK-constructions. Box-and-whiskers plots are accompanied by colour-coded p-values of Conover's test, NS

— non-significant. Kruskal-Wallis H Test: H-statistic = 4.48, $p = 0.1$ (A), H-statistic = 19.13, $p = 7.7e-3$ (B) or H-statistic = 21.82, $p = 2.7e-3$ (C). N = 7-48 leaves per box plot. The colour of data points (if not grey) indicates different plant lines.

Table S1. Selected candidate plant type III polyketide synthases.

Taxonomic data	Polyketide synthase name and origin	ID in this study	Gene sequence	Reported function
Clade: Tracheophytes<br>*Clade: Angiosperms*<br>*Clade:* **Eudicots**<br>*Clade:* **Rosids**<br>*Order: Malvales*<br>*Family: Thymelaeaceae*<br>*Genus: Aquilaria*	*Aquilaria sinensis* polyketide synthase AsPKS1 (PMID 28366630) (*19*)	AsPKS1	MGSQDVAGGALKGVNPGKATILALGKAF<br>PYQLVMQEFLVDGYFKNTSCKDQELKQK<br>LARLCKTTTVKTRYVVMSEEILNKYPELA<br>VEGIPTLKQRLDIGNEALTEMAIEASQACI<br>KKWGRPASEITHLVYVSSSEARLPGGDLY<br>LAQGLGLSPRTKRVVLYFMGCSGGVAGL<br>RVAKDIAENNPGSRVLLATSETTIVGFKPP<br>SAHRPYDLVGVALFGDGAGAMVIGSDPL<br>PGTESPLFELHTAIQNFLPNTEKTIDGRLT<br>EEGISFKLARELPQIVEDHIEGFCGQLTGVI<br>GLSHKQYNKMFWAVHPGGPAILNRVEKR<br>LDLHPNKLDASRRALEDYGNASSNSIVYV<br>LDYMIEETLKMKTESLEPSEWGLILAFGP<br>GVTFEGILARNLAV	Synthesis of hydroxystyrylpyrone compounds
	Aquilaria sinensis polyketide synthase AsPKS2 (PMID 28366630) (*19*)	AsPKS2	MSQAIADNAYRHHLKRAPTPGKATVLAL<br>GKAFPKQVIPQENLVEGYIRDTKCEDVSIK<br>EKLERLCKTTTVKTRYTVMSKEILDNYPE<br>LVTEGSPTIRQRLEIANPAVVEMAKEASLA<br>CIKQWGRPAGDITHIVYVSSSEIRLPGGDL<br>YLANELGLKNDINRIMLYFLGCYGGVTGL<br>RVAKDIAENNPGSRILLTTSETTILGFRPP<br>NKSRPYDLVGAALFGDGAAAVIIGANPEIG<br>RESPFMELNFALQQFLPGTHGVIDGRLSE<br>EGINFKLGRDLPQKIEDNIEDFCRKLMIKA<br>DGDLKEFNELFWAVHPGGPAILNRLESIL<br>DLKNGKLECSRRALMDYGNVSSNTIFYV<br>MEYMREELKREGSEEWGLALAFGPGITF<br>EGILLRSL	
Clade: Tracheophytes<br>*Clade: Angiosperms*<br>*Clade:* **Eudicots**<br>*Clade:* **Asterids**<br>*Order: Cornales*<br>*Family: Hydrangeaceae*<br>*Genus: Hydrangea*	*Hydrangea macrophylla* coumaroyl triacetic acid synthase HmS from GenBank AB011468 (PMID 10469148) (*20*)	HmS	MATKSVAVEEMCKAQKAGGPATILAIGTA<br>VPSNCYYQSEYPDFYFRVTKSDHLTDLKS<br>KFKRMCERSSIKKRYMHLTEEILEENPNM<br>CTFAAPSIDGRQDIVVKEIPKLAKEAASKAI<br>KEWGQPKSNITHLVFCTTSGVDMPGCDY<br>QLTRLLGLRPSIKRLMMYQQGCHAGGTG<br>LRLAKDLAENNKGARVLVVCSEMTVINFR<br>GPSEAHMDSLVGQSLFGDGASAVIVGSD<br>PDLSTEHPLYQIMSASQIIVADSEGAIDGH<br>LRQEGLTFHLRKDVPSLVSDNIENTLVEAF<br>TPILMDSIDSIIDWNSIFWIAHPGGPAILNQ<br>VQAKVGLKEEKLRVSRHILSEYGNMSSAC<br>VFFIMDEMRKRSMEEGKGTTGEGLEWG<br>VLFGFGPGFTVETIVLHSVPI	Synthesis of p-coumaroyl triacetic acid
Clade: Tracheophytes<br>*Clade: Angiosperms*<br>*Clade:* **Eudicots**<br>*Clade:* **Rosids**<br>*Order: Brassicales*<br>*Family: Brassicaceae*<br>*Genus: Arabidopsis*	*Arabidopsis thaliana* polyketide synthase AtPKSA from Uniprot O23674 (PMID 21883237) (*21*)	AtPKSA	MSNSRMNGVEKLSSKSTRRVANAGKAT<br>LLALGKAFPSQVVPQENLVEGFLRDTKCD<br>DAFIKEKLEHLCKTTTVKTRYTVLTREILA<br>KYPELTTEGSPTIKQRLEIANEAVVEMALE<br>ASLGCIKEWGRPVEDITHIVYVSSSEIRLP<br>GGDLYLSAKLGLRNDVNRVMLYFLGCYG<br>GVTGLRVAKDIAENNPGSRVLLTTSETTIL<br>GFRPPNKARPYDLVGAALFGDGAAAVIIG<br>ADPRECEAPFMELHYAVQQFLPGTQNVIE	Synthesis of tetraketide α-pyrone

			GRLTEEGINFKLGRDLPQKIEENIEEFCKKL<br>MGKAGDESMEFNDMFWAVHPGGPAILN<br>RLETKLKLEKEKLESSRRALVDYGNVSSN<br>TILYVMEYMRDELKKKGDAAQEWGLGLA<br>FGPGITFEGLLIRSLTSS	
Division: **Bryophyta**<br>*Class:* **Bryopsida**<br>*Subclass: Funariidae*<br>*Order: Funariales*<br>*Family: Funariaceae*<br>*Genus: Physcomitrella*	*Physcomitrella patens* anther-specific chalcone synthase PpASCL from Uniprot A9TSD3 (PMID 21883237) (*21*)	PpASCL	MASRRVEAAFDGQAVELGATIPAANGNG<br>THQSIKVPGHRQVTPGKTTIMAIGRAVPA<br>NTTFNDGLADHYIQEFNLQDPVLQAKLRR<br>LCETTTVKTRYLVVNKEILDEHPEFLVDGA<br>ATVSQRLAITGEAVTQLGHEAATAAIKEW<br>GRPASEITHLVYVSSSEIRLPGGDLYLAQL<br>LGLRSDVNRVMLYMLGCYGGASGIRVAK<br>DLAENNPGSRVLLITSECTLIGYKSLSPDR<br>PYDLVGAALFGDGAAAMIMGKDPIPVLER<br>AFFELDWAGQSFIPGTNKTIDGRLSEEGIS<br>FKLGRELPKLIESNIQGFCDPILKRAGGLK<br>YNDIFWAVHPGGPAILNAVQKQLDLAPEK<br>LQTARQVLRDYGNISSSTCIYVLDYMRHQ<br>SLKLKEANDNVNTEPEWGLLLAFGPGVTI<br>EGALLRNLC	Synthesis of tetraketide α-pyrone
Clade: Tracheophytes<br>*Clade: Angiosperms*<br>*Clade:* **Eudicots**<br>*Order:* **Caryophyllales**<br>*Family: Polygonaceae*<br>*Genus: Reynoutria*	*Polygonum cuspidatum* polyketide synthase PcPKS3 from GenBank ACC76754 (22)	PcPKS3	MAPAVADIRKAQRAEGPATVLAIGTATPP<br>NCVYQKDYPDYYFRVTNSDHMTDLKEKF<br>RRMCEKSNIEKRYMYLTEEILKENPNMCS<br>YMQTSSLDTRQDMVVSEVPRLGKEAAQK<br>AIKEWGQPKSKITHVIMCTTSGVDMPGA<br>DYQLTKLLGLHPSVKRFMMYQQGCFAGG<br>TVLRLAKDLAENNRGARVLVVCSEITAICF<br>RGPTDTHPDSMVGQALFGDGSGAVIIGA<br>DPDLSIEKPIFELVWTAQTILPDSEGAIDG<br>HLREVGLTFHLLKDVPGLISKNIEKNLTEA<br>FSPLNVSDWNSLFWIAHPGGPAILDQVET<br>KLGLKEEKLKATRQVLNDYGNMSSACVL<br>FIMDEMRKKSVENGHATTGEGLEWGVLF<br>GFGPGLTVETVVLHSVPVAN	Synthesis of bisnoryangonin
	Polygonum cuspidatum (*53*)	PcPKS2	MAASIEEIRKEQTPATVLAIGTANPPNCLY<br>QADFPDYYFRITKSDHLTHLKQKFKRICE<br>NSRIEKRYFQLTEETIKENPNIGAYEAPSL<br>NARHKIQVKGVAELGKEAALEAIKEWGQP<br>KSKITHLIVCCLAGVDMPGTDYQLTKLLDL<br>HPTVKRFMFYHLGCYAGGTVLRLAKDIA<br>ENNKGARVLIVCSEMTAICFRGPSETNIS<br>SMIGTSVLGDGAAAVIVGANPDLTVERPIF<br>ELVWTAQTIVPESDGAVEGHLLESGLSCH<br>LSKTLPVLISNNIEACLSEAFTPLNISDWN<br>SLFWITHPGGPAILDHVEAATGLNKEKLK<br>ATRQVLNDYGNMSSATVFFIMDKMRKRS<br>LENGRATTGEGLEWGVLFGIGPGVTVET<br>VVLRSVPIIH	Synthesis of p-coumaroyl triacetic lacton and bisnoryangonin-type pyrone
Clade: Tracheophytes<br>*Clade: Angiosperms*<br>*Clade:* **Eudicots**<br>*Order:* **Caryophyllales**<br>*Family: Polygonaceae*<br>*Genus: Rheum*	*Rheum palmatum* benzalacetone synthase mutant L132S from UniProt Q94FV7 (PMID 20667730) (*54*)	RpBAS_L132S	MATEEMKKLATVMAIGTANPPNCYYQAD<br>FPDFYFRVTNSDHLINLKQKFKRLCENSR<br>IEKRYLHVTEEILKENPNIAAYEATSLNVR<br>HKMQVKGVAELGKEAALKAIKEWGQPKS<br>KITHLIVCCSAGVDMPGADYQLTKLLDLD<br>PSVKRFMFYHLGCYAGGTVLRLAKDIAE<br>NNKGARVLIVCSEMTTTCFRGPSETHLD<br>SMIGQAILGDGAAAVIVGADPDLTVERPIF<br>ELVSTAQTIVPESHGAIEGHLLESGLSFHL<br>YKTVPTLISNNIKTCLSDAFTPLNISDWNS<br>LFWIAHPGGPAILDQVTAKVGLEKEKLKV<br>TRQVLKDYGNMSSATVFFIMDEMRKKSL<br>ENGQATTGEGLEWGVLFGFGPGITVETV<br>VLRSVPVIS	Synthesis of benzalacetone
Clade: Tracheophytes<br>*Clade: Angiosperms*	*Rheum tataricum* stilbene synthase	RtSTS	MAPEESRHAETAVNRAATVLAIGTANPP<br>NCYYQADFPDFYFRATNSDHLTHLKQKF	Synthesis of stilbene-like polyketide (resveratrol)

Clade: **Eudicots**<br>*Order:* **Caryophyllales**<br>*Family: Polygonaceae*<br>*Genus: Rheum*	RtSTS from Uniprot Q84Q58 (PMID 12620343) (*24*)		KRICEKSMIEKRYLHLTEEILKENPNIASFE<br>APSLDVRHNIQVKEVVLLGKEAALKAINE<br>WGQPKSKITRLIVCCIAGVDMPGADYQLT<br>KLLGLQLSVKRFMFYHLGCYAGGTVLRLA<br>KDIAENNKEARVLIVRSEMTPICFRGPSET<br>HIDSMVGQAIFGDGAAAVIVGANPDLSIER<br>PIFELISTSQTIIPESDGAIEGHLLEVGLSFQ<br>LYQTVPSLISNCIETCLSKAFTPLNISDWN<br>SLFWIAHPGGRAILDDIEATVGLKKEKLKA<br>TRQVLNDYGNMSSACVFFIMDEMRKKSL<br>ANGQVTTGEGLKWGVLFGFGPGVTVETV<br>VLSSVPLIT	
Clade: Tracheophytes<br>*Clade: Angiosperms*<br>*Clade:* **Monocots**<br>*Clade:* **Commelinids**<br>*Order: Commelinales*<br>*Family: Haemodoraceae*<br>*Genus: Wachendorfia*	*Wachendorfia thyrsiflora* polyketide synthase WtPKS1 from Uniprot Q3ZMG6 (PMID 16496097) (*18*)	WtPKS1	MASTEGIQAYRNNMAEGPATIMAIGTAN<br>PPNVVDASTFPDYYWRVTNSEHLSPEYR<br>VKLKRICERSSIRKRHLVLTEQLLKENPTLT<br>TYVDASYDERQSIVLDAVPKLACEAAAKAI<br>KEWGRPKTDITHMVVCTGAGVDVPGVD<br>YKMMNLLGLPPTVNRVMLYNVGCHASG<br>TVLRIAKDLAENNKGARVLVVSSEVSVMF<br>FRGPAEGDVEILLGQALFGDGSAAIIVGAD<br>PIEGVEKPIFQIFSASQMTLPEGEHLVAGH<br>LRELGLTFHLKPQLPNTVSSNIHKPLKKA<br>FEPLNITDWNSIFWIVHPGGRAILDQVQE<br>KIGLEENKLDVSRYVLAENGNMMSASVF<br>FIMDEMRKRSAAQGCSTTGEGHEWGVL<br>FGFGPGLSIETVVLHSVPLSI	Synthesis of tetraketide α-pyrone
Clade: Tracheophytes<br>*Division: Polypodiophyta*<br>*Class: Polypodiopsida*<br>*Subclass:* **Equisetidae**<br>*Order: Equisetales*<br>*Family: Equisetaceae*<br>*Genus: Equisetum*	*Equisetum arvense* transcript EU17351 with first 36aa from oxoglutarate-dependent dioxygenase from Picea sitchensis	EaPKS1	MALQSESQACLGIDAAFILSPEHRPNAKQ<br>CEFTLDQLPVIDLHGLDQPITRQQIVEQIG<br>QACREWGFFQVSNHGLPAELMQSMRDE<br>TRAFFALPMEEKNKLNRHEGNAIGYADIEI<br>TKSIRNWREVFDVAASGEMHMPATYDPA<br>DDSIDRHITRWPEHPSSFRPTCEKYLEAT<br>QGLALNLLELVCESLGVNPRRLHQYFEGN<br>NTSHIRLNHYNACPQPELVLGVGPHQDS<br>GAITVLAVDESVEGLEIKSNKDGQWIRVK<br>DVHPSAFIINLGDMLKVWSNDKYEAVEH<br>RVVTNSAKDRISIVFFLKPSYRADVMPLA<br>ELVDEENPPRYETVNWGRFFKRRIDANY<br>RKPGIVVQQLHHFAINRD	Not reported
	Equisetum arvense transcript EU01786	EaPKS2	MTVLEESADASSRRLAQRANGPATVLAIG<br>TANPANVFEQSSYPDFYFDITNSQHMTEL<br>KLKFSRMCQKSGIKKRYMHLNSEILKAN<br>PSLCAYWEKSLDVRQDIAVVEVPKLGKEA<br>SLKAIKEWGQPKSKITHLVFCTTSGVDMP<br>GADWALTKLLGLRPSVKRLMMYQQGCFA<br>GGTVLRVAKDLAENNKGARVLVVCSEITC<br>VTFRGPSETHLDSLVGQALFGDGAAAVIL<br>GSDPLPEENPCFELHWSGSNILPDSDGAI<br>DGHLREVGLTFHLMKDVPGIISKNIGKVL<br>NDAFRSAFDESGNAEDRPASVNDIFWIAH<br>PGGPAILDQVEEKMKLAPEKMRATRDVL<br>SEYGNMSSACVLFIMDHMRRMSAQNKL<br>QTTGEGLDWGVLLGFGPGLTVETVLLKSI<br>RLAC	Not reported
	Equisetum arvense transcript EU19726 with first 32aa from Equisetum arvense transcript EU19494	EaPKS3_mut	MTILQESSAASPRRLAQRADGPATVLAIG<br>TANPLTAYEQANYPDFYFGITNSNHMTDL<br>KEKFSRMCEKSGIKKRYLHLTEEILKANPS<br>MCGYWEKSLDVRQDIVVVEVPMLARQAS<br>IKAIKEWGQPKSKITHLVFCTTNGLDMPG<br>ADWKLTKLLGLHPNVKRLMIYQQGCFAG<br>GTVMRIAKDLAENNKDARVLVVCSEINLS<br>TFRGPSDIHLDSLVGQALIGDGASAMIIGS<br>DPIPKVETPWFELHWSGSTILPESNGAID<br>GHLREVGLIFHLSKDVPRIISKNIGVLLTDA<br>FEKAFLGGKEAPPSYNDVFWIAHPGGPAI	Not reported

			LDQIEAKLQLKMEKMHASRSILSEYGNM<br>SSASVIFIMDYMRQQAVEKKLATTGEGLD<br>WGVLLGFGPGLTVETILLKSVNLCN	
	Equisetum arvense<br>transcript EU19726	EaPKS3	MTDLKEKFSRMCEKSGIKKRYLHLTEEIL<br>KANPSMCGYWEKSLDVRQDIVVVEVPML<br>ARQASIKAIKEWGQPKSKITHLVFCTTNG<br>LDMPGADWKLTKLLGLHPNVKRLMIYQQ<br>GCFAGGTVMRIAKDLAENNKDARVLVVC<br>SEINLSTFRGPSDIHLDSLVGQALIGDGAS<br>AMIIGSDPIPKVETPWFELHWSGSTILPES<br>NGAIDGHLREVGLIFHLSKDVPRIISKNIGV<br>LLTDAFEKAFLGGKEAPPSYNDVFWIAHP<br>GGPAILDQIEAKLQLKMEKMHASRSILSEY<br>GNMSSASVIFIMDYMRQQAVEKKLATTG<br>EGLDWGVLLGFGPGLTVETILLKSVNLCN	Not reported
Clade: Tracheophytes<br>*Clade: Angiosperms*<br>*Clade:* **Magnoliids**<br>*Order:* **Piperales**<br>*Family: Piperaceae*<br>*Genus: Piper*	*Piper methysticum*<br>*(16)*	PmSPS1	MSKTVEDRAAQRAKGPATVLAIGTATPAN<br>VVYQTDYPDYYFRVTKSEHMTKLKNKFQ<br>RMCDRSTIKKRYMVLTEELLEKNLSLCTY<br>MEPSLDARQDILVPEVPKLGKEAADEAIAE<br>WGRPKSEITHLIFCTTCGVDMPGADYQLT<br>KLLGLRSSVRRTMLYQQGCFGGGTVLRL<br>AKDLAENNAGARVLVVCSEITTAVNFRGP<br>SDTHLDLLVGLALFGDGAAAVIVGADPDP<br>TLERPLFQIVSGAQTILPDSEGAINGHLRE<br>VGLTIRLLKDVPGLVSMNIEKCLMEAFAP<br>MGIHDWNSIFWIAHPGGPTILDQVEAKLG<br>LKEEKLKSTRAVLREYGNMSSACVLFILD<br>EVRKRSMEEGKTTTGEGFDWGVLFGFG<br>PGFTVETVVLHSMPIPKADEGRSG	Synthesis of hispidin reported (not confirmed in this study)
		PmSPS2	MSKMVEEHWAAQRARGPATVLAIGTAN<br>PPNVLYQADYPDFYFRVTKSEHMTQLKE<br>KFKRICDKSAIRKRHLHLTEELLEKNPNIC<br>AHMAPSLDARQDIAVVEVPKLAKEAATKA<br>IKEWGRPKSDITHLIFCTTCGVDMPGADY<br>QLTTLLGLRPTVRRTMLYQQGCFAGGTVL<br>RHAKDFAENNRGARVLAVCSEFTVMNFS<br>GPSEAHLDSMVGMALFGDGASAVIVGAD<br>PDFAIERPLFQLVSTTQTIVPDSDGAIKCH<br>LKEVGLTLHLVKNVPDLISNNMDKILEEAF<br>APLGIRDWNSIFWTAHPGGAAILDQLEAK<br>LGLNKEKLKTTRTVLREYGNMSSACVCF<br>VLDEMRRSSLEEGKTTSGEGLEWGILLGF<br>GPGLTVETVVLRSVPISTANSG	Synthesis of hispidin reported (not confirmed in this study)
Clade: Tracheophytes<br>*Clade: Angiosperms*<br>*Clade:* **Eudicots**<br>*Order:* **Caryophyllales**<br>*Family: Plumbaginaceae*<br>*Genus: Plumbago*	*Plumbago zeylanica*<br>*(55)*	PzPKS2	MAPSVEEIRKAQRAQGPATVLAIGTATPP<br>NCVYQKDYPDYYFRVTNSEHMTELKEKF<br>RRMCDKSMIEKRYMLLTEDLLKENPSMC<br>AYMGSSLDARQDLVVAEVPRLGKEAAQK<br>AIKEWGQPKSKITHVIMCTTSGVDMPGA<br>DYQLTKLLGLRPSVKRFMMYQQGCFAGG<br>TVLRLAKDVAENNKGARVLVVCSEITAICF<br>RGPTDTHLDSMVGQALFGDGSGAVIIGA<br>DPDLTVERPIFELVWTAQTILPDSEGAIDG<br>HLREVGLTLHLLKDVPGLISKNITKALVEA<br>FNPLGISDWNELFWVAHPGGPAILDQVEE<br>KLALKPEKMKATRQVLNDYGNMSSACVL<br>FILDEMRKKSLENGHSTTGEGLEWGVLF<br>GFGPGLTVETVVLHSVPVVN	Synthesis of bisnoryangonin
Clade: Tracheophytes<br>*Clade: Angiosperms*<br>*Clade:* **Eudicots**<br>*Clade:* **Asterids**<br>*Order:* **Solanales**	*Nicotiana tabacum*<br>*(56)*	NtPKS1	MSQNGKNINGASKYFFQPSTRLPTPGKS<br>TILAMGKAFPAQLVPQDCLVEGYIRDTNC<br>QDLAIKEKLERLCKTTTVKTRYTVMSKEIL<br>DKYPELATEGTPTIKQRLEIANPAVVEMA<br>KQASQACIKEWGRSAEEITHIVYVSSSEIR	Synthesis of bisnoryangonin and related triketide pyrones

Family: Solanaceae<br>*Genus: Nicotiana*			LPGGDLYLATELGLRNDIGRVMLYFLGCY<br>GGVTGLRVAKDIAENNPGSRVLLTTSETTI<br>LGFRPPNNARPYDLVGAALFGDGAAAVII<br>GTEPIVGKESPFMELNFATQQFLPGTNNV<br>IDGRLTEEGINFKLGRDLPEKIQDNIEEFCK<br>KLMAKADLKETKYNDLFWAVHPGGPAIL<br>NRLENTLKLQSEKLDCSRRALMDFGNVS<br>SNTIFYVMEYMREELKNKKDEGEEWGLA<br>LAFGPGITFEGILLRSLLERNL	
Clade: Tracheophytes<br>*Clade: Angiosperms*<br>*Clade:* **Eudicots**<br>*Clade:* **Rosids**<br>*Order:* **Fabales**<br>*Family: Fabaceae*<br>*Subfamily: Caesalpinioideae*<br>*Genus: Senna*	*Cassia alata (57)*	CalPKS1	MVKVEEIRKAQRAEGAATVMAIGTATPAN<br>CVEQSTYPDYYFRVTNSEHMTELKEKFQ<br>RMCDKSMIKKRYMHLTEEILKENPNMCA<br>YMAPSIDARQDIVVLEVPKLGKEAATKAIK<br>EWGQPKSKITHLIFCTTSGVDMPGADYQ<br>LTKLLGLRPSVKRYMMYQQGCFAGGTVL<br>RLAKDLAENNKGARVLVVCSEITAVTFRG<br>PSDTHLDSLVGQALFGDGAAAVIVGSDPI<br>PQVETPLFELVWTAQTILPDSEGAIDGHL<br>REVGLTFHLLKDVPGLISKNIEKALVEAFN<br>PLGISDYNSIFWIAHPGGPAILDQVEAKLG<br>LKPEKMQATRHVLSEYGNMSSACVLFIM<br>DEMRRKSTKDGLGTTGEGLEWGVLFGF<br>GPGLTVETVVLHSIAI	Synthesis of bisnoryangonin-like pyrone
Clade: Tracheophytes<br>*Clade: Angiosperms*<br>*Clade:* **Eudicots**<br>*Clade:* **Rosids**<br>*Order:* **Fabales**<br>*Family: Fabaceae*<br>*Subfamily: Faboideae*<br>*Genus: Arachis*	*Arachis hypogaea (58)*	AhSTS	MVSVSGIRKVQRAEGPATVLAIGTANPPN<br>CIDQSTYADYYFRVTNSEHMTDLKKKFQ<br>RICERTQIKNRHMYLTEEILKENPNMCAY<br>KAPSLDAREDMMIREVPRVGKEAATKAIK<br>EWGQPMSKITHLIFCTTSGVALPGVDYEL<br>IVLLGLDPCVKRYMMYHQGCFAGGTVLR<br>LAKDLAENNKDARVLIVCSENTAVTFRGP<br>SETDMDSLVGQALFADGAAAIIIGSDPVPE<br>VEKPIFELVSTDQKLVPGSHGAIGGLLREV<br>GLTFYLNKSVPDIISQNINDALNKAFDPLGI<br>SDYNSIFWIAHPGGRAILDQVEQKVNLKP<br>EKMKATRDVLSNYGNMSSACVFFIMDL<br>MRKRSLEEGLKTTGEGLDWGVLFGFGPG<br>LTIETVVLRSVAI	Synthesis of p-coumaroyl triacetic lacton and bisnoryangonin-like pyrone
Clade: Tracheophytes<br>*Clade: Angiosperms*<br>*Clade:* **Monocots**<br>*Clade:* **Commelinids**<br>*Order: Poales*<br>*Family: Poaceae*<br>*Genus: Oryza*	*Oryza sativa (59)*	OsPKS	MAPTTTMGSALYPLGEMRRSQRADGLA<br>AVLAIGTANPPNCVTQEEIPDFYFRVTNS<br>DHLTALKDKFKRICQEMGVQRRYLHHTEE<br>MLSAHPEFVDRDAPSLDARLDIAADAVPE<br>LAAEAAKKAIAEWGRPAADITHLVVTTNS<br>GAHVPGVDFRLVPLLGLRPSVRRTMLHL<br>NGCFAGCAALRLAKDLAENSRGARVLVV<br>AAELTLMYFTGPDEGCFRTLLVQGLFGDG<br>AAAVIVGADADDVERPLFEIVSAAQTIIPES<br>DHALNMRFTERRLDGVLGRQVPGLIGDN<br>VERCLLDMFGPLLGGDGGGGWNDLFWA<br>VHPGSSTIMDQVDAALGLEPGKLAASRR<br>VLSDYGNMSGATVIFALDELRRQRKEAAA<br>AGEWPELGVMMAFGPGMTVDAMLLHA<br>TSHVN	Synthesis of bisdemethoxycurcumin

Table S2. Yeast strains and plant lines present in work

Organism	Line name (ID)	Expressed genes
Yeast strains<br>*Pichia pastoris GS115*	ppas603	*nnLuz, nnH3H, Pv4CL1, AsPKS1*
	ppas604	
	ppas605	
	ppas608	*nnLuz, nnH3H, Pv4CL1, AsPKS2*
	ppas609	
	ppas610	
	ppas613	*nnLuz, nnH3H, Pv4CL1, HmS*
	ppas614	
	ppas615	
	ppas619	*nnLuz, nnH3H, Pv4CL1, AtPKSA*
	ppas620	
	ppas621	
	ppas623	*nnLuz, nnH3H, Pv4CL1, PpASCL*
	ppas625	
	ppas626	
	ppas802	*nnLuz, nnH3H, Pv4CL1, PcPKS3*
	ppas803	
	ppas804	
	ppas628	*nnLuz, nnH3H, Pv4CL1, RpBAS_L132S*
	ppas629	
	ppas630	
	ppas635	*nnLuz, nnH3H, Pv4CL1, EaPKS2*
	ppas636	
	ppas637	
	ppas638	*nnLuz, nnH3H, Pv4CL1, EaPKS3_mut*
	ppas639	
	ppas640	

	ppas797	*nnLuz, nnH3H, Pv4CL1, PzPKS2*
	ppas798	
	ppas799	
	ppas583	*nnLuz, nnH3H, Pv4CL1, PcPKS2*
	ppas584	
	ppas585	
	ppas598	*nnLuz, nnH3H, Pv4CL1, OsPKS*
	ppas599	
	ppas600	
	ppas588	*nnLuz, nnH3H, Pv4CL1, NtPKS*
	ppas589	
	ppas590	
	ppas593	*nnLuz, nnH3H, Pv4CL1, AhSTS*
	ppas594	
	ppas595	
	ppas578	*nnLuz, nnH3H, Pv4CL1, CalPKS1*
	ppas579	
	ppas580	
	ppas558	*nnLuz, nnH3H, At4CL1, AsPKS1*
	ppas559	
	ppas560	
	ppas563	*nnLuz, nnH3H, At4CL1, AsPKS2*
	ppas564	
	ppas565	
	ppas568	*nnLuz, nnH3H, At4CL1, HmS*
	ppas569	
	ppas570	
	ppas573	*nnLuz, nnH3H, At4CL1, AtPKSA*
	ppas574	

	ppas575	
	ppas643	*nnLuz, nnH3H, At4CL1, PpASCL*
	ppas644	
	ppas645	
	ppas247	*nnLuz, nnH3H, At4CL1, PcPKS3*
	ppas257	
	ppas258	
	ppas648	*nnLuz, nnH3H, At4CL1, RpBAS_L132S*
	ppas649	
	ppas650	
	ppas032	*nnLuz, nnH3H, At4CL1, RtSTS*
	ppas033	*nnLuz, nnH3H, At4CL1, WtPKS1*
	ppas034	*nnLuz, nnH3H, At4CL1, EaPKS1*
	ppas653	*nnLuz, nnH3H, At4CL1, EaPKS2*
	ppas654	
	ppas655	
	ppas658	*nnLuz, nnH3H, At4CL1, EaPKS3_mut*
	ppas659	
	ppas660	
	ppas031	*nnLuz, nnH3H, At4CL1, EaPKS3*
	ppas024	*nnLuz, nnH3H, At4CL1, PmSPS1*
	ppas025	*nnLuz, nnH3H, At4CL1, PmSPS2*
	ppas483	*nnLuz, nnH3H, At4CL1, PzPKS2*
	ppas484	
	ppas485	
	ppas222	*nnLuz, nnH3H, At4CL1, PcPKS2*
	ppas223	
	ppas224	
	ppas228	*nnLuz, nnH3H, At4CL1, OsPKS*

	ppas229	
	ppas230	
	ppas239	*nnLuz, nnH3H, At4CL1, NtPKS*
	ppas240	
	ppas241	
	ppas225	*nnLuz, nnH3H, At4CL1, AhSTS*
	ppas226	
	ppas227	
	ppas219	*nnLuz, nnH3H, At4CL1, CalPKS1*
	ppas220	
	ppas221	
	ppas336	*nnLuz, nnH3H, npgA, nnHispS*
	ppas337	
	ppas338	
	ppas671	*nnLuz, nnH3H, Pv4CL1*
	ppas012	*nnLuz, nnH3H, At4CL1*
	ppas000	*Wild type*
	ppas874	*npgA*
	ppas013	*At4CL1*
	ppas037	*npgA. nnHispS*
	ppas039	*At4CL1, AsPKS2*
	ppas040	*At4CL1, AtPKSA*
	ppas041	*At4CL1, PpASCL*
	ppas038	*At4CL1, HmS*
	ppas042	*At4CL1, PcPKS3*
	ppas043	*At4CL1, RpBAS_L132S*
	ppas1189	*At4CL1, PzPKS2*
	ppas044	*At4CL1, EaPKS2*
	ppas045	*At4CL1, PmSPS1*

	ppas046	*At4CL1, PmSPS2*
Plant stable lines<br>*Nicotiana benthamiana*	NB034	*nnLuz, nnH3H, nnCPH*
	NB218	*nnLuz, nnH3H, nnCPH, PpASCL*
	NB220	
	NB221	
	NB2359	*nnLuz, nnH3H, nnCPH, nnHispS, npgA*
	NB021	*nnLuz, nnH3H, nnCPH, nnHispS*

Table S3. Primers for genotyping

Primer name	Primer sequence
AsPKS1 dir	CACAAGCAGTACAACAAAATGT
AsPKS1 rev	GCATAAGCTTTCAAACTGCCAAGTTACGGGCC
AsPKS2 dir	AAGGCCGATGGTGATCTTAAG
AsPKS2 rev	GCATAAGCTTTCAAAGTGAACGAAGCAGAATGCC
HmS dir	CATTGATTGGAACTCTATCTTTT
HmS rev	GCATAAGCTTTCAAATGGGCACACTGTGCAACA
AtPKSA dir	GGCGACGAGTCCATGGAATT
AtPKSA rev	GCATAAGCTTTCATGATGAAGTAAGACTACGGATC
PpASCL dir	ATAACGATATTTTCTGGGCAGT
PpASCL rev	GCATAAGCTTTCAGCACAGGTTACGCAGCAAAG
PcPKS3 dir	ACTCTCTTTTCTGGATCGCAC
PcPKS3 rev	GCATAAGCTTTCAGTTAGCAACGGGAACAGAATG
RpBAS_L132S dir	CTCCCTTTTTTGGATCGCCC
RpBAS_L132S rev	GCATAAGCTTTCAAGAGATTACGGGTACTGAACG
RtSTS dir	CAGATTGGAACTCTTTGTTCTG
RtSTS rev	GAGCATtACTAGTTTAGTGATGGTGGTGGTGATGTGTGATCAGTGGCACGGAGGACAGG ACC
WtPKS1 dir	CTGATTGGAACTCCATTTTTTG
WtPKS1 rev	GAGCATACTAGTTTAGTGATGGTGGTGGTGATGAATTGACAAGGGTACGGAATGCAGCA CC
EaPKS1 dir	CGCATTCATCATCAACTTGGG
EaPKS1 rev	GAGCATACTAGTTTAGTGATGGTGGTGGTGATGATCACGGTTGATTGCAAAATGATGCA GTTG
EaPKS2 dir	CTTCTGTGAATGACATCTTTTG
EaPKS2 rev	GAGCATACTAGTTTAGTGATGGTGGTGGTGATGGCAAGCCAAACGGATTGATTTAAGCA GCA
EaPKS3_mut dir	CTTCCTATAATGATGTGTTTTGG

EaPKS3_mut rev	gcatAAGCTTtcaGTTGCAAAGATTGACAGACTTA
EaPKS3 dir	CATCCTATAATGACGTCTTTTGG
EaPKS3 rev	GAGCATACTAGTTTAGTGATGGTGGTGGTGATGGTTACAAAGGTTCACAGATTTAAGCAGGATAGTCTCC
PmSPS1 dir	CTCACCCCGGCGGCCC
PmSPS1 rev	GCATAAGCTTTCAAGACTTACCTGACCTTCC
PmSPS2 dir	AATAGCATATTCTGGACTGCTCA
PmSPS2 rev	GCATAAGCTTTCAGGACTTACCTGAATTGGC
PzPKS2 dir	GAAGTCTTTGGAGAACGGTC
PzPKS2 rev	gaGTTTACAACAGGGACACTG
AhSTS dir	GATTTGATGAGGAAGagaTCTC
AhSTS rev	gaTATGGCCACACTTCTAAGA
CalPKS1 dir	TGAGGAGGAAATCAACAAAAGA
CalPKS1 rev	gaAATAGCAATACTGTGCAACA
OsPKS dir	GAGGCAGAGGAAGGAGGC
OsPKS rev	gaATTCACATGAGAGGTGGC
PcPKS2 dir	GAGGTCACTTGAGAATGGTC
PcPKS2 rev	gaGTGTATAATAGGAACACTAC
NtPKS1 dir	GAGTACATGCGTGAAGAA
NtPKS1 rev	gaGAGGTTTCTCTCCAACAAT
Pich actin dir	GGTGTGGTGCCAGATCTTTT
Pich actin rev	AGTGTTCCCATCGGTCGTAG
At4CL1 dir	GATGTTGCTGTCGTTGCAATG
At4CL1 rev	tctcGAAGACAACAGAACCTGCGTAATCAGGTACGTCATA
Pv4CL1 dir	GTTGCTAAAGAAGTTGTATTCTAT
Pv4CL1 rev	gaATGTACCCCTGCGGCAA
nnH3H dir	GGTTTTGGTATGGGCCTG
nnH3H rev	agtcAAGCTTTCAGGCAGAATTAGAACTTCTTAG

nnLuz dir	ATCCCTAGCCACGACACTGT
nnLuz rev	gcatAAGCTTTCACTTGGCGTTTTCTACAATC

Table S4. Plasmids used in the study

Plasmid ID	Plasmid name	Constructs sequence
Yeast experiments		
pNK3404	pGap - At4CL1 - tAOX	https://benchling.com/s/seq-m6wrTzqsj8zicKydDerL?m=slm-kEEqCQBzIUDKwAHkHWJi
pNK3402	pGap - Pv4CL1 - tAOX	https://benchling.com/s/seq-BYql0xhY33kzIUK3ApIq?m=slm-chCt1JYHTwyNlCAaMHh5
pN001	pGap - AsPKS1 - tAOX	https://benchling.com/s/seq-TsxNcYba7SEi1IvCwYyN?m=slm-yv1WXh3PHTIl3EyTA1Tw
pN002	pGap - AsPKS2 - tAOX	https://benchling.com/s/seq-zx9ZAdB4jbGpDWovpN0n?m=slm-qpxRsZlYwAsMfv2l9jSL
pN003	pGap - HmS - tAOX	https://benchling.com/s/seq-pRRA8dmIaabhDHnPn1WS?m=slm-O3oCr0M0hIOAnzqoSa4r
pN004	pGap - AtPKSA - tAOX	https://benchling.com/s/seq-zlc88ZAIEk6SHGJPfaDr?m=slm-KJqrDES0hBpC6TIyzCRo
pN005	pGap - PpASCL - tAOX	https://benchling.com/s/seq-MLvwHwRKsBSlIP90XvEZ?m=slm-Z1n1OSOHuWY3lVtgzZBG
pN006	pGap - PcPKS3 - tAOX	https://benchling.com/s/seq-NDrkX1OvTENBsJW7go6X?m=slm-Suy8Uy0AtajleZJIB0r9
pN007	pGap - *RpBAS_L132S* - tAOX	https://benchling.com/s/seq-FUwEy2PEamAta77dt5xl?m=slm-w929JBhDF4gdClYLwt6y
pN008	pGap - *RtSTS* - tAOX	https://benchling.com/s/seq-uardiVquqYpZxkQINsam?m=slm-MLObZ8hzbySVaDTIRA6q
pN009	pGap - *WtPKS1* - tAOX	https://benchling.com/s/seq-SIFIMm1e2SjaVETyHvYp?m=slm-59ZY3zKKtkBoUkkIH48E
pN010	pGap - EaPKS1 - tAOX	https://benchling.com/s/seq-P9oSA9tyjA1GFCYmXJYL?m=slm-yYOD09OcpYjf8tCdfJet
pN011	pGap - EaPKS2 - tAOX	https://benchling.com/s/seq-cQvR138dBQUGxmegovUz?m=slm-PR8x4teEVfn2rLrZPNE3
pN012	pGap - EaPKS3_mut - tAOX	https://benchling.com/s/seq-IpZILPktjP0bil3bQ09O?m=slm-DfovDlMqTehOwdCJnIMc
pN013	pGap - EaPKS3 - tAOX	https://benchling.com/s/seq-vu7zDvGCUK8EJ4jFqEZj?m=slm-8IgcGZ6EtD4yJiM4jfDn
pNK5972	pGap - PmSPS1 - tAOX	https://benchling.com/s/seq-zPCE2sV7UVf1aeDXfGbZ?m=slm-K9dto17gVUl3OJY5ZGfC
pNK5973	pGap - PmSPS2 - tAOX	https://benchling.com/s/seq-Bpkz40lEPrCfMfK555lA?m=slm-iwJTmy5yGbDvtt6cdEtT
pNK1337	pGap - PzPKS2 - tAOX	https://benchling.com/s/seq-y4oyvfN7oOYe8U7AZRxt?m=slm-QNrmFzKmk3FdsvA9h8Jm
pNK1241	pGap - PcPKS2 - tAOX	https://benchling.com/s/seq-6rPjiT2AboVEBfMeREpS?m=slm-C3IvbXq54hGJiHeP3cbO
pNK1228	pGap - OsPKS - tAOX	https://benchling.com/s/seq-gAaLZaRSNyR82RMXE9O4?m=slm-1wsIFKFM7eoQZ08v0Rsr
pNK1338	pGap - NtPKS1 - tAOX	https://benchling.com/s/seq-AT1g8uRsEwgasKC25AIH?m=slm-lAKwWHNbS4AwTBfiDwHa

pNK1246	pGap - AhSTS - tAOX	https://benchling.com/s/seq-EkHBqsN4BWB8Vv1ZpsV3?m=slm-ClOAhjdmGqMahF9tVrSD
pNK1227	pGap - CalPKS1 - tAOX	https://benchling.com/s/seq-bHxKSdFnDwWasqJ5RfUn?m=slm-SALULca5FKIBrHmIG74L
pNK1507	pGap - nnHispS - tAOX	https://benchling.com/s/seq-OgA0nrEXqO4Cb9HIcoyb?m=slm-tWHZ2NoP6JFmuVz2SuTv
pNK1637	pGap - npgA - tAOX	https://benchling.com/s/seq-SWbHhDP60E0U1rULTq78?m=slm-oRXLoRlXCoNT7PaQGILV
pNK6701	pGap - FFluc - tAOX	https://benchling.com/s/seq-u8dbhWgpXTKi154SbxE1?m=slm-oKzc0RbC3EMDifoz3xgT
pNK6766	pGap - Nanoluc - tAOX	https://benchling.com/s/seq-MhVWrZpXt6HumM6YITEx?m=slm-y8xtkXWIIKn8SBIYdDNl
Plant experiments		
pNK062	L1-1 | pNOS - P19 - tOCS	https://benchling.com/s/seq-RYyvplUDlaBIZVyGhuxm?m=slm-eJqj3Gh7HJZV7f3othYM
pNK093	L1-4 | p35s_0.4kb - 5'UTR TMV omega - nnLuz - tOCS	https://benchling.com/s/seq-Ddvu5VZlYUM7Cv3GFBQp?m=slm-RqgtHGaw5xQi7uSfTtUr
pX030	L1-5 | p35s_0.4kb - 5'UTR TMV omega - CPH - tOCS	https://benchling.com/s/seq-pH45GUlQ6iwRHJebnzY0?m=slm-tw8bt88Z4BPTnGNZ8Wq9
pNK093	L1-6 | pFMV - nnH3H - tNOS	https://benchling.com/s/seq-UcmQVyvYAqPOIykpadhe?m=slm-A14cAcGZy3Hk8XHKkER0
pNK069	L1-3 | pCmYLCV - At4CL1 - tATP	https://benchling.com/s/seq-SWdIqrsb34LhecB56Fqb?m=slm-jnr9GHCVadY1FtFloQ2T
pNK276	L1-2 | p35s_0.4kb - 5'UTR TMV omega- AsPKS1 - tOCS	https://benchling.com/s/seq-sYhLTULbprTUEoOEbIxL?m=slm-L4w5Hs0yUoMPpyXcEQsl
pNK277	L1-2 | p35s_0.4kb - 5'UTR TMV omega- AsPKS2 - tOCS	https://benchling.com/s/seq-YVQLBHvYarVnQRluLpB8?m=slm-SOHwz7PrG6BTuHqfmQLk
pKB147	L1-2 | p35s_0.4kb - 5'UTR TMV omega- HmS - tOCS	https://benchling.com/s/seq-hWUU2sN20C0OzEeybNE8?m=slm-KOlvhPAyhoLxoXN4sp0U
pNK306	L1-2 | p35s_0.4kb - 5'UTR TMV omega- AtPKSA - tOCS	https://benchling.com/s/seq-chCq17w2zPd90xsgwOMM?m=slm-LM6ZpjjOhYF92Lp9QDBb
pNK657	L1-2 | p35s_0.4kb - 5'UTR TMV omega- PpASCL - tOCS	https://benchling.com/s/seq-0BydnO38KrevKMyhhWx1?m=slm-VxQxfLELzEdVX1C1ZVx6
pKB148	L1-2 | p35s_0.4kb - 5'UTR TMV omega- PcPKS3 - tOCS	https://benchling.com/s/seq-0zUAAWXO4X4UClxACwl4?m=slm-OMdSQdC8qmBBUTa3sNh4
pNK282	L1-2 | p35s_0.4kb - 5'UTR TMV omega- RpBAS_L132S - tOCS	https://benchling.com/s/seq-bBw141tDvlZoQUHljaYe?m=slm-vQeXMloHVDFUdAVjrWmZ
pKB149	L1-2 | p35s_0.4kb - 5'UTR TMV omega- EaPKS2 - tOCS	https://benchling.com/s/seq-5rRJb76gP4MN9Y0IoHZO?m=slm-Cb3Vba1hKynV1F8P7EaM
pNK891	L1-2 | p35s_0.4kb - 5'UTR TMV omega- CalPKS1 - tOCS	https://benchling.com/s/seq-qjhQRQgDVQUTnjp7xByM?m=slm-dODMfjOT200xc2bFX7B9
pNK625	L1-2 | p35s_0.4kb - 5'UTR TMV omega- NtPKS1 - tOCS	https://benchling.com/s/seq-43OWdLnujKNA4q7prx1f?m=slm-kELSBFK5eaVDLhRVNDm8

pNK624	L1-2 | p35s_0.4kb - 5'UTR TMV omega- AhSTS - tOCS	https://benchling.com/s/seq-TwFubLjEBYmigJEJm7id?m=slm-WURymbBN4nOHeb6SOBy4
pNK1028	L1-2 | p35s_0.4kb - 5'UTR TMV omega- PzPKS2 - tOCS	https://benchling.com/s/seq-iTVNB6gU30Kvnj6HHX5s?m=slm-JcnST5wL40IAn1rGuKqu
pNK622	L1-2 | p35s_0.4kb - 5'UTR TMV omega- PcPKS2 - tOCS	https://benchling.com/s/seq-ghOucIV8b42KGgx1WrO5?m=slm-Wl2DK4xgWOnpPLOM8S1s
pNK474	LM-2 | p35s_0.4kb - 5'UTR TMV omega- PpASCL - tOCS | pNOS - HygrR - tOCS	https://benchling.com/s/seq-Jl36TWwqEaGIXl1NsfML?m=slm-IkYCNnY2Csc0jK3jUXqu
pX028	L1-2 | p35s_0.4kb - 5'UTR TMV omega - nnHispS - tOCS	https://benchling.com/s/seq-RZSqnGarVOTfY6YkQwbi?m=slm-nDRWeULaMf3D0lv55JOm
pNK077	L1-3 | pCmYLCV - npgA - tATP	https://benchling.com/s/seq-KnaONbefkXDrg8iLcMkn?m=slm-pNyGIuy4jFiIDFOd0an7
pNK5980	L1-5 | p35s_0.4kb - 5'UTR TMV omega - nnLuz sense - PDK intron - nnLuz antisense - tOCS	https://benchling.com/s/seq-2nObY4Jdz498jLMPrtJt?m=slm-H4eyZKj3V1kqonykOsMJ
pNK6012	L1-5 | p35s_0.4kb - 5'UTR TMV omega - nnHispS sense - PDK intron - nnHispS antisense - tOCS	https://benchling.com/s/seq-ytmsZP6tKkwbTKr58l3A?m=slm-au1euzV0WfmqCZ7JKqtu
pNK5902	L1-5 | p35s_0.4kb - 5'UTR TMV omega - C4H sense - PDK intron - C4H antisense - tOCS	https://benchling.com/s/seq-SBTvLcnAUj1NelUoytZ9?m=slm-kBZciTIt25JesJDLHp3V
pNK5879	L1-5 | p35s_0.4kb - 5'UTR TMV omega- CHI sense - PDK intron - CHI antisense - tOCS	https://benchling.com/s/seq-vd7HabvaUOzvAdGs1shT?m=slm-o1KJquFFUgATfwArenIK
pNK5881	L1-5 | p35s_0.4kb - 5'UTR TMV omega - CHIL sense - PDK intron - CHIL antisense - tOCS	https://benchling.com/s/seq-mbTbbzSY77k1lWXGJTwE?m=slm-CdA7IHSjH6CYUtD8rVdT
pNK5965	L1-5 | p35s_0.4kb - 5'UTR TMV omega - CHIL3 sense - PDK intron - CHIL3 antisense - tOCS	https://benchling.com/s/seq-T2fjVRJHPiqvvIc9Dd0p?m=slm-jFZYuR0ek3RZkRK5E1bt
pNK5882	L1-5 | p35s_0.4kb - 5'UTR TMV omega - F3H sense - PDK intron - F3H antisense - tOCS	https://benchling.com/s/seq-DxiFcyYXTzsPOfqs39r3?m=slm-MW7E7WUoEwO45D4EL0hh
pNK5884	L1-5 | p35s_0.4kb - 5'UTR TMV omega - P450 sense - PDK intron - P450 antisense - tOCS	https://benchling.com/s/seq-dPpxxG8nDYktYt0FYKT2?m=slm-bkL30AFK3no3kvZLiJWm
pNK5958	L1-5 | p35s_0.4kb - 5'UTR TMV omega - PAL4 sense - PDK intron - PAL4 antisense - tOCS	https://benchling.com/s/seq-YnDYCFYFBSSTcHKlwd4u?m=slm-Ri2VbEMqixUzL98OnOG5
pNK806	L1-3 | p35s_0.4kb - 5'UTR TMV omega - AAE13 - tOCS	https://benchling.com/s/seq-5ZJ8Vz1GSTdN5hWZMmxv?m=slm-AbxhOPopqVpBJsEgPeRS
pNK743	L1-3 | p35s_0.4kb - 5'UTR TMV omega - AtC4H (CYP73A5) - tOCS	https://benchling.com/s/seq-Qw4fR0WJL4Qv1Hync26z?m=slm-bxuqbJblZf0KXLlkAh20
pNK5759	L1-3 | p35s_0.4kb - 5'UTR TMV	https://benchling.com/s/seq-njw1NKC1aMr3v9w3FiqQ?m=slm-sWUx9CBZcSc9s40bqSDh

	omega - GmCHIL - tOCS	
pNK5761	L1-3 | p35s_0.4kb - 5'UTR TMV omega - MdCHIL - tOCS	https://benchling.com/s/seq-hBpOuXJY3zuKVnb8aOCF?m=slm-3DtziJe78bV75RE3WI7e
pNK5744	LM-4 | p35s_1.4kb - nnLuz - tAct2 | p35s_0.4kb - nnCPH - tOCS | pFMV - nnH3H - tNOS	https://benchling.com/s/seq-b4yEOjLZVRcBDon6n9xI?m=slm-FbK7bF66bkikMVpbZHQR
pNK328	LM-2 | p35s_0.4kb - 5'UTR TMV omega - nnHispS - tOCS | pCmYLCV - npgA - tATP	https://benchling.com/s/seq-dSatuz4vY5VHAhQ6SHy6?m=slm-O2kdYoG6fjtqSHdNC9ja
pNK6062	(GB2085) dCasEV	Plasmids from ref (*30*) (https://gbcloning.upv.es/search/)
pNK6063	(GB2070) Non-target gRNA2.1	
pNK6064	(GB2389) NbPAL1 gR1	
pNK6065	(GB2396) NbPAL2 gR1	
pNK6066	(GB2760) NbCL4 gR1	
pNK6067	(GB2390) NbCHS1 gR1	
pNK6068	(GB2397) NbCHS2 gR2b	
pNK6069	(GB2599) NbCHS2 gR2ab	
pNK6070	(GB2502) NbCHI1 gR1	
pNK6071	(GB2391) NbCHI2 gR1	
pNK6072	(GB2503) NbCHI2 gR2	
pNK6073	(GB2392) NbF3H gR1	
pNK6074	(GB2531) NbF3'H gR1	
pNK6075	(GB2395) NbC4H gR1	
pNK6212	(GB2170) NbDFR gR1	
pNK6213	(GB2394) NbFLS1 gR1	
pNK6214	(GB2500) NbCHS2 gR2a	
pNK6215	(GB2530) NbFLS2 gR2	
pNK6216	(GB2639) NbFLS1 gR2	
pNK6218	(GB2863) AP-N2B	
pNK6219	(GB2864) AP-N2	
pNK6220	(GB2866) AP-N4	
pNK6221	(GB2776) AP-N1B	

pNK6222	(GB2777) AP-N1	
pNK6223	(GB3242) AP-E	
(GB4926) p35s-PzPKS2-tNos		https://benchling.com/s/seq-BB6FtXKz2HOOyREpSco4?m=slm-1ee978fjiQfot6iQTWY6
(GB4925) p35s-PpASCL-tNos		https://benchling.com/s/seq-D8CcAtpWVL45X4to3Gv6?m=slm-lNEWcSWj9p3QktYbTWZf
(GB4924) p35s-HmS-tNos		https://benchling.com/s/seq-z5UZn4e714gBE0n6sYbI?m=slm-sfzcj7qEuqiArcKx8Vo5
(GB3475) p35s-nnHispS-tNos		https://benchling.com/s/seq-0kgZNmWUGmsJ8vzzVQgn?m=slm-KX51mzSn2cl09bN88lF3
(GB4485) p35s-nnCPH-tNos + p35s-nnLUZ-tNos + p35s-nnH3H-tNos + p35Ss-eGFP-tNos + p35s-p19-tNos		https://benchling.com/s/seq-Ay779tOPziPYlFi1LZpk?m=slm-pqwxZYU3NpQhaiVPwp6O
(GB4401) pNos-nnHispS-tNos + p35s-nnCPH-tNos + p35s-nnLUZ-tNos + p35s-nnH3H-tNos + p35Ss-eGFP-tNos + p35s-p19-tNos		https://benchling.com/s/seq-fUADE2y6kV1GkLWBs9ug?m=slm-yo9pOrmUKWln0QBorRTZ
pNK6451	(GB5060) BeYDV-PzPKS2	https://benchling.com/s/seq-e1bnlO80oUK3suw3mGz6?m=slm-NUuHFSiACyPoBFozaRPz
pNK6448	(GB5058) BeYDV-PpASCL	https://benchling.com/s/seq-V8kqHdsWuuEFmRLNPXTw?m=slm-T4i4qao093ju2RogZDr0
pNK6449	(GB5059) BeYDV-HmS	https://benchling.com/s/seq-x3iAUmgv4mKwsSs5gAGW?m=slm-vCCcbuazWLMucgzPVtf3
pNK6442	(GB3881) BeYDV-nnHispS	https://benchling.com/s/seq-cevoWpx9zPgLpvlJuTLj?m=slm-mQvHxCe16WiMpYyblO8d
pNK6443	(GB3598) pNos-Rep/RepA-tNos	https://benchling.com/s/seq-ktdr0uyCdzPyIoAwP0A2?m=slm-TzqTLJ9jqMPyI1h7dcdL
pNK6445	(GB5098) TMV-PzPKS2	https://benchling.com/s/seq-ikFZtoJMKrQmOkNUVM68?m=slm-Ptoz99tb8gFq5EosyBfz
pNK6444	(GB5097) TMV-PpASCL	https://benchling.com/s/seq-LA7E3meiUwx0Er7EzNIB?m=slm-eG08sytIIPSJkiFKenP3
pNK6467	(GB5096) TMV-HmS	https://benchling.com/s/seq-rpTiB0tpfo3er9jXX0b3?m=slm-wAGeTACU1qxvP8sdRe9x
pNK6453	(GB5095) TMV-nnHispS	https://benchling.com/s/seq-Y1UryKgC2YdZkzuWVsxT?m=slm-SGRBfDTydb15U28CIYfD
	(GB0106) Negative control	https://benchling.com/s/seq-3V8Paz833MXNrUFxb2Ll?m=slm-6TV3cD7l5OsTzfN73OGl
pNK6260	L1-2 | p35s_0.4kb - 5'UTR TMV omega- FFLuc- tOCS	https://benchling.com/s/seq-p9DhYLhq19tb7w0WByGJ?m=slm-aJDvpOuOCnqmlYZoirfh
pNK6269	L1-2 | p35s_0.4kb - 5'UTR TMV omega- nanoluc - tOCS	https://benchling.com/s/seq-q8457Sl7OXMulQ5JB0BV?m=slm-CkGvdi7lMlnoz7VrsZgp
Mammalian experiments		
pN021	pCMV - nnLuz - 3'UTR_SV40	https://benchling.com/s/seq-Z37IB4oDW0iVBjzLEFnn?m=slm-9Mco8HsoEpDGdl3vnkUe
pN022	pCMV - nnH3H - 3'UTR_SV40	https://benchling.com/s/seq-X0HAsCliT6dIOOgyJFLS?m=slm-t2rFiH1c649ABy3yabRk
pX158	pCMV - At4CL1 - 3'UTR_SV40	https://benchling.com/s/seq-RVUuDNc3Bdpht6YCuiTN?m=slm-t4n6vYXyL4Pquq7LAoOU

pNK2917	pEF1α - At4CL1 - 3'UTR_BGH	https://benchling.com/s/seq-OzOADh5AMM74WKNjmBqF?m=slm-vkoNMm5i2jJDZeOfZAJq
pNK2907	pEF1α - At4CL2 - 3'UTR_BGH	https://benchling.com/s/seq-J2kRS7oGIT8gClbL4cga?m=slm-R8cBGpxzVMwgESc6Erbo
pNK2915	pEF1α - Nt4CL2 - 3'UTR_BGH	https://benchling.com/s/seq-D4bBEu7q8U4S0K2YXuCN?m=slm-bUZOJajPRdx188KBfew8
pNK2913	pEF1α - Pv4CL1 - 3'UTR_BGH	https://benchling.com/s/seq-NyTHD4b0PNOfnjM6HIOT?m=slm-gqXPdvGLXmOgeEJAqOIA
pX137	pCMV - npgA - 3'UTR_SV40	https://benchling.com/s/seq-RpulkKBJlWumFOqDylqa?m=slm-aHyh7E5aGOEbnmpn4Xqn
pNK4292	pCMV - PzPKS2 - 3'UTR_SV40	https://benchling.com/s/seq-DiDfruPE08ATt8MYEJnt?m=slm-7p87KPAfLrH9ACBVVrOc
pX156	pCMV - HmS - 3'UTR_SV40	https://benchling.com/s/seq-BSJClJaulWx6JOhtyi1B?m=slm-fMb3XJF7YqSMKsD3hElq
pX157	pCMV - AtPKSA - 3'UTR_SV40	https://benchling.com/s/seq-1vZYXt4Zt7uY24igBHr2?m=slm-VdrwYJkjO8MGHRLJcnvs
pNK2896	pCMV - CalPKS1 - 3'UTR_SV40	https://benchling.com/s/seq-4lm66JuyEdzjcod81unT?m=slm-fzcO4MxDpidtCrhKyb1j
pNK2898	pCMV - AhSTS - 3'UTR_SV40	https://benchling.com/s/seq-WvvT5kFWJ3ZiUSyqZWfU?m=slm-0Gyg8H7nDjvZNS8ZwyVC
pNK2901	pCMV - PcPKS3 - 3'UTR_SV40	https://benchling.com/s/seq-uHhtFw8dw1xIzqpVvUdj?m=slm-23vt29c00QOqGKjrJs3i
pNK2905	pCMV - NtPKS1 - 3'UTR_SV40	https://benchling.com/s/seq-BE3Us73tN5rA7yzwIUen?m=slm-Bq1N4qYma4peclVLt53D
pNK6037	pCMV - PpASCL - 3'UTR_SV40	https://benchling.com/s/seq-FEteabmI0kaeMIME65hk?m=slm-DOLGVY3YoAU9lYDmjyON
pNK6039	pCMV - RpBAS_L132S - 3'UTR_SV40	https://benchling.com/s/seq-qYMxKnAQlbmEyENUAOTO?m=slm-xgoXXhjUjzccoeJfsJj1
pNK6038	pCMV - PcPKS2 - 3'UTR_SV40	https://benchling.com/s/seq-k0Bg3wdPzL7r2EKNiCYP?m=slm-OktgV9zxnnvaX55Mlaiu
pX135	pCMV - nnHispS - 3'UTR_SV40	https://benchling.com/s/seq-nI1eb986Zxk0sZGVEMDt?m=slm-gm1MFkhaitfObtxbcDca
